# Manipulating Backbone Planarity of Ester Functionalized Conjugated Polymer Constitutional Isomer Derivatives Blended with Molecular Acceptors for Controlling Photovoltaic Properties

**DOI:** 10.1021/acs.chemmater.4c02751

**Published:** 2024-11-26

**Authors:** Sina Sabury, Austin L. Jones, Nora Schopp, Sadisha Nanayakkara, Thomas P. Chaney, Veaceslav Coropceanu, Seth R. Marder, Michael F. Toney, Jean-Luc Brédas, Thuc-Quyen Nguyen, John R. Reynolds

**Affiliations:** † School of Chemistry and Biochemistry, School of Materials Science and Engineering, Center for Organic Photonics and Electronics, Georgia Tech Polymer Network, Georgia Institute of Technology, Atlanta, Georgia 30332, United States; ‡ Center for Polymers and Organic Solids, Department of Chemistry and Biochemistry, University of California Santa Barbara, Santa Barbara, California 93106, United States; § Department of Chemistry and Biochemistry, The University of Arizona, Tucson, Arizona 85721-0041, United States; ∥ Materials Science and Engineering Program, University of Colorado, Boulder, Colorado 80309, United States; ⊥ Department of Chemical and Biological Engineering, University of Colorado, Boulder, Colorado 80309, United States; # Renewable and Sustainable Energy Institute, University of Colorado, Boulder, Colorado 80303, United States

## Abstract

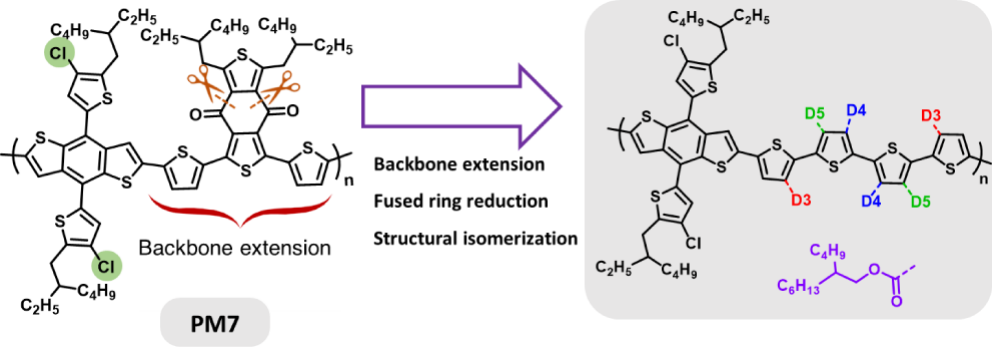

Exploring both electron donor and acceptor phase components in bulk heterojunction structures has contributed to the advancement of organic photovoltaics (OPV) realizing power conversion efficiencies reaching 20%. Being able to control backbone planarity of the donor polymer, while understanding its effects on the polymer conformation and photophysical properties, fosters the groundwork for further achievements in this realm. In this report, three isomeric PM7 derivatives are designed and synthesized where the benzodithiophene-4,8-dione structure is replaced by a quaterthiophene bridge carrying two ester moieties. The placement of these two ester groups varies among three configurational isomers, which ultimately influences the chain conformations and aggregation behavior of each polymer. Specifically, PM7-D3 has ester groups attached to the inner positions of the outer thiophenes showing moderate solution aggregation; PM7-D4 has ester groups attached to the inner positions of the inner thiophenes featuring a twisted backbone with no solution aggregation behavior; and PM7-D5 has ester groups attached to the outer positions of the inner thiophenes with strong solution aggregation. PM7-D5 shows the highest average power conversion efficiency of 11.4% paired with the molecular acceptor L8-BO. In addition, the differences among the polymer backbones are expressed by their state energies and carrier mobility in the corresponding fabricated OPV devices.

## Introduction

Impressive improvements in the power conversion efficiency (PCE), reaching 20%,^1^ have incentivized efforts toward providing an in-depth understanding of the complex processes involved in using new donor and acceptor phase materials in polymer-based organic photovoltaics (OPV). As illustrated by the repeat unit structures and acronyms in Figure S1, the original donor polymers paired with fullerene-based acceptors used in bulk heterojunction (BHJ) morphologies have advanced from poly(3-hexylthiophene) (P3HT) to include many donor–acceptor (D–A type) polymers. These systems, including PTB7-Th and PCE11, broke the 10% PCE threshold by producing a favorable BHJ morphology.^2−6^ Further, molecular acceptors (MAs), also referred to as nonfullerene acceptors (NFAs), with a set of structures shown in Figure S1, have been instrumental in the improvement of performance of OPVs since their introduction in 2015,^7^ eventually surpassing 15% PCE in 2019.^8^ While progress in MAs have continued to lead the way, development of donor polymers with varied electronic-state energies, backbone conformations, and packing modes are required to extend the field further.

**Scheme 1:**
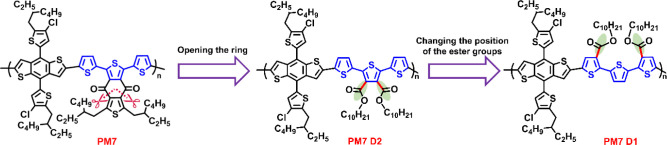
Derivation of PM7 D1 and PM7 D2 from PM7 Based on Open-Ring Structure–Schematic Scissors Cutting Two Bonds Replacing the BDD Unit with a Terthiophene Bridge with Two Ester Functional Groups and Altering the Placement of the Ester Groups (Isomerization) in Our Previous Works Adapted with permission from ref (17). Copyright 2023 American Chemical Society.

As highlighted in Figure S1, many of the higher performing donor phase polymers in BHJ OPVs are based on benzo[1,2-b:4,5-b′]dithiophene (BDT) as the donor unit and benzodithiophene-4,8-dione (BDD) as the acceptor and make up the PBDB-T family of polymers. Namely, PM6 (also referred to as PBDB-T-2F) became a benchmark donor polymer when paired with various MAs, with solar cells reported to be performing at 15.7% when the polymer is paired with Y6 as the acceptor.^8,9^ Ternary blends and interfacial engineering using self-assembled monolayers (SAMs) have also been used to reach even higher PCEs of 20%.^1,10−12^ The chlorinated derivative of PM6, named as PM7 (PBDB-T-2Cl), owing to the absence of three synthetic steps enabled by replacement of fluorine for chlorine, is a move toward scalability even though the PM7:Y6 binary system performs at a slightly lower PCE of ∼14.4%.^13,14^ Moving toward the polymers in the present work, the Reynolds group^15−17^ developed two PM7 derivatives shown in Scheme 1, PM7 D1 and PM7 D2, in which the BDD fused ring was opened, shown schematically as scissors cutting two bonds, to provide a structure having a terthiophene with two ester functional groups. PM7 D1, for example, when used in a binary mixture with ITIC-4F provided a PCE of 12.1%. More importantly, it was shown that this opened-ring design simplifies the synthesis scheme compared to the BDD-based homologue (PM7) by reducing synthetic steps. Further, backbone flexibility was increased via rotations around the thiophene linkages, which enhanced the mechanical robustness, such as crack onset strain,^16^ and led to active layer thickness tolerance;^15^ also, that flexibility promoted network-like aggregation, found to be resilient toward variations in processing temperature while retaining OPV performance.^17^ Batch-to-batch variation and dependency of device performance on the molecular weight are among other factors positively modified by the opened-ring design.^18^ While the conventional design paradigm for conjugated polymers has mostly been explored based on developing rigid and planar π-conjugated systems, evident by the repeat unit structures in Figure S1, studies have suggested excess rigidity of the backbone can frustrate proper packing^19^ and some degree of backbone flexibility is required to accommodate the favorable phase separation in BHJ OPVs.^20,21^ Additionally, backbone flexibility based on the open-ring structures is targeted to improve the processability of these systems, such as the ability to process from nonhalogenated solvents.^22,23^

**Figure 1:**
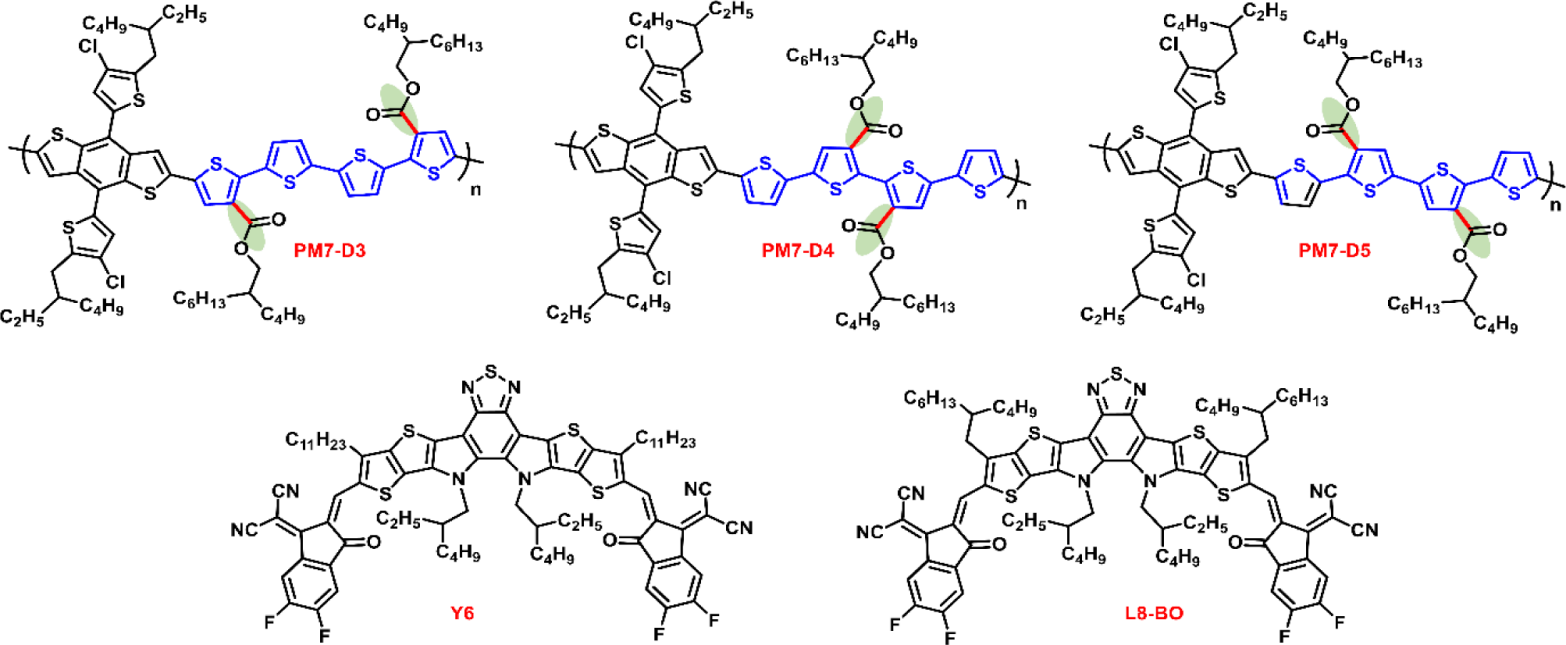
Extension of opened-ring PM7 constitutional isomeric derivatives based on quaterthiophene bridges paired with Y6 and L8-BO as molecular acceptors.

In this report, we extend the approach of reducing fused-ring content using quaterthiophenes that contain two ester functionalities to create three constitutional isomeric donor polymers derived from PM7. This allows fine-tuning of the backbone conformation, aggregation properties, and control of the ionization potentials based on the placement of the two ester moieties. As shown in Figure 1, the placement of the ester side chains differs among these three derivatives: PM7-D3 has ester groups attached to the inner positions of the outer thiophenes, PM7-D4 has ester groups attached to the inner positions of the inner thiophenes, and PM7-D5 has ester groups attached to the outer positions of the inner thiophenes. These polymers show three distinct aggregation and packing properties, ranging from nonaggregating in solution and poorly packing in films (PM7-D4), to nonaggregating in solution yet showing enhanced ability to pack in the solid state (PM7-D3), to aggregating in solution in a way that resembles solid-state packing (PM7-D5). Solution preaggregation is acknowledged as a contributor to the BHJ morphology formation and device performance.^6,24−28^ The ionization energies vary over a range of 280 meV from 5.55 to 5.83 eV, providing an opportunity to match with the electronic-state energies of various MAs. Long-range corrected density functional theory (DFT) calculations confirmed the placements of the ester groups planarize PM7-D3 and PM7-D5, while creating twists along PM7-D4’s backbone. These polymers (PM7-D*x*, *x* = 3, 4, 5) paired with Y6 lead to markedly different OPV performances with PM7-D5′s PCE at 8.9%, PM7-D4’s at 3.9%, and PM7-D3′s at 5.9%. Devices made with another MA, referred to as L8-BO^29,30^ (chemical structure displayed in Figure 1), shows the same trend among the polymers with PM7-D5 performing as high as 11.4% followed by PM7-D3 and then PM7-D4 at lower PCEs. The significant difference between the OPV devices allowed us to compare PM7-D4 and PM7-D5 in more depth. Impedance spectroscopy measurements demonstrated that the performance of the PM7-D4:L8-BO blend is not limited by charge generation processes, nor by bulk or interface traps. Time-dependent (TD) DFT calculations indicated a lack of electronic couplings between the local exciton state and charge transfer state in the PM7-D4 conformer, resulting in limited exciton dissociation. On the other hand, efficient exciton dissociation and higher effective mobility in PM7-D5 allow it to perform at the highest PCE whereas PM7-D4:L8-BO blend suffers from large acceptor domains, reduced D:A interfacial area, and low mobility leading to low PCE (2.1%). As a result of this effort, the role of constitutional isomerization on backbone conformation, state energies, aggregation, and packing, which affect charge generation processes and therefore the performance in a BHJ single junction OPV system is discussed.

## Results and Discussion

### Synthetic Approach

Three structurally isomeric quaterthiophene-based dibromo comonomers containing two ester functionalities (see Figure S2 for molecules **4**, **8**, and **14**) were synthesized with their synthesis schemes and molecular characterization described in the Supporting Information(Figures S3–S34). Branched butyl-octyl side chains on the esters are incorporated to increase solubility of the polymer in the organic solvents used for Stille coupling polymerization and processing; indeed, we had observed solubility limitations (solubility in chloroform of less than 1 mg/mL) when linear decyl side chains were used in our initial synthesis of these quaterthiophene-based polymers. As summarized in Table S1, all polymers were obtained in excellent yields (93–98%) after Soxhlet purification. Based on high-temperature gel permeation chromatography (HT-GPC) results at 140 °C using 1,2,4-trichlorobenzene as the eluent solvent, the number-average molecular weights (*M*_n_) of the polymers are found to be 64.6 kg/mol, 64.3 kg/mol, and 26.1 kg/mol for *M*_n_ of PM7-D3, PM7-D4, and PM7-D5 respectively. As the HT-GPC chromatograms show, all polymers are monomodal with a dispersity (*Đ*) in the range of 2.1–2.6 (see Figure S35 for HT-GPC chromatograms). A separate study of the dependency of the OPV performance on molecular weight for PM7-D5 (comparing *M*_n_ of 26.1 kg/mol and 125 kg/mol) indicated a minimal molecular weight dependency for this polymer; thus, we can corroborate that the differences within the quaterthiophene derivatives arise from their repeat-unit structural differences rather than variations in molecular weights and molecular-weight distributions.^18^ Being constitutional isomers, the elemental composition of these three polymers is the same and the elemental analysis of carbon, hydrogen, and sulfur supports the high purity of the polymers within the detection limit (0.3%) of the analysis using the combustion method.

### Geometry, Electronic Structure, and Optical Properties

**Figure 2:**
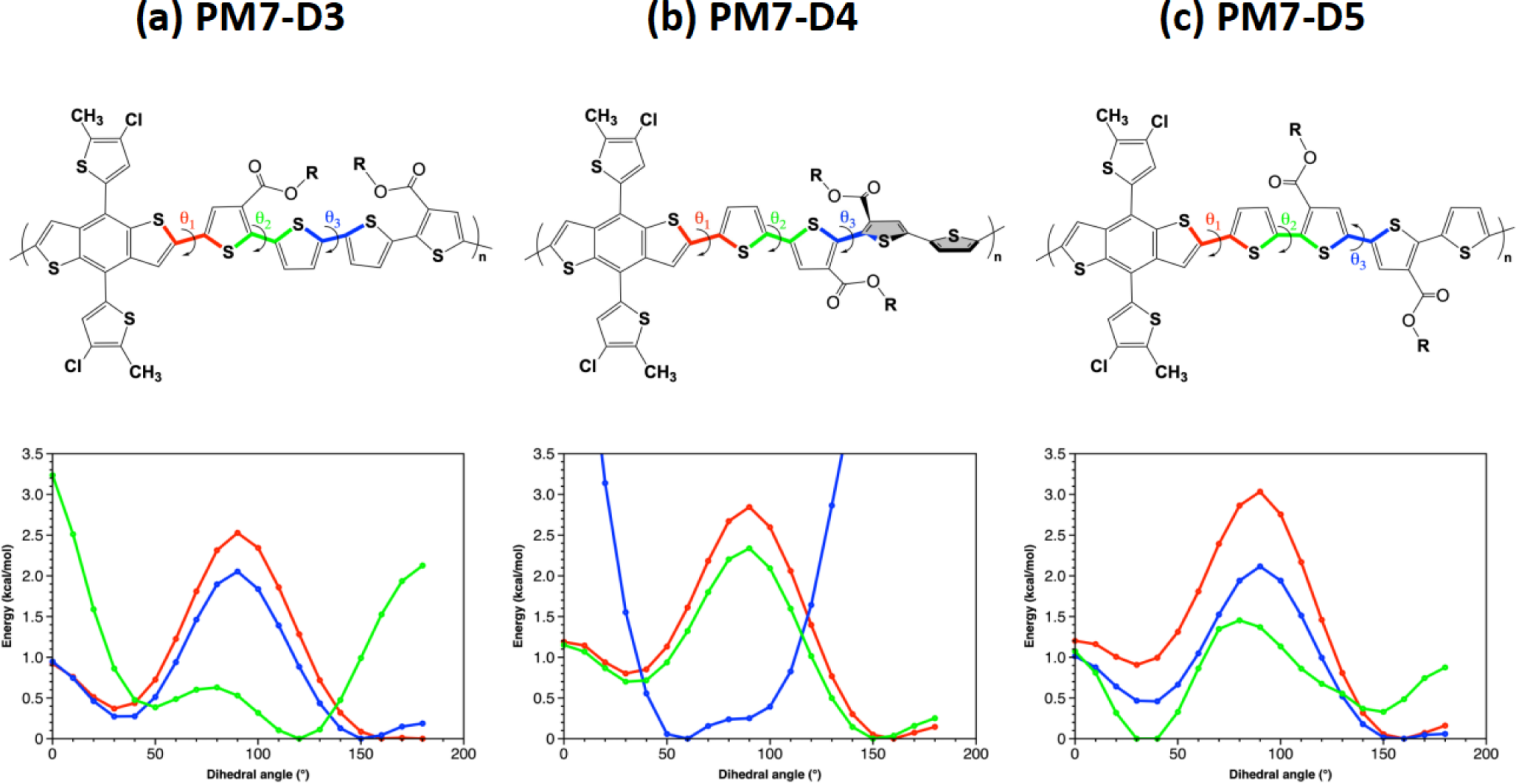
(a) PM7-D3, (b) PM7-D4, and (c) PM7-D5. Top panel: Illustrations of the backbone conformations within one repeat unit of the symmetric oligomers and labeling of dihedral angles θ_1_ (in red), θ_2_ (in green), and θ_3_ (in blue) at different positions along the chain (R denotes full-length side chains). Bottom panel: Inter-ring torsion potential scans of the specific dihedral angles with truncated side chains, as calculated at the ωB97X-D/6-31G(d,p) level of theory.

To investigate the impact of side-chain placement on the backbone planarity of these conformational isomers at the DFT level, we modeled the PM7-D3, PM7-D4, and PM7-D5 polymers with symmetric, A:D:A:D:A, 18-ring oligomers; here, D stands for benzodithiophene and A, for quaterthiophene segments, respectively. The most stable structures obtained from geometry optimizations performed on isolated oligomers (see the computational methodology section in the Supporting Information for more details) are illustrated in Figure 2, with the corresponding dihedral angles reported in Table 1. We note that these results account for the influences of full-length side chains. Three key dihedral angles (θ_1_, θ_2_, and θ_3_) within the repeat units of each PM7-D*x* polymer (*x* = 3, 4, 5) were analyzed; these are the angle between BDT and the quaterthiophene group, the angle between the outermost thiophene and the inner bithiophene within a quaterthiophene segment, and the angle between the two inner thiophenes within a quaterthiophene segment (see Figure 2). Here, syn-orientation denotes the arrangement of neighboring thiophene rings in which the sulfur atoms are oriented in the same direction (or at the closest interacting distance), while anti-orientation indicates a conformation where the sulfur atoms face opposite directions (or at the furthest interacting distance).

**Table 1:** DFT/ωB97X-D/6-31G(d,p) Results on PM7-D3, PM7-D4, and PM7-D5 Oligomers^a^

Polymer	QT unit	θ_1_ (deg)	θ_2_ (deg)	θ_3_ (deg)	IP (eV)	EA (eV)	S_1_ (eV)
PM7-D3	1	163.5	154.0	24.1	5.20	2.14	2.51
	2	172.4	145.7	12.5			
	3	N/A	144.0	24.9			
PM7-D4	1	146.2	124.0	99.0	5.26	1.95	2.71
	2	152.2	132.0	86.7			
	3	N/A	150.5	69.0			
PM7-D5	1	159.0	31.2	162.5	5.14	2.13	2.46
	2	168.5	31.4	157.4			
	3	N/A	35.4	153.8			

a Dihedral angles θ1, θ2, and θ3 (deg) are reported for each of the quaterthiophene (QT) units in the backbone (three units per backbone, the QT unit number represents values for each QT unit). Also, ionization potential (IP), electron affinity (EA), and energies of the first singlet excitation (S_1_) are reported for each conformation observed for PM7-D3, PM7-D4, and PM7-D5 (see computational methodology section for derivation of these parameters). All energies are in eV.

It can be seen that PM7-D3 exhibits a relatively planar backbone, with average dihedral angles of anti-oriented thiophenes varying between 168° (±6°) for θ_1_ and 148° (±5°) for θ_2_ (standard deviation in parentheses) along with a pair of syn-oriented thiophenes having an average θ_3_ dihedral angle of 21° (±7°). In contrast, PM7-D4 displays a strongly twisted backbone (see gray colored ring segment in Figure 2, top panel) where θ_3_ fluctuates around 90° (±15°), deviating significantly from planarity. It should be mentioned that an alternative conformation (not reported here) is accessible to PM7-D4 with a higher energy (<1 kcal/mol per repeat unit) and exhibits θ_3_ around 60°, resulting in a somewhat less twisted backbone. For PM7-D5, a markedly more planar backbone is observed, with θ_1_ and θ_3_ of anti-oriented thiophenes typically varying around 160° (±4–7°) and θ_2_ of syn-oriented thiophenes about 33° (±2). Thus, our DFT calculations underline reduced torsional angles in PM7-D3 and PM7-D5; this is related to the positioning of the ester groups, which in turn adversely affects the PM7-D4 backbone resulting in a twisting deformation.

Additionally, we performed inter-ring torsion potential energy scans (see Figure 2, bottom panel) for the three dihedral angles of interest, this time using truncated models of the optimized oligomers with the side chains reduced to methyl groups. As deduced by the minima associated with each torsional curve, which point to the dihedrals of low-energy conformers, backbone planarity is exhibited in PM7-D3 and PM7-D5, while PM7-D4 shows a deviation from planarity (see the flat region from 50°–100° in the blue curve for PM7-D4 in the bottom panel of Figure 2). Overall, this is consistent with the average dihedral angles reported above for the optimized symmetric oligomers with full-length side chains.

We note that the θ_3_ dihedral angle of about 21° observed in the case of PM7-D3 oligomer is in fact a slightly higher energy local minimum on the torsional potential energy surface (see the minima approximately around 30° and 150° in the blue curve for PM7-D3 in the bottom panel of Figure 2). This suggests that factors such as S···O type interactions, commonly observed in thiophene-containing conjugated backbones,^31^ can play a significant role in minimizing the overall energy; such interactions could lead to one or more dihedrals adopting a less favorable local minimum in order to achieve a more favorable overall conformation. Since all the low-energy conformers in the PM7-D*x* series are separated by energy barriers of 3 kcal/mol or less per repeat unit, intrachain interactions are expected to facilitate the conversion to optimal conformations in the solid state and solution.

The DFT results derived for the excited-state properties of the PM7-D*x* oligomers are depicted in Figure S36 and reported in Table 1. The main absorption peaks arise from excitation from the ground state to the lowest excited state (S_0_ → S_1_) and correspond to transition energies of 2.51 eV (493 nm), 2.71 eV (458 nm), and 2.46 eV (504 nm) for the PM7-D3, PM7-D4, and PM7-D5 oligomers, respectively. The fact that the main absorption with lowest energy is calculated for PM7-D5 and the one with highest energy for PM7-D4, closely reflects the trends observed in the experimental UV–vis spectra of the polymer thin-films (discussed in the next section). Also, the calculated optical spectra of all polymers indicate the presence of a weak shoulder (see Figure S36) in the 350–450 nm region, which is related to several electronic transitions having a dominant intrachain charge-transfer character (see Figure S37). As reported in Table 1, the ionization potentials (IPs) of the oligomers vary in the order PM7-D5 (IP = 5.14 eV) < PM7-D3 (5.20 eV) < PM7-D4 (5.26 eV), which is consistent with the measured IPs for the polymers.

While it should be borne in mind that the conformations optimized for isolated PM7-D3, PM7-D4, and PM7-D5 oligomers can be affected by interchain interactions, our calculations point overall to high planarity for the backbones of both PM7-D3 and PM7-D5 and a twisted backbone for PM7-D4. These results are in good agreement with the experimental optical and electrochemical properties of these polymers.

### Photophysical and Electrochemical Properties

**Figure 3:**
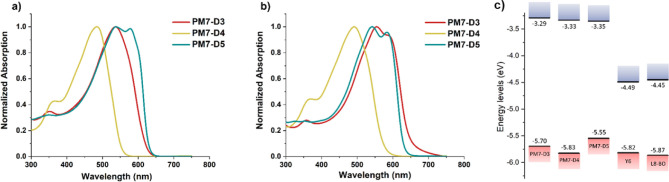
Normalized UV–vis absorption spectra of PM7-D*x* (*x* = 3, 4, and 5) in (a) solution (∼0.1 mg/mL in chlorobenzene) and (b) thin-films processed from chlorobenzene (10 mg/mL). (c) Energy diagram for donor phase PM7-D*x* (*x* = 3, 4, and 5) along with those for small-molecule acceptors Y6 and L8-BO. The state energies were calculated by assuming saturated calomel electrode (SCE) versus vacuum with respect to Fc/Fc^+^ to be 5.12 eV using the equation IP/EA = +e(*E*_ox_/_red_ + 5.12).

The solution UV–vis absorption spectra of PM7-D*x* (*x* = 3, 4, and 5) polymers in chlorobenzene shown in Figure 3a point to notable distinctions for the backbone conformation and aggregation properties among the quaterthiophene derivative polymers. The π–π* transition absorption in chlorobenzene features a ∼0.26 eV (55 nm) blue-shift of the maximum absorption wavelength for PM7-D4 (λ_max_ = 483 nm) compared to the other quaterthiophene diester polymers (λ_max_ = 538 nm for PM7-D3 and PM7-D5). This provides confirmation of a twisted backbone conformation for PM7-D4, which can negatively impact charge transport along the backbone, as shown before for other conjugated polymers.^32−35^ The π extension of the acceptor bridge to quaterthiophene in this series (compared to PM7 D1 and D2) is reflected in a solution λ_max_ red-shift for the D3 and D5 isomers supportive of a more planar backbone, as designed. While compared to the parent PM7 (fused-ring structure) with λ_max_ = 608 nm, a slight blue shift of solution λ_max_ for the D3 and D5 isomers is indicative of the structural flexibility obtained by incorporation of the opened-ring structure in the quaterthiophene unit.

The appearance of a low energy absorption band at 578 nm in the chlorobenzene solution for PM7-D5 is attributed to the aggregated organization of the polymer chains.^26,36−41^ The formation of such PM7-D5 aggregates in solution implies stronger interchain interactions potentially driven by a more planar structure.^42−46^ While the opened-ring structure in the quaterthiophene derivatives provides conformational flexibility, the side chains are distributed a further distance (2 esters for 4 thiophenes) from one another compared to PM7, PM7 D1 and PM7 D2, thus the polymers are distinct in terms of their side chain distributions, which will impact side-chain interdigitation and interchain interactions.^47−50^

Transitioning to the thin-film maximum absorption wavelengths shown in Figure 3b, we listed in Table S2 the solution-to-film λ_max_ shifts and thin-film absorption onsets (used to find optical bandgap) for the polymers. As evident in Figure S38, where the overlapped solution and thin-film absorptions are shown for ease of comparison, all polymers show red-shifted absorption upon thin-film formation. The magnitude of the red-shift for both λ_max_ and the absorption onset vary among the polymers, underscoring the different conformational changes each polymer undergoes during the transition from solution to solid state (spin-coated from chlorobenzene). PM7-D5 shows the smallest change in λ_max_ (4 nm) and unnoticeable onset red-shift. A low-energy sideband emerges for the thin-film absorption of PM7-D3, pointing to backbone planarization and formation of aggregated structures upon transitioning to the thin-film, while the thin-film absorption spectrum of PM7-D4 is blue-shifted and remains featureless. A structureless absorption band in conjugated polymers usually corresponds to the presence of multiple nonplanar conformers with minimal difference in their energies.^15,51^ Here, we observe three distinct conformation-aggregation behaviors. PM7-D3 is solvated without aggregation and shows ability to organize in the solid state; PM7-D4 is disordered both in solution and the solid state, while PM7-D5 shows aggregation properties in both solution and the solid state.

The thin-film redox properties of the PM7 quaterthiophene isomeric derivatives and the MAs were studied using differential pulse voltammetry (DPV) and are shown in Figure S39. Fresh films were used to obtain the separate oxidation and reduction DPV scans to properly capture the redox properties of the films by eliminating morphology disruptions due to initial electrochemical conditionings and electrolyte uptake. This cautious approach is used to give an estimate of the ionization energy (IE) and electron affinity (EA) in a material with a thin-film morphology resembling that used in the solar cells.^52,53^ Based on the DPV results, which were used to construct the energy diagrams in Figure 3c, the oxidation onset potential for PM7-D4 is highest (IP = 5.83 eV,  = 0.71 V versus Fc/Fc^+^) among the three polymers. This raised oxidation potential supports a twisted conformation for PM7-D4, which comes from the reduction in backbone conjugation at the highly nonplanar dihedral angles.^33,54^ The lowest IP, 5.55 eV ( = 0.43 V versus Fc/Fc^+^), is found for PM7-D5, while the PM7-D3 IP is measured to be 5.70 eV. The experimental IP trend of PM7-D5 < PM7-D3 < PM7-D4 is consistent with our DFT results on the oligomers. The 280 meV variation in ionization potential shows that varying the positions of the two ester functionalities within the quaterthiophene segment provides a useful tool to tune the state energies for better alignment (IP offset) with different (small-molecule or polymeric) acceptors as a means to optimize *V*_oc_ and *J*_sc_. The electrochemical gap, measured as the difference of onset of oxidation and reduction by DPV, is smallest for PM7-D5,  = 2.20 eV, as compared to PM7-D3,  = 2.41 eV, and PM7-D4,  = 2.50 eV; on the other hand, PM7-D3 has the smallest optical gap in the series, 1.92 eV. This is attributed to variations in the exciton binding energies defined as the difference between the electrochemical and optical gaps.^55−61^ These observations, correlating both our experimental and computational results, highlight the impact of the placement of the ester functionalities on the solution and solid-state backbone conformations and state energies, making these polymers a suitable series for conducting a systematic investigation of BHJ OPVs using MAs (in the present case, Y6 and L8-BO).

### OPV Device Studies

The OPV studies of these polymers were started by pairing them with Y6 (one of the most studied MAs, also known as BTP-4F) in an inverted device architecture (indium tin oxide glass/ITO/zinc oxide/PM7-D*x* (*x* = 3, 4, or 5):MA/MoOx/Ag) to elucidate the constitutional isomer effect of the donor phase polymer (see SI for device fabrication details). A summary of the device characteristics is included in Table 2 and a full screening of the results is listed in Table S3. We note that, as listed in Table S3, the addition of 5 vol % of a low vapor pressure cosolvent, chlorobenzene, improved the device performances considerably for all three isomeric polymers, with the performance of PM7-D4 remaining significantly lower than those of the other two isomeric polymers. (Table 2 lists the optimized conditions device characteristics additional 5 vol % of chlorobenzene) While PM7-D5 demonstrates an acceptable performance with an average power conversion efficiency of 8.9%, we observe a substantial difference in the performance of PM7-D4 despite its chemical similarity, 3.9%. This makes an ideal case for conducting more comprehensive comparative investigations of these systems.

**Table 2:** Device Screening Results Using Y6 as the Molecular Acceptor and Optimized Solvent Conditions^a^

Polymer blend	*J*_SC_ (mA cm^–2^)	*V*_OC_ (V)	FF (%)	Average PCE (best) (%)
PM7-D3:Y6	13.7 ± 0.63	0.83 ± 0.01	49.5 ± 1.9	5.90 ± 0.4 (6.5)
PM7-D4:Y6	9.72 ± 0.35	0.82 ± 0.01	46.8 ± 0.69	3.9 ± 0.2 (4.21)
PM7-D5:Y6	18.2 ± 0.39	0.76 ± 0.01	61.4 ± 1.8	8.9 ± 0.3 (9.30)

a Devices in this table are cast using an additional 5 vol % of chlorobenzene. The full comparison listed in Table S3 indicates improvement in PCE in all three polymers when a small amount of chlorobenzene is added.

In a second set of device studies, we achieved enhanced performance when using the more branched and soluble MA, L8-BO. Table 3 highlights enhanced fill factors and PCEs relative to Y6, with PM7-D3 attaining a 9.2% PCE and PM7-D5 attaining 11.4%. Additionally, it is worth noting that the ionization potential offset (ΔIP = IP(Acceptor) – IP(Donor)) (measured for the individual components) between PM7-D4 and Y6 is negligible (−0.01 eV), which could be another contributing factor to the underwhelming performance of PM7-D4. IP energy offset is generally discussed as an important factor in device performance,^62−66^ while examples of high-performance MA-based BHJ OPVs with near to zero IP energy offset are considered less common.^67,68^ Thus, L8-BO that has branched solubilizing side chains (butyl-octyl instead of *n*-undecane in Y6) and a higher IP (roughly speaking, deeper HOMO) that Y6 (as illustrated in Figure 3c) has been used in the morphological and device physics studies aimed at further elucidating the differences in the PM7-D3, PM7-D4, and PM7-D5 donor polymer series.

**Table 3:** Device Results Using L8-BO as the Molecular Acceptor^a^

Polymer Blend	*J*_sc_ (mA/cm^2^)	*V*_OC_ (V)	FF	PCE (%)
PM7-D3:L8-BO	18.6 ± 0.1	0.87 ± 0.01	0.56 ± 0.01	9.2 ± 0.1
PM7-D4:L8-BO	4.70 ± 0.1	0.88 ± 0.02	0.50 ± 0.03	2.1 ± 0.2
PM7-D5:L8-BO	20.9 ± 0.8	0.85 ± 0.01	0.64 ± 0.01	11.4 ± 0.6

a See the Supporting Information for fabrication details.

### Morphological Studies

**Figure 4:**
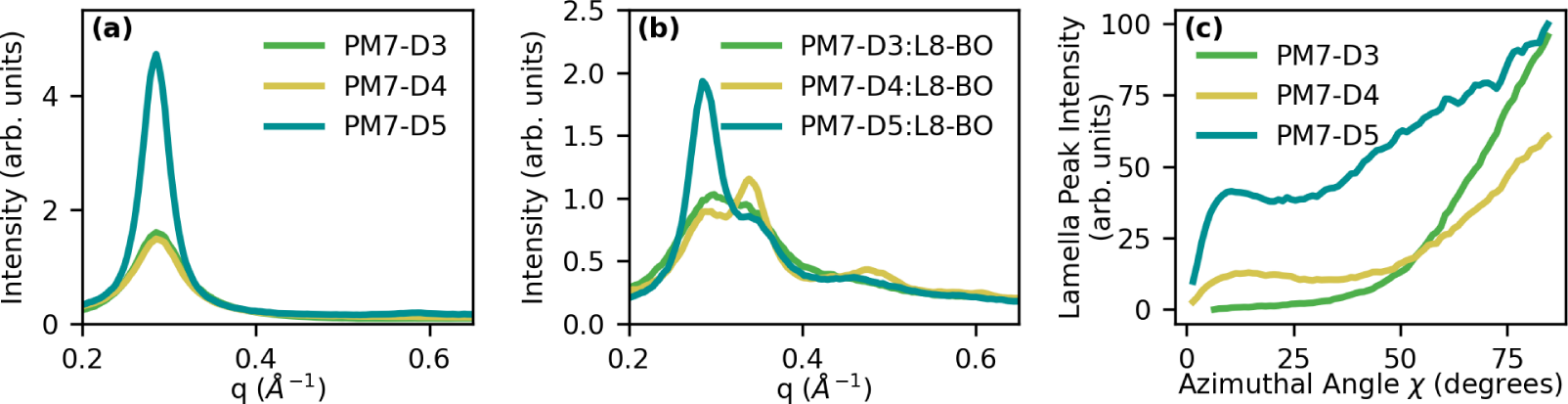
(a) Linecuts from neat polymer film GIWAXS. (b) Linecuts from bulk heterojunction film GIWAXS. (c) Pole figure showing the texturing and relative total fraction of lamella-ordered aggregates for each neat polymer film. Scattering intensities are normalized by film thickness and sin(χ) corrected as described in the literature.^69^

Grazing-incidence wide-angle X-ray scattering (GIWAXS) was used to understand how the opening of the benzodithiophene unit and the placement of the ester groups along the backbone alters the aggregation behavior of these polymers and whether the significant difference in performance among PM7-D3, PM7-D4, and PM7-D5 is rooted in morphological changes. GIWAXS measurements were taken on neat donor polymer films (Figure S40), a neat L8-BO acceptor film (Figure S41a), and BHJ films of donor:L8-BO (Figure S42). Films were prepared by spin coating on silicon substrates using the procedures replicating the device fabrication process. Quantitative descriptors of the morphologies formed by the different polymers were calculated by fitting a combination of pseudo-Voigt peaks to thickness normalized, sin(χ) corrected, and azimuthally integrated linecuts (Figures S41b, S43, and S44).^69,70^ The relevant fit parameters are collected in Table S4. Selected linecuts in Figure 4a,b show the low-*q* scattering of neat polymer and BHJ films, respectively. Interpretation of the linecuts relies on three metrics from the peak profiles: *d*-spacing from the peak center, relative aggregated fraction obtained by the integrated intensity, and crystalline coherence length (*L*_c_) indicated by the peak width. The crystalline coherence length is calculated using the Scherrer relationship, *L*_c_ = 2π*K*/Δ*q*, where a typical value of 0.9 was used for the shape parameter, *K*, and Δ*q* refers to the peak full width at half-maximum.^71−73^

First, we examine the morphology trends in neat donor polymer films, then draw connections to the more complex BHJ film scattering, and finally relate these morphology trends to characteristics of device performance. Linecuts of the neat donor polymer films, plotted in Figure 4a, show all polymers share a similar aggregate structure with lamella (100) and π–π (010) stacking. While the lamella peak position does not change significantly with polymer structure, the narrow and high intensity lamella peak in PM7-D5 indicates a larger proportion of well-ordered aggregates compared to PM7-D3 and PM7-D4. Specifically, the fitted lamella *L*_c_ of PM7-D5 at 145 Å is nearly double that of PM7-D4 at 82 Å, as given in Table S4. This difference in lamella *L*_c_ is greater than what has been shown to be caused by molecular weight differences in PM7-D5 and therefore is attributed to differences in polymer conformation.^18,74^ In contrast to the lamella (100) peak, the π–π stacking peak position changes with polymer structure, revealing a smaller *d*-spacing of 3.63 ± 0.001 Å in PM7-D5 films compared with 3.68 ± 0.009 and 3.76 ± 0.013 Å in PM7-D3 and PM7-D4, respectively. Finally, the sin (χ) corrected pole figure (Figure 4c) of the lamella scattering peak reveals that PM7-D5 exhibits more isotropic orientation of aggregates, while PM7-D3 and PM7-D4 are more strongly face-on oriented with respect to the substrate. Previous studies indicate that a lack of preferential orientation in films as seen for PM7-D5 can be caused by strong preaggregation in solution, as indicated by UV–vis for PM7-D5, which can kinetically lock-in isotropic orientations during film formation.^28,75,76^ It follows that the strong face-on orientation of the PM7-D3 film is reflective of its disaggregation in solution, and that aggregation occurs only in the film as indicated by the UV–vis measurements. Together these morphology trends in neat films strongly support the computational results of a well-planarized PM7-D5 that readily aggregates and of a twisted backbone for PM7-D4 hindering aggregation and preventing close π–π stacking, while PM7-D3 generally exhibits a morphology between the extremes of PM7-D4 and PM7-D5.

The morphology differences of the PM7-D*x* polymers seen in neat films are also present in the BHJ films of donor polymer paired with the L8-BO acceptor, apart from trends in the π–π peak since that peak in the BHJ films is a superposition of both donor and L8-BO stackings. The lack of changes in the donor lamella peak compared with the scattering from neat films indicates that the presence of the acceptor does not strongly influence the polymer aggregation. However, the linecuts, shown in Figure 4b, reveal that the extent of donor polymer aggregation strongly influences the aggregation behavior of the L8-BO acceptor. Comparing the ratios of the donor lamella peak intensity to acceptor (100) peak intensity in Table S4, we reveal that the strongly aggregating PM7-D5 polymer hinders L8-BO acceptor aggregation with a D:A peak intensity ratio of 2.3. On the other extreme, the hindered aggregation of PM7-D4 allows for stronger L8-BO aggregation, leading to a D:A peak intensity ratio of 1.0. This balancing of donor and acceptor aggregation in the BHJ films has been well documented already.^77−79^ These morphological results underscore the importance of balanced donor/acceptor aggregation and demonstrate that modifying the polymer backbone planarity is an effective strategy to control this aggregation balance.

### Device Physics

**Figure 5:**
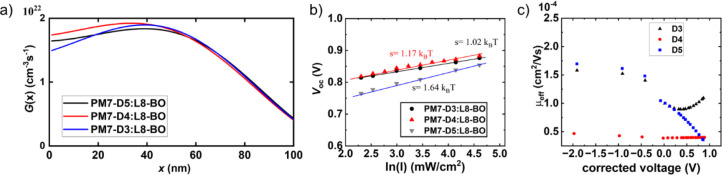
(a) Exciton generation rates, (b) *V*_oc_ dependence on light intensity, and (c) effective charge mobility in the three PM7-D*x*:L8-BO OPV devices.

To compare the device parameters for the three blends, we have fabricated inverted-structure OPV devices (glass/ITO/ZnO/PM7-D*x*:L8-BO/MoOx/Ag). The overall performance metrics follow the trends observed in the initial device testing using Y6 as the MA. In the first step, we evaluate the exciton photogeneration across the film thickness, via a previously reported method that relies on the reflectance and transmittance data along with the thicknesses of the BHJ films to obtain the optical constants, specifically the refractive index (*n*) and extinction coefficient (*k*).^80−82^ The exciton generation rate (*G*(*x*)) was then simulated with Optical Transfer Matrix Simulations.^80,82,83^ Interestingly, all three polymers demonstrate similar rates of exciton generation, see Figure 5a, indicating that the conformational differences observed among the donor polymers, such as the twisted backbone in PM7-D4, do not result in reduced charge generation rates in PM7-D4 and thus do not explain the observed differences in device performance.

Next, we carried out impedance spectroscopy measurements, followed by fitting of the recombination current, as described in the literature,^84,85^ to gain insight into charge recombination and extraction. The nongeminate recombination dynamics commonly play a critical role in OPV performance.^86,87^ Recombination can occur through band-to-band transitions via a so-called bimolecular recombination or via trap-assisted recombination. To quantify bimolecular recombination losses, we derived the Langevin prefactor ξ. The prefactor is low for all three systems, with values below 0.05 (ξ=0.05, ξ not detectable/within fitting error, and ξ = 0.01 for PM7-D3, -D4, and -D5, respectively). Thus, recombination via a trap-assisted mechanism should be considered. Trap states can arise through impurities or morphological defects,^87^ leading to trap-assisted recombination in the bulk or at the interfaces of the electrode/active layer. For the present systems, we find low densities of bulk traps (0.69 × 10^15^ cm^–3^) and moderate densities of surface traps (3.0 × 10^12^ cm^–2^) in the case of PM7-D3:L8-BO, values that are within the typically observed range of values for MA-based systems.^83,87^ These results suggest that bimolecular recombination can be the dominant recombination mechanism. In such a case, a slope close to unity is expected when measuring *V*_oc_ as a function of ln(I), with ln(I) being the natural logarithm of the light intensity.^88^ In fact, we observe a slope of 1.02 ± 0.01, see Figure 5b, confirming the conclusion of our impedance analysis for PM7-D3:L8-BO. The PM7-D4:L8-BO system shows significantly increased surface and bulk trap densities of 4.8 × 10^12^ cm^–2^ and 7.1 × 10^15^ cm^–3^. The bulk trap increase is more significant and, as expected, leads to an increase in the *V*_oc_ versus *I* slope to 1.17 ± 0.01. This increased trap-assisted recombination contribution can be considered one of the performance-limiting aspects of the PM7-D4:L8-BO device. However, as we will see when looking at PM7-D5:L8-BO, trap-assisted recombination seems not to be the primary performance-governing aspect in these devices as the surface trap density is lower (0.9 × 10^12^ cm^–2^) and the bulk trap density is comparable with those seen in the PM7-D4:L8-BO devices (6.9 × 10^15^ cm^–3^). Since PM7-D5:L8-BO devices show the highest performance, it can be concluded that the variations in performance do not primarily originate from the trap densities and the associated recombination lifetimes.

Thus, these results suggest that the origins of the poor performance must lie elsewhere. A comparison of the voltage-dependent mobility values, derived from the impedance spectroscopy data, reveals that indeed the D4 system suffers from extremely low mobility values (Figure 5c). It is likely that the low charge carrier mobility in PM7-D4 can be attributed to the twisted nature of the backbones and resulting short donor lamella *L*_c_. Additionally, overaggregation of the L8-BO acceptor may also contribute to the observed poor exciton splitting due to reduced D:A interfacial area. To summarize, the charge generation rates and recombination features are comparable between the isomeric donor polymers; however, the PM7-D4:L8-BO blend is suffering from large acceptor domains, reduced D:A interfacial area, and very low charge mobility leading to low Jsc and PCE.

**Figure 6:**
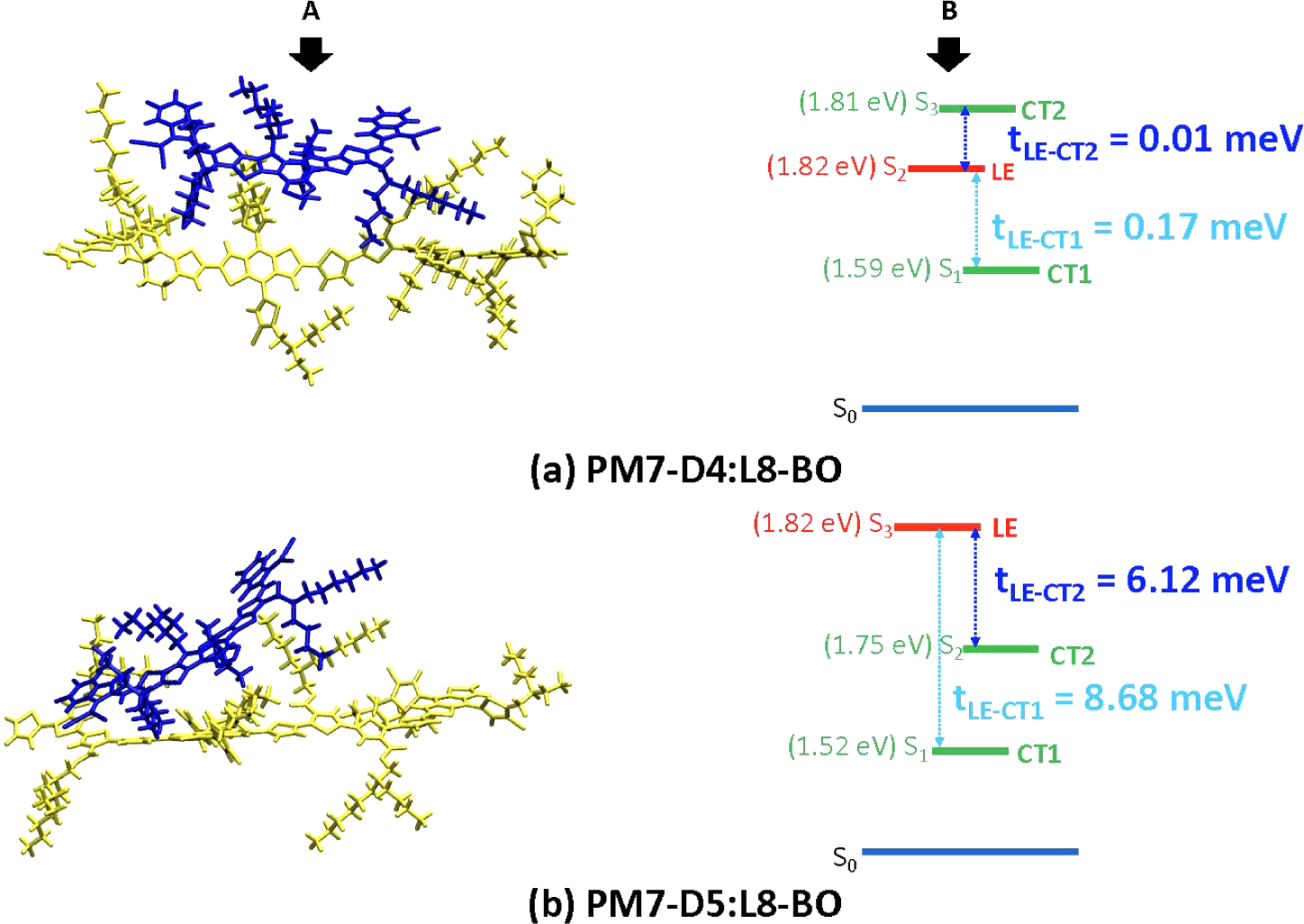
(A) Illustration of the optimized D:A complex configurations (where D and A are indicated by yellow and blue stick representations, respectively) and (B) energy diagram for the singlet electronic states (LE: local-exciton state, CT*n*: *n*th charge-transfer state, and S_0_: ground state) and electronic coupling values for complexes of (a) PM7-D4 and (b) PM7-D5 with the L8-BO acceptor, as calculated at the TD-DFT ωB97X-D/6-31G(d,p) level (with an implicit dielectric medium with ε = 3.5). The state energies are not drawn to proportion.

To gain additional insight into the low short-circuit current values, we carried out DFT calculations on model D:A complexes; we characterized the nature of their lowest local-exciton (LE) and donor–acceptor charge-transfer (CT) states as well as their electronic couplings, which are relevant to the exciton-dissociation rates (see the computational methodology section in the SI for more details on the construction of the model D:A complexes). The optimized configurations of the PM7-D4:L8-BO and PM7-D5:L8-BO complexes as well as their calculated state energies and related electronic couplings are shown in Figure 6 (and Figure S45 for PM7-D3:L8-BO). A major result in Figure 6 is that there is no significant electronic coupling between the LE and CT states in PM7-D4 compared to the other two isomers. In contrast, the LE-to-CT intramolecular reorganization energies in PM7-D4:L8-BO (0.34 eV) and PM7-D5:L8-BO (0.37 eV) are estimated to be nearly equal. As a result, the exciton dissociation rate constant derived in PM7-D5:L8-BO using a Marcus semiclassical model (see Supporting Information for details) is markedly larger than that in PM7-D4:L8-BO (2 × 10^12^ s^–1^ vs 6 × 10^8^ s^–1^). Since a smaller exciton dissociation rate constant is expected to produce a smaller current density, these computational results suggest an inferior device performance related to short-circuit current for the PM7-D4:L8-BO blends, which is in agreement with the experimental findings, see Table 3.

Finally, we note that our previous theoretical studies have shown that the microscopic parameters derived from DFT calculations based on donor:acceptor complexes (such as those discussed here) usually agree very well with the mean values derived from the distributions of these parameters computed by a more rigorous but much more computationally expensive approach that combines molecular dynamics simulations of the bulk morphology with DFT electronic-structure calculations.^89,90^

## Conclusions

In a family of PM7-D*x* polymers using quaterthiophene bridges, we have shown our ability to control the planarity of the conjugated polymer backbone through a combination of open-ring structures and variations in the placement of ester groups. We performed a systematic case study for three isomeric donor polymers blended with the molecular acceptors Y6 and L8-BO to investigate the effect of backbone planarity on OPV performance. The position of the ester groups within the quaterthiophene segments effectively modulates the backbone conformation (strongly twisted in PM7-D4), the solution aggregation behavior (no aggregation for PM7-D4, moderate aggregation for PM7-D3, and noticeable aggregation for PM7-D5) as well as the relevant electronic-state energies (the ionization potentials vary within a 280 meV window).

Devices based on PM7-D5 have power conversion efficiencies up to 11.4% when paired with L8-BO as the MA, whereas PM7-D4:L8-BO devices have a PCE of 2.1%. The differences between the performance of these isomeric donor polymers lay beyond the charge generation rates and recombination pathways. The twisted backbone in PM7-D4 leads to short donor lamella *L*_c_, overaggregation of the L8-BO acceptor, reduced D:A interfacial area, low charge carrier mobility, and insignificant electronic couplings between the local-exciton and charge-transfer states in the blend. Importantly, while backbone planarity usually corresponds to a better transport behavior (PM7-D4 that displays large backbone twists has a low charge mobility), the moderate rotational freedom provided by the open-ring structure in PM7-D3 and PM7-D5 keeps their ability to pack well in the solid state and have proper interactions with MAs (significant LE-CT electronic couplings).

## Experimental and Methods

### General Polymerization and Purification Procedures

Bisstannyl monomer (molecule 15 in Figure S2) (220 mg, 0.226 mmol, 1 equiv) and dibromo quaterthiophene monomer (207 mg, 0.226 mmol, 1 equiv of molecule 4, molecule 8, or molecule 14 respectively for PM7-D3, PM7-D4, and PM7-D5 as shown in Figure S2) were added to a freshly dried and cooled round-bottom flask containing a stir bar. Then the flask was taken to a glovebox where dipalladium-tris(dibenzylideneacetone)chloroform complex, Pd_2_(dba)_3_·CHCl_3_ as the catalyst (7.0 mg, 0.00678 mmol, 0.03 equiv), tris(*o*-tolyl)phosphine as the ligand, and 4 mL of toluene as the polymerization solvent were added to the flask. The reaction flask then was taken out of the glovebox and was immersed in an oil bath set at 105 °C and stirred for 16 h. At the end of this time, a small amount of palladium scavenger (diethylammonium diethyldithiocarbamate) was added, and the temperature was dropped to 90 °C. After stirring for 1 h at 90 °C, the solution was brought to room temperature and the crude polymer was precipitated into stirring cold methanol upon cooling to room temperature. The crude polymer was further purified by Soxhlet washing with methanol (24 h), Acetone (24 h), hexane (24 h), and the purified polymer was obtained from Soxhlet extraction using chloroform. The purified polymer then reprecipitated into cold methanol and collected by vacuum filtration on a nylon membrane with pore size of 45 μm.

### Device Fabrication Details (PM7-D*x*:Y6)

OPV devices were fabricated in an inverted device architecture (ITO)/ZnO/PM7-D*x*:Y6/MoO_3_/Ag. ITO substrates were purchased from Latech Scientific Supply Pte. Ltd., sheet resistance 10 Ω/sq. They were cleaned with dish soap and distilled water, followed by sonication in distilled water, acetone, isopropyl alcohol, subsequently for 10 min each. For the electron transport layer, a ZnO solution was made with 0.11 M Zn acetate dihydrate and 0.11 M ethanolamine combined in 2-methoxyethanol (Sigma-Aldrich). This solution was stirred overnight at room temperature, then filtered with a 0.45 μm PTFE syringe filter before use. The ZnO solution was deposited on the cleaned ITO substrates by spin-coating for 30 s at 4000 rpm in ambient atmosphere to get a layer thickness of ∼30 nm. After spin-coating, the ZnO layer was annealed in air at 150 °C for 15 min followed by slow cooling to room temperature and brought into an argon filled glovebox for active layer deposition. The active layers were prepared at a concentration of 20 mg/mL (D3 and D4) and 19 mg/mL (D5) in chloroform with or without 5% chlorobenzene (v/v), using a 1:1.2 donor:acceptor ratio. The active layers were spin-coated at 1500 (D3, D5) and 5000 rpm (D4) to yield active layers of 97 nm (D3), 93 (D4) and 74 nm (D5). After annealing of the films at 100 °C for 10 min, MoO_3_ (7 nm) and Ag (100 nm) electrodes were thermally evaporated at pressures <10^–6^ in a Ångstrom Engineering deposition chamber to obtain 6 complete solar cell devices per substrate with an electrode overlap area of 0.07 cm^2^. The *J*–*V* characteristics of all photovoltaic devices were evaluated under AM 1.5 G solar illumination (100 mW/cm^2^) using a Keithley SMU 2410 with a Newport Thermal Oriel 94021 solar simulator calibrated with a reference silicon solar cell.

### Device Fabrication Details (PM7-D*x*:l8-BO)

OPV devices were fabricated in an inverted device architecture (ITO)/ZnO/PM7-D*x*:L8-BO/MoO_3_/Ag. ITO substrates were purchased from South China Science & Technology Company Limited. They were cleaned with dish soap and distilled water, followed by sonication in distilled water, acetone, isopropyl alcohol, subsequently for 10 min each. The ZnO layers were prepared via spin-coating under ambient conditions at 4000 rpm. They were prepared from a solution of diethyl zinc in toluene (15%) mixed in a 1:2 ratio with THF. After spin-coating, the films were annealed annealing at 150 °C for 25 min. The substrates were transferred into a nitrogen-filled glovebox. The active layers were prepared at a concentration of 20 mg/mL (D3 and D4) and 19 mg/mL (D5) in chloroform with 5% chlorobenzene (v/v), using a 1:1.2 donor:acceptor ratio. The active layers were spin-coated at 1500 (D3, D5) and 5000 rpm (D4) to yield active layers of 97 nm (D3), 93 (D4) and 74 nm (D5). After annealing of the films at 100 °C for 10 min, MoO_3_ (7 nm) and Ag (100 nm) electrodes were thermally evaporated at pressures <10^–6^ in a Ångstrom Engineering deposition chamber using shadow masks with an area of 0.22 cm^2^.

### Morphology Studies

GIWAXS measurements were performed at the National Synchrotron Light Source II using beamline 11-BM. X-rays with an energy of 13.5 keV at an incident angle of 0.15 degrees were used to produce scattering profiles collected by a Pilatus 1 M detector (Dectris). The sample-to-detector distance of 258 mm was determined using a silver behenate standard. Scattering intensities of the linecuts were normalized by film thickness before fitting was performed in python using the lmfit package with a combination of linear background, exponential decay background, and pseudo-Voigt scattering peaks.

## Supporting Information Available

Syntheses methods, preparations, and characterizations of intermediate molecules, monomers, and polymers, including ^1^H NMR spectra and GPC chromatograms, DFT calculations, UV–vis spectroscopy, voltammetry, solar cell fabrication, and GIWAXS (PDF)
