# Supplementary material for: Manipulating Backbone Planarity of Ester Functionalized Conjugated Polymer Constitutional Isomer Derivatives Blended with Molecular Acceptors for Controlling Photovoltaic Properties
Source: Chem Mater. 2024 Nov 26;36(23):11656–68. doi: 10.1021/acs.chemmater.4c02751 (PMC11635973; doi:10.1021/acs.chemmater.4c02751)
Supplement: Supplementary file 1 — cm4c02751_si_001.pdf [file cm4c02751_si_001.pdf]

Supporting Information

for

Manipulating Backbone Planarity of Ester Functionalized Conjugated Polymer Constitutional Isomer Derivatives Blended with Molecular Acceptors for Controlling Photovoltaic Properties

Sina Sabury[1], Austin Jones[1], Nora Schopp[2], Sadisha Nanayakkara[3], Thomas P. Chaney[4], Veaceslav Coropceanu[3], Seth R. Marder[4,5,6], Michael F. Toney[4,5,6], Jean-Luc Brédas[3], Thuc-Quyen Nguyen[2], John R. Reynolds[1, *]

[1]School of Chemistry and Biochemistry, School of Materials Science and Engineering, Center for Organic Photonics and Electronics, Georgia Tech Polymer Network, Georgia Institute of Technology, Atlanta, Georgia 30332, United States.

[2]Center for Polymers and Organic Solids, Department of Chemistry and Biochemistry, University of California at Santa Barbara, Santa Barbara, CA 93106, USA.

[3]Department of Chemistry and Biochemistry, The University of Arizona, Tucson, AZ 85721-0041, United States

[4]Materials Science and Engineering Program, University of Colorado, Boulder, Colorado 80309, United States

[5]Department of Chemical and Biological Engineering, University of Colorado, Boulder, Colorado 80309, United States

[6]Renewable and Sustainable Energy Institute, University of Colorado Boulder, Boulder, Colorado 80303, United States

Table of Contents

1- OPV material examples 3

2- Polymer synthesis procedure 4

2-1 Synthesis of intermediate molecules and the quaterthiophene monomer for PM7-D3 5

2-2 Synthesis of intermediate molecules and the quaterthiophene monomer for PM7-D4 13

2-3 Synthesis of intermediate molecules and the quaterthiophene monomer for PM7-D5 22

3-Polymerization details and general polymer characterizations 34

4- DFT calculations 40

5- Solution processing optimization for device screening 46

6- Morphology studies 48

1- OPV material examples

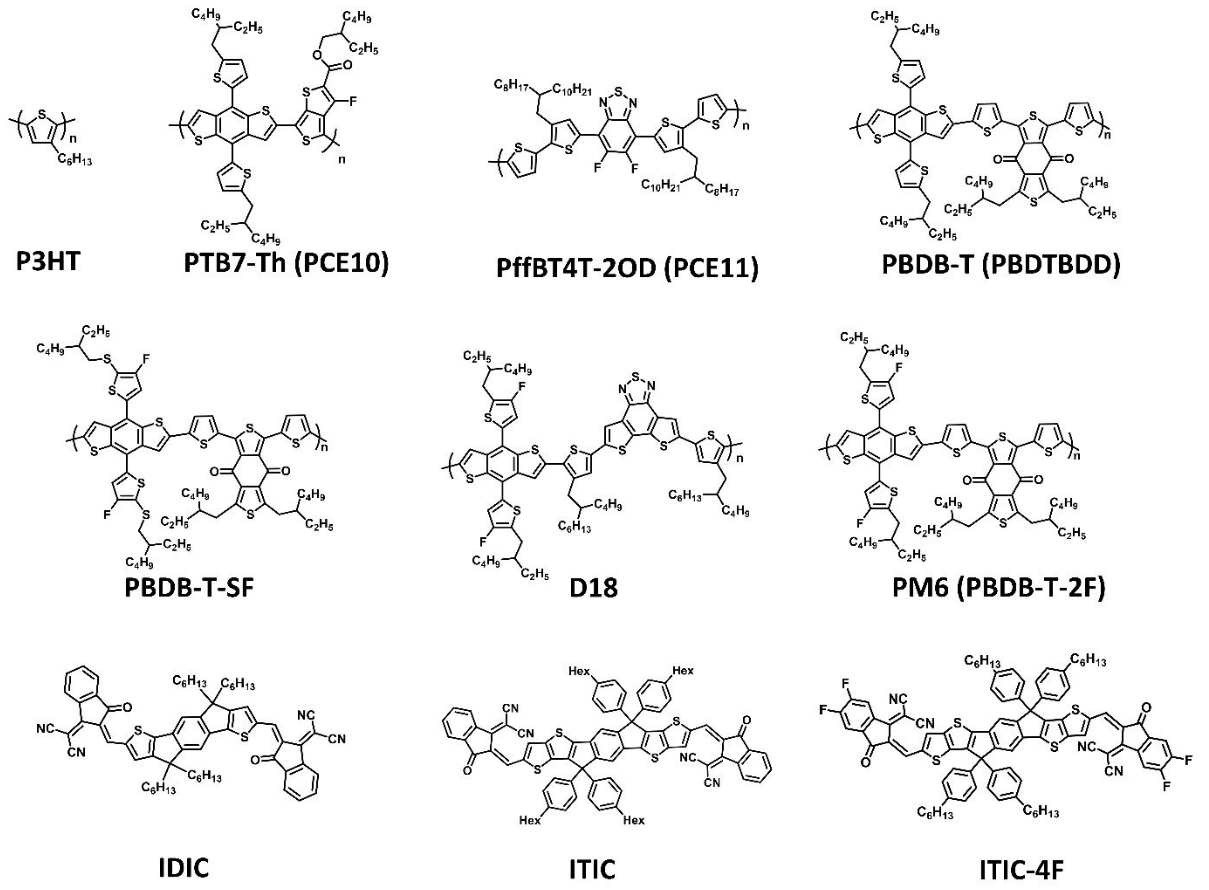

Figure S1. A few examples of donor polymers and molecular acceptors that have contributed to the current developments of organic solar cells.

2- Polymer synthesis procedure

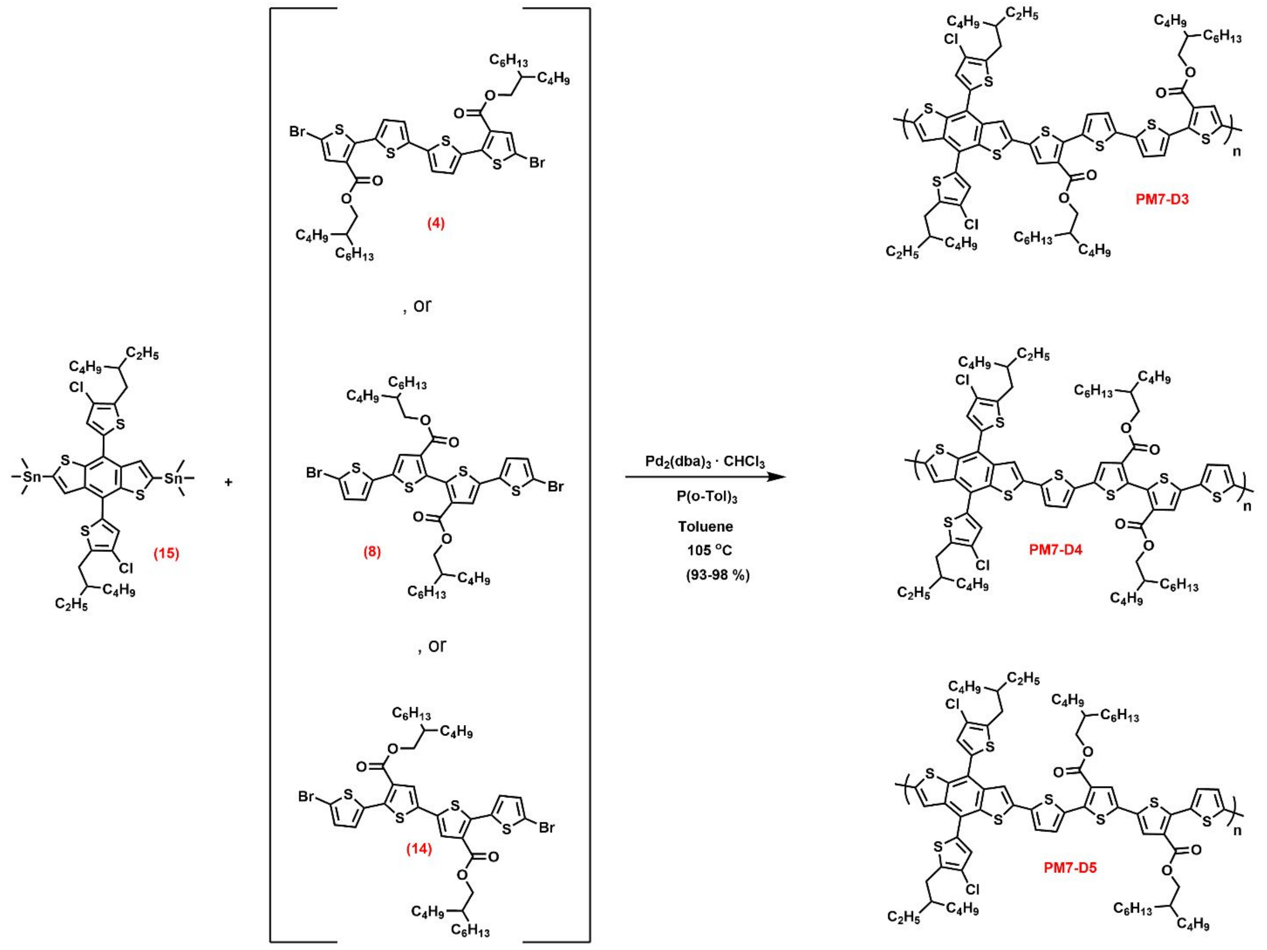

Figure S2. Synthesis of PM7-D3, PM7-D4, and PM7-D5 using three different constitutional diester quaterthiophene molecules.

2-1 Synthesis of intermediate molecules and the quaterthiophene monomer for PM7-D3

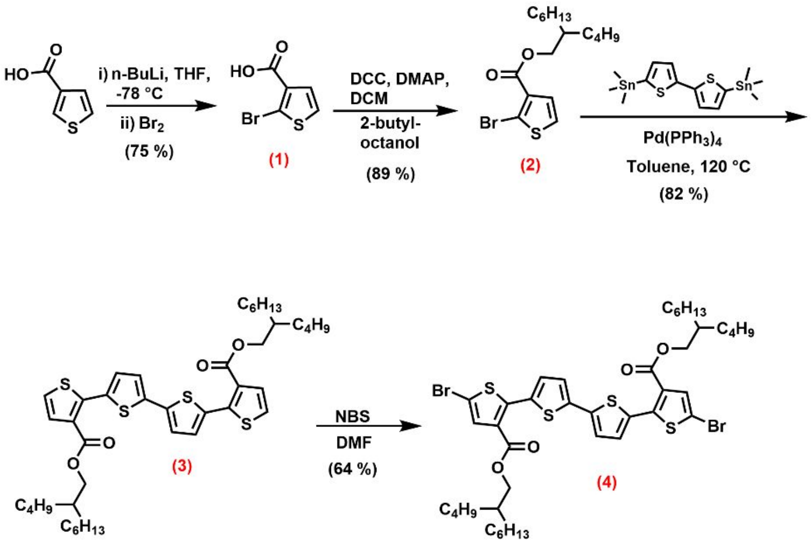

Figure S3. Synthetic pathway for molecule 4, the quaterthiophene monomer for PM7-D3.

Synthesis of 2-bromothiophene-3-carboxylic acid (1):

Similar to a reported procedure,[1] 3-thiophenecarboxylic acid (5 g, 39.0 mmol) was added to a dry 250 mL round bottom flask equipped with a stir bar. After transferring the flask to the glovebox, 100 mL of dry THF was added and placed under a constant flow of argon uppon removal from the glove box. The reaction vessel was chilled to -78 °C and 2 equivalents of 2.5 M n-butyllithium in hexanes (31.2 mL, 78 mmol) was added dropwise over 30 minutes. The reaction mixture was allowed to react for 3 hours at -78 °C followed by the dropwise addition of liquid bromine (2.1 mL, 40.7 mmol) at -78 °C. The mixture was then allowed to slowly warm up to room temperature while mixing over 16 hours. A small amount of HCl (2 mL of 1 M) was added to the reaction mixture turning the mixture into a clear solution. The solvent was removed under reduced pressure followed by the addition of ethyl acetate which was added to a separatory funnel and extracted with 1 M HCl twice. The organic layers were combined, dried over Na_2SO4 and filtered. The organic solvent was removed under reduced pressure to produce an impure white powder which

was recrystallized using a water/ethanol 4:1 mixture to produce white crystals (6.1 g, 75%).^{1}H NMR (500 MHz, C_2D_6OS): δ (ppm) 13.10 (s, 1H), 7.62 (d, J = 5.7 Hz, 1H), 7.32 (d, J = 5.8 Hz, 1H). NMR chemical shifts are consistent with a previous report.[1]

Synthesis of 2-butyloctyl 2-bromothiophene-3-carboxylate (2):

Similar to reported procedures,[2,3] 100 mL of dry dichloromethane (DCM) and 2-bromothiophene-3-carboxylic acid (1) (3 g, 14.5 mmol) were added to a dry 250 mL round bottom flask equipped with a stir bar. Then, 512 mg (4.2 mmol, 0.29 equiv.) of 4-dimethylaminopyridine (DMAP) was added followed by the addition of 1.25 equivalents of N,N'-dicyclohexylcarbodiimide (DCC) (3.75 g, 18.1 mmol). Then 1.1 equivalents of 2-butyl-1-octanol (2.97 g, 15.9 mmol) was added into the flask and the reaction mixture was left to stir overnight (16 hours) at room temperature. The solvent was removed under reduced pressure via rotary evaporation and the remaining contents were purified using silica gel column chromatography with 2:1 ratio of hexane:dichloromethane as mobile phase to afford a colorless oil (4.9 g, 89%). ^{1}H NMR (500 MHz, $CDCl_3$), δ(ppm): 7.36 (d, J = 5.8 Hz, 1H), 7.21 (d, J = 5.8 Hz, 1H), 4.20 (d, J = 5.5 Hz, 2H), 1.77-1.71 (m, 1H), 1.41-1.26 (m, 16H), 0.91-0.88 (m, 6H). $^{13}C\{^{1}H\}$ NMR (126 MHz, $CDCl_3$), δ (ppm):162.59, 131.87, 129.91, 126.18, 119.83, 68.09, 37.73, 32.21, 31.76, 31.44, 30.01, 29.35, 27.10, 23.38, 23.04, 14.49, 14.46.NMR spectra are consistent with a previous report.[3]

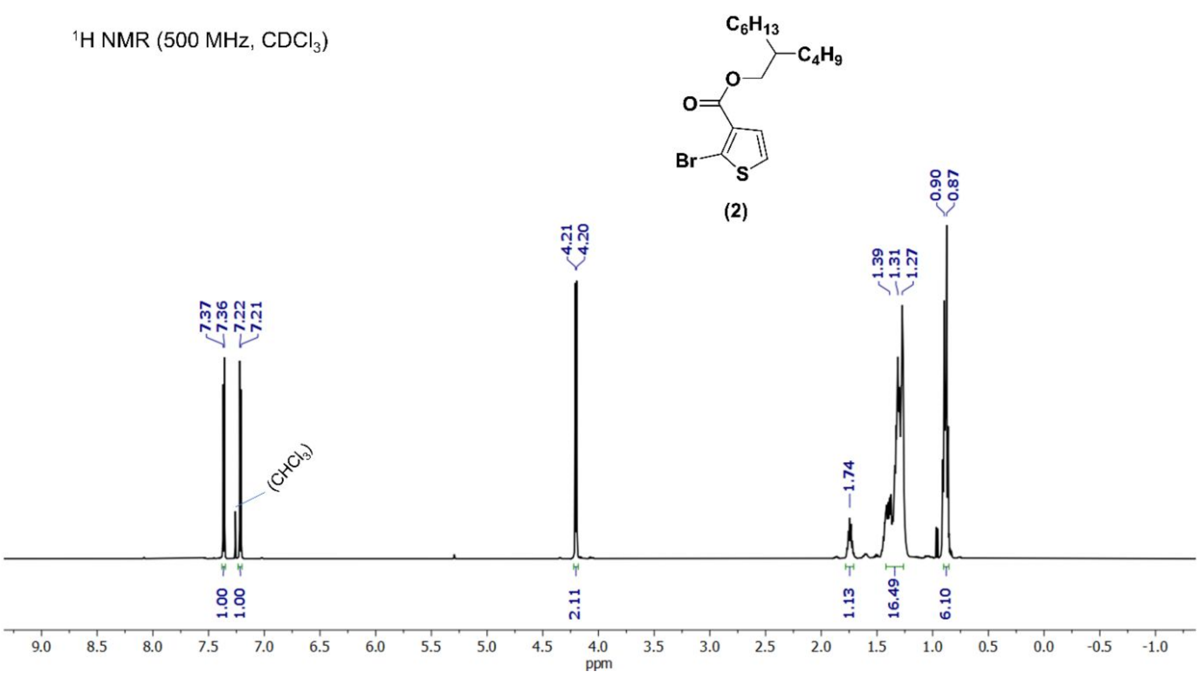

Figure S4. ^{1}H NMR of 2-butyloctyl 2-bromothiophene-3-carboxylate (2).

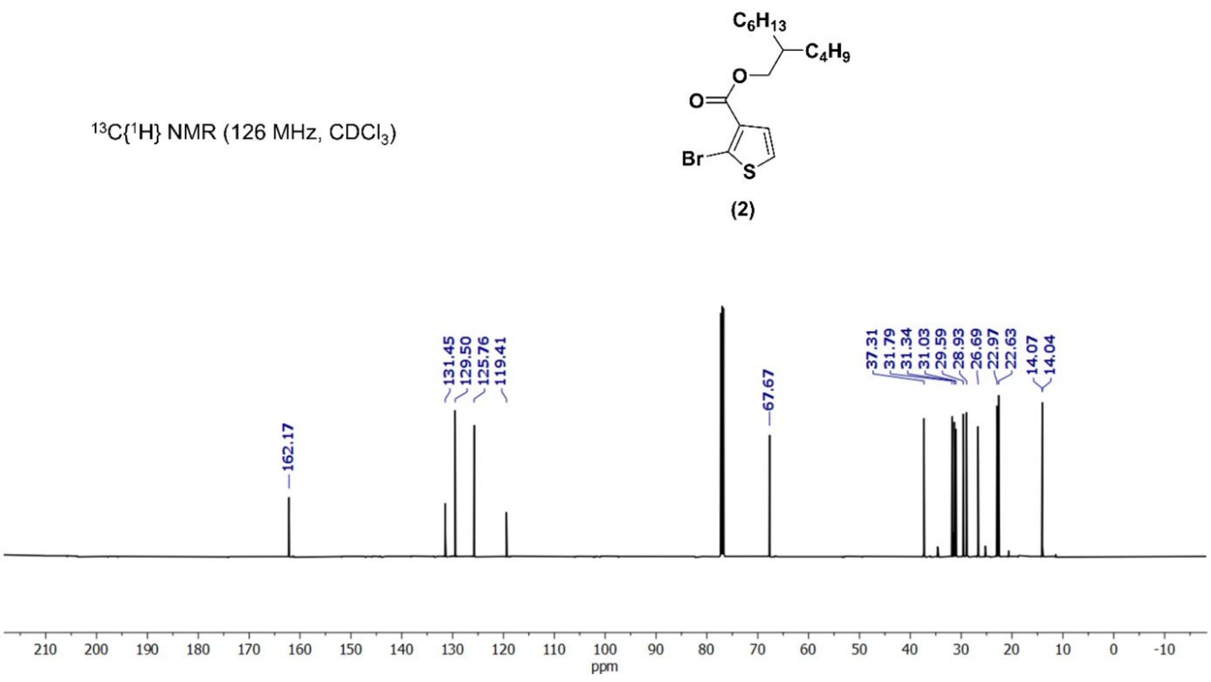

Figure S5. $^{13}C\{^1H\}$ NMR of 2-butyloctyl 2-bromothiophene-3-carboxylate (2).

Synthesis of bis(2-butyloctyl) [2,2':5',2'':5'',2'''-quaterthiophene]-3,3'''-dicarboxylate (3):

A dry 150 mL round bottom flask equipped with a stir bar, 5,5'-bis(trimethylstannyl)-2,2'-bithiophene (2 g, 4.07 mmol), and compound 2 (3.51 g, 9.35 mmol) was transferred into a glove box. Then, tetrakis(triphenylphosphine)palladium(0) (235 mg, 0.20 mmol) was added to the flask followed by 30 mL of dry toluene. The reaction flask was removed from the glove box and heated to 120 °C and stirred for 18 hours. The solvent was removed under reduced pressure and the remaining mixture was purified using silica gel chromatography using hexane:DCM (5:2) as the eluent to produce an orange oil (2.51 g, 82%). ^1H NMR (300 MHz, $CDCl_3$), δ(ppm): 7.48 (d, J = 4 Hz, 2H), 7.38 (d, J = 3 Hz, 2 H), 7.20 (d, J = 4 Hz, 2H), 7.16 (d, J = 3 Hz, 2H), 4.16 (d, J = 4 Hz, 4H), 1.73-1.66 (m, 2H), 1.34-1.22 (m, 32H), 0.90-0.83 (m, 12H); $^{13}C\{^1H\}$ NMR (126 MHz,

CDCl$_3$), δ (ppm): 163.32, 142.82, 139.04, 133.25, 130.62, 130.10, 128.03, 124.03, 123.87, 67.70, 37.42, 31.91, 31.41, 31.09, 29.72, 29.03, 26.79, 23.08, 22.74, 14.18, 14.15. HR-MS (APCI) m/z for $C_{42}H_{58}O_4S_4$ theoretical (M+H): 755.3290, found (M+H): 755.3279.

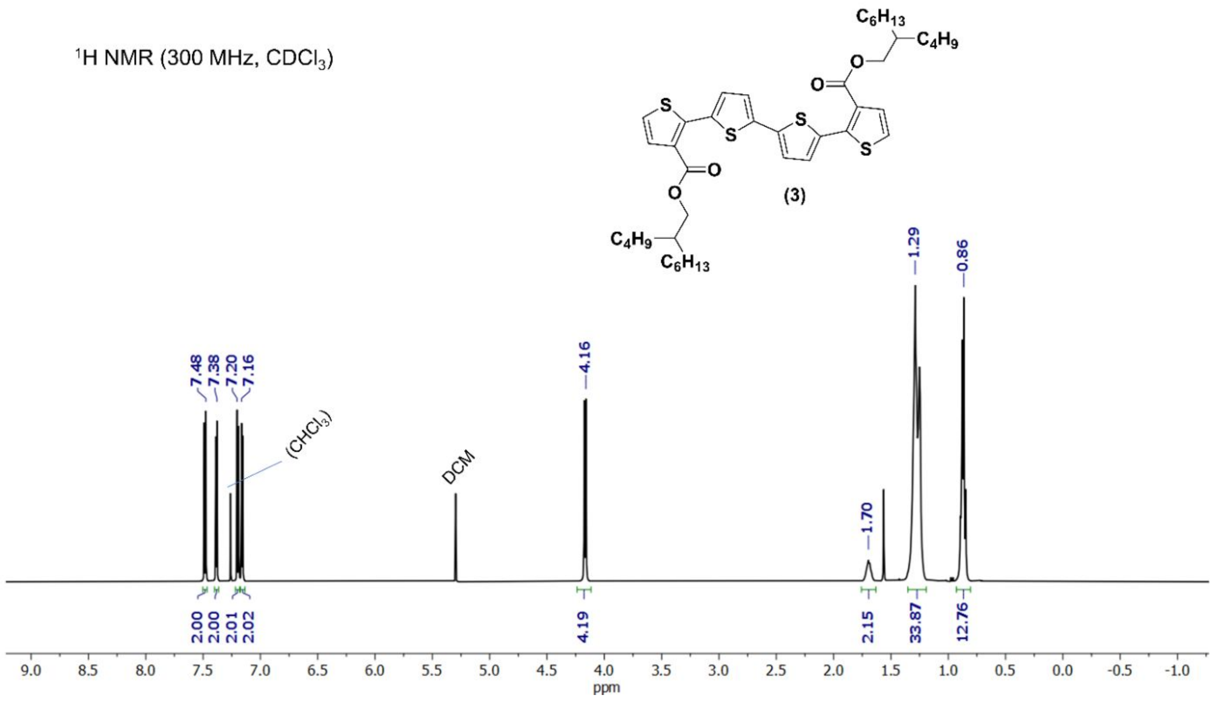

Figure S6. ^{1}H NMR of bis(2-butyloctyl) [2,2':5',2'':5'',2'''-quaterthiophene]-3,3'''-dicarboxylate (3).

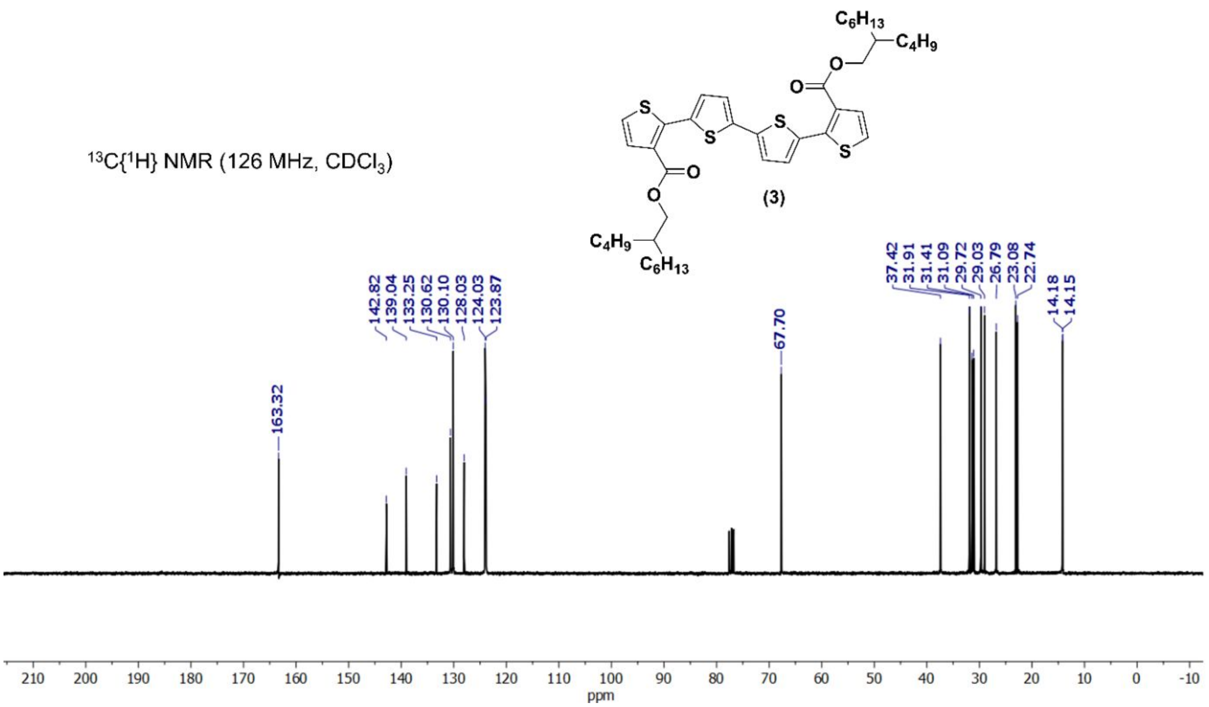

Figure S7. $^{13}C\{^1H\}$ NMR of bis(2-butyloctyl) [2,2':5',2'':5'',2'''-quaterthiophene]-3,3'''-dicarboxylate (3)

Synthesis of bis(2-butyloctyl) 5,5'''-dibromo-[2,2':5',2'':5'',2'''-quaterthiophene]-3,3'''-dicarboxylate (4):

To a 150 mL round bottom flask was added a stir bar, compound 3 (2 g, 2.65 mmol) and 70 mL of DMF. Then 2.7 equivalents of NBS (1.27 g, 7.13 mmol) were added to the solution in small portions at 0 °C. The reaction was sealed, protected from light, and stirred for 16 hours. Subsequently, the solvent was removed under reduced pressure followed by an extraction using DCM and water. The organic layer was dried over Na_2SO4, filtered, and concentrated under reduced pressure. The crude product was purified using silica gel chromatography using

hexane:DCM (4:1) as the eluent to produce an orange solid (1.55 g, 64%).^{1}H NMR (300 MHz, $CDCl_3$), δ(ppm): 7.42 (s, 2H), 7.33 (d, J = 3 Hz 2H), 7.14 (d, J = 3 Hz 2H), 4.16 (d, J = 4 Hz, 4H), 1.72-1.64 (m, 2H), 1.34-1.20 (m, 32H), 0.92-0.83 (m, 12H); $^{13}C\{^1H\}$ NMR (126 MHz, $CDCl_3$), δ (ppm):162.26, 144.14, 139.48, 132.89, 132.26, 130.55, 128.33, 124.31, 110.82, 68.11, 37.44, 31.97, 31.43, 31.12, 29.75, 29.09, 26.84, 23.13, 22.80, 14.25, 14.21. HR-MS (APCI) m/z for $C_{42}H_{56}Br_2O_4S_4$ theoretical (M+H): 911.1501, found (M+H): 911.1485.

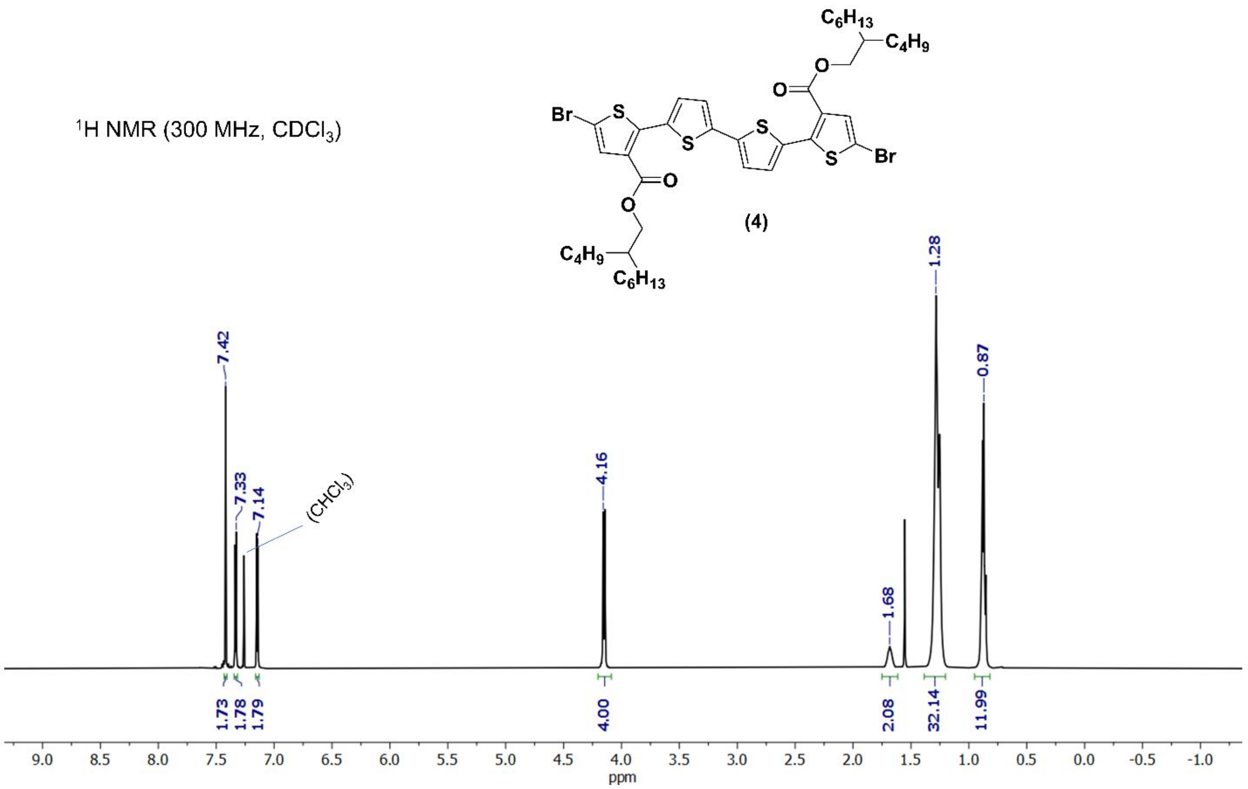

Figure S8. ^{1}H NMR of bis(2-butyloctyl) 5,5'''-dibromo-[2,2':5',2'':5'',2'''-quaterthiophene]-3,3'''-dicarboxylate (4).

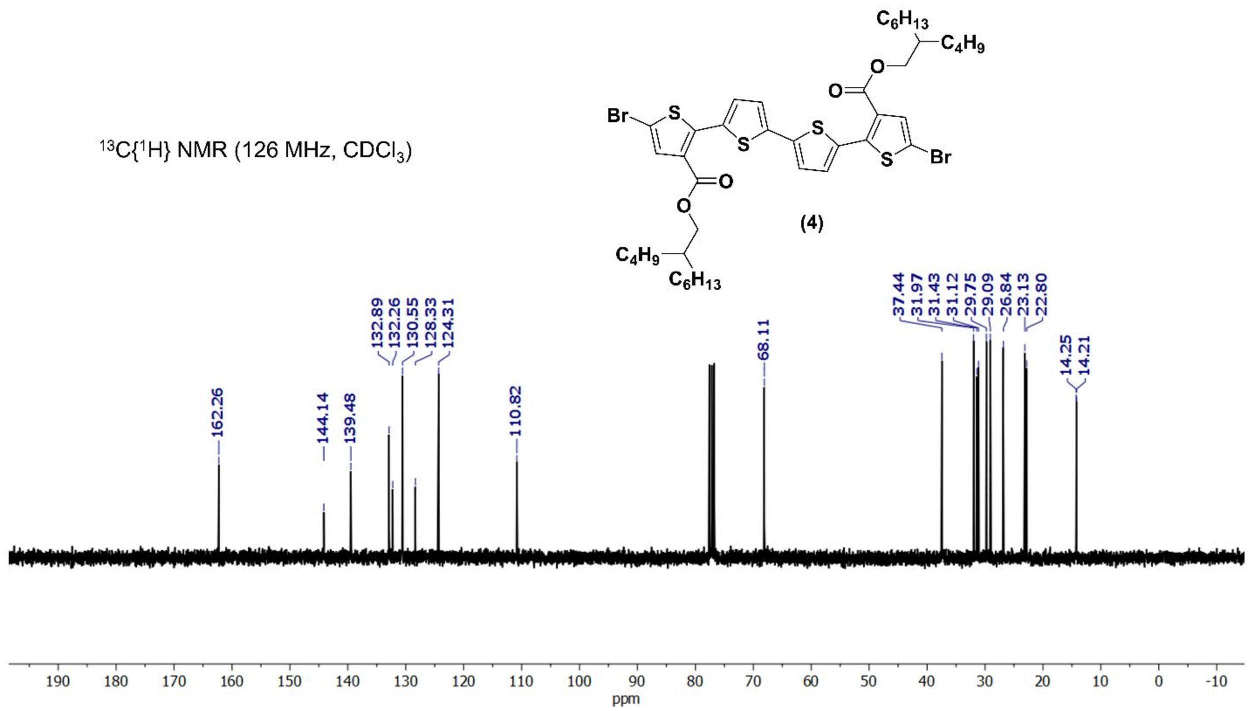

Figure S9. $^{13}C\{^1H\}$ NMR of bis(2-butyloctyl) 5,5'''-dibromo-[2,2':5',2'':5'',2'''-quaterthiophene]-3,3'''-dicarboxylate (4).

2-2 Synthesis of intermediate molecules and the quaterthiophene monomer for PM7-D4

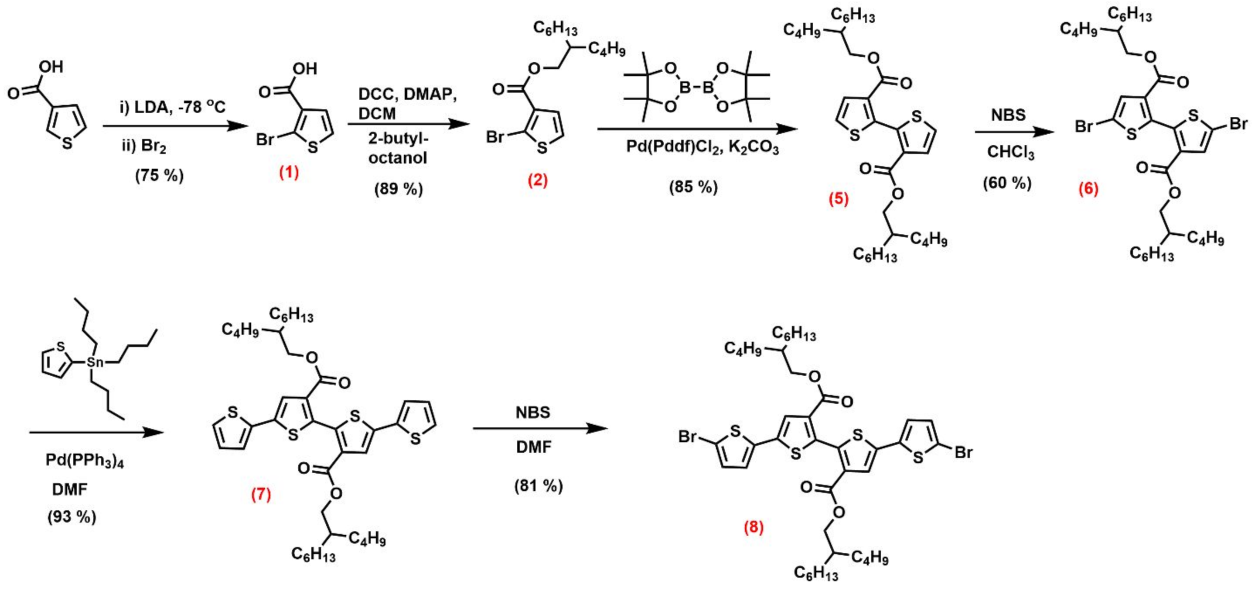

Figure S10. Synthetic pathway for molecule 8, the quaterthiophene monomer for PM7-D4.

Synthesis of bis(2-butyloctyl) [2,2'-bithiophene]-3,3'-dicarboxylate (5)

To a dry 250 mL round bottom flask were added 30 mL of dry dimethylformamide (DMF), 2-butyloctyl 5-bromothiophene-3-carboxylate (2) (1.88 g, 5.05 mmol), and a stir bar under positive argon pressure. Then, 558 mg (2.50 mmol, 0.5 equiv.) of bis(pinacolato)diboron was added followed by addition of 3 equivalents of potassium carbonate (K_2CO_3) (2.07 g, 15.15 mmol). Next, [1,1'-bis(diphenylphosphino)ferrocene]dichloropalladium(II), 1:1 complex with dichloromethane ($Pd(dppf)Cl_2 \cdot CH_2Cl_2$) (410 mg, 0.5 mmol, 0.1 equiv.) was added into the flask and the reaction mixture was left to stir overnight (16 hours) at 110 °C. The solvent was removed under reduced pressure and the remaining contents were purified using silica gel column chromatography with 3:2 ratio of hexane:dichloromethane as mobile phase to afford a light yellow oil (1.6 g, 85%). ^{1}H NMR (400 MHz, $CDCl_3$), δ (ppm): 7.54 (d, J = 5.4 Hz, 2H), 7.32 (d, J = 5.3 Hz, 2H), 4.00 (d, J = 5.6 Hz, 4H), 1.48-1.43 (m, 2H), 1.30-1.15 (m, 32H), 0.90-0.87 (m, 12H). $^{13}C\{^{1}H\}$ NMR (101

MHz, $CDCl_3$), δ (ppm): 162.87, 139.68, 132.15, 129.77, 125.67, 67.43, 37.24, 31.87, 31.04, 30.73, 29.60, 28.91, 26.68, 22.96, 22.68, 14.11, 14.09. NMR spectra are consistent with a previous report.[4]

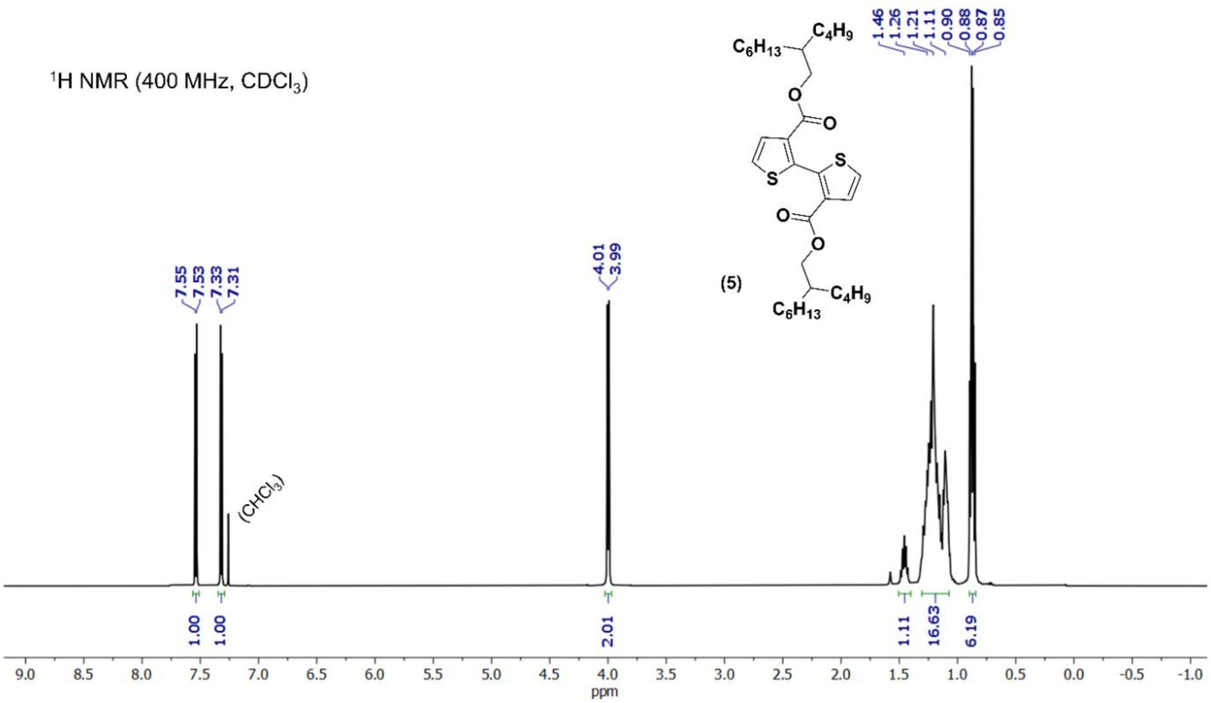

Figure S11. ^{1}H NMR of bis(2-butyloctyl) [2,2'-bithiophene]-3,3'-dicarboxylate (5).

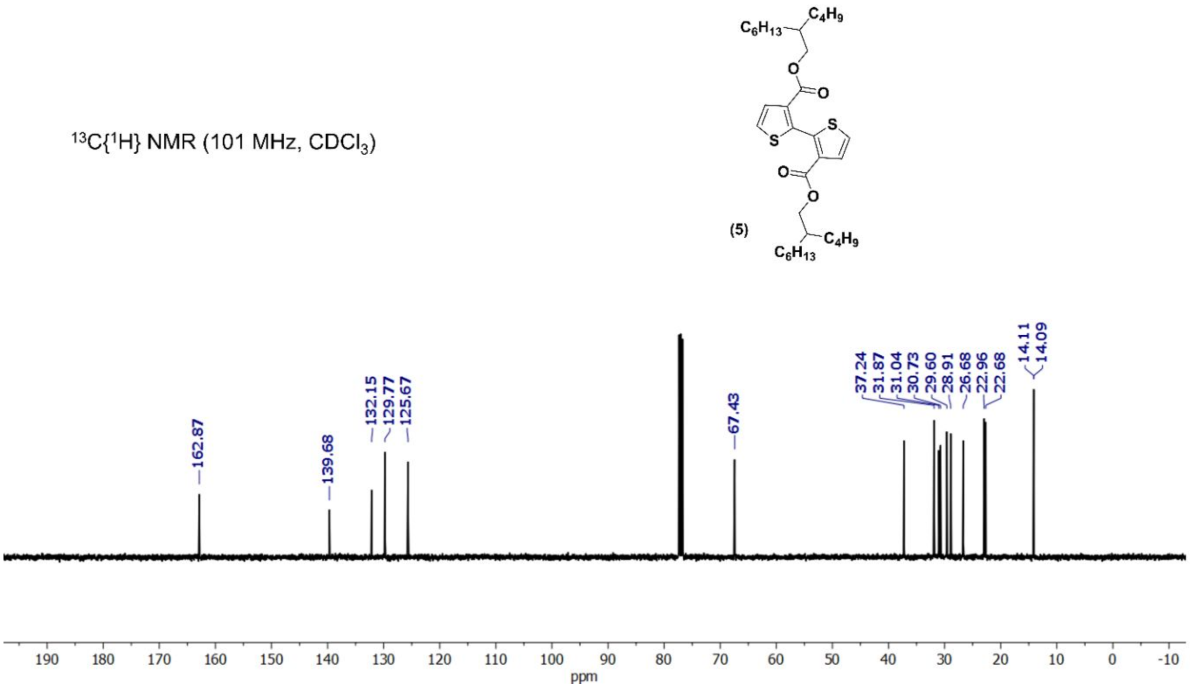

Figure S12. $^{13}C\{^1H\}$ NMR of bis(2-butyloctyl) [2,2'-bithiophene]-3,3'-dicarboxylate (5).

Synthesis of bis(2-butyloctyl) 5,5'-dibromo-[2,2'-bithiophene]-3,3'-dicarboxylate (6)

To a dry 250 mL round bottom flask were added 50 mL of chloroform, bis(2-butyloctyl) [2,2'-bithiophene]-3,3'-dicarboxylate (3) (1.10 g, 1.86 mmol), and a stir bar under positive argon pressure. Subsequently, 100 mL of chloroform and 20 mL of trifluoroacetic acid (TFA) were added to the reaction flask. N-Bromosuccinimide (NBS) (895 mg, 5.0 mmol, 2.7 equiv.) was added over 20 minutes to the flask at room temperature. The reaction mixture was left to stir overnight (16 hours) at room temperature. After this, the reaction was quenched by adding 50 mL of distilled water. The reaction mixture was extracted with 100 mL of DCM and the extract was washed with water followed by a brine solution. The organic layer was dried over sodium sulfate and the salt was filtered out. The solvent was removed under reduced pressure and the remaining contents were

purified using silica gel column chromatography with a 3:2 ratio of hexane:dichloromethane as mobile phase to afford a light yellow oil (838 mg, 70%). ^{1}H NMR (400 MHz, $CDCl_3$), δ (ppm): 7.47 (s, 2H), 4.02 (d, J = 5.4 Hz, 4H), 1.50-1.47 (m, 2H), 1.31-1.15 (m, 32H), 0.91-0.86 (m, 12H). $^{13}C\{^{1}H\}$ NMR (101 MHz, $CDCl_3$), δ (ppm): 161.49, 139.27, 132.87, 132.12, 113.37, 67.86, 37.26, 31.90, 31.08, 30.77, 29.63, 28.96, 26.73, 22.99, 22.70, 14.14, 14.13. NMR spectra are consistent with previous report.[4]

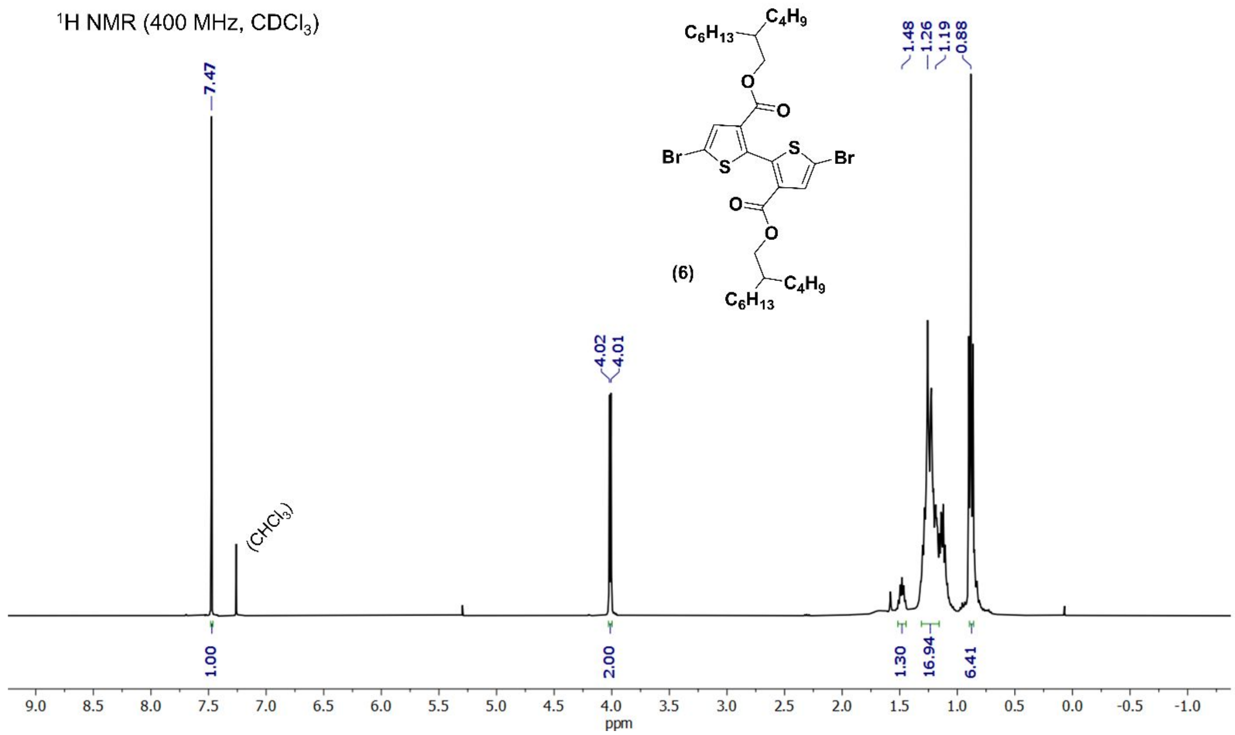

Figure S13. ^{1}H NMR of bis(2-butyloctyl) 5,5'-dibromo-[2,2'-bithiophene]-3,3'-dicarboxylate (6)

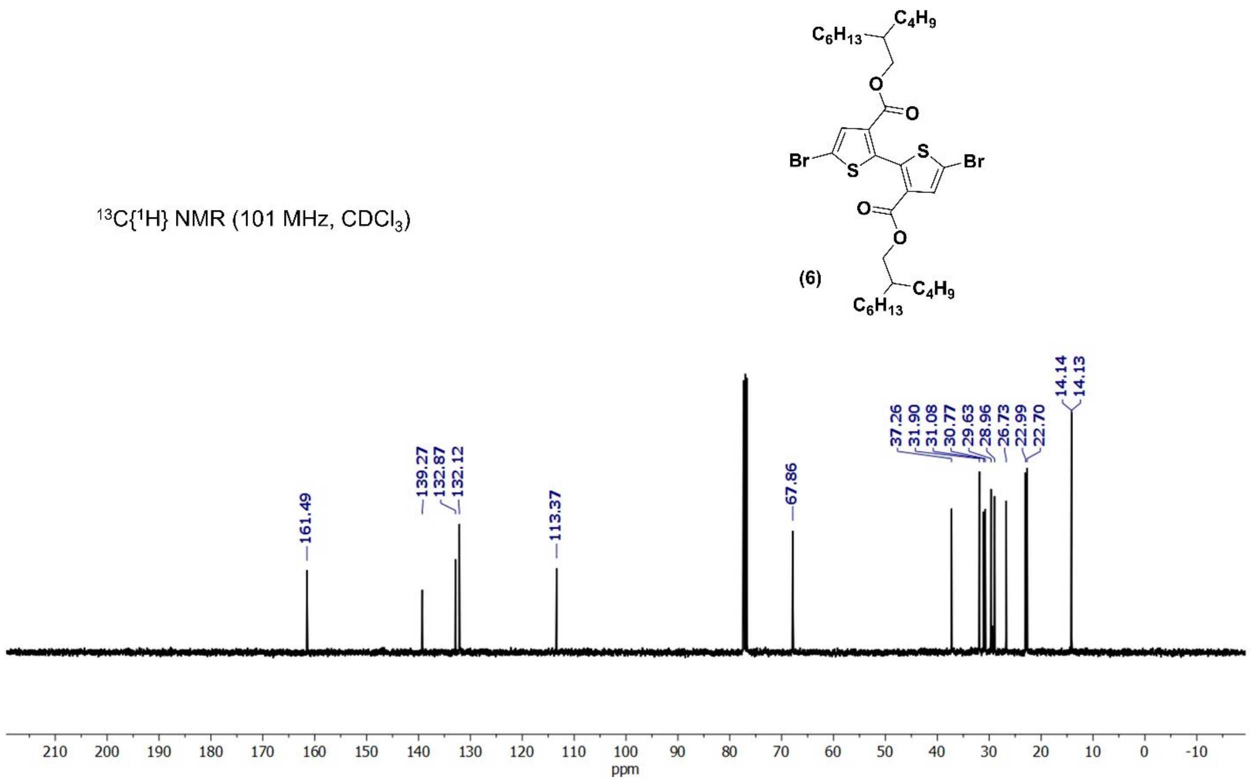

Figure S14. $^{13}C\{^1H\}$ NMR of bis(2-butyloctyl) 5,5'-dibromo-[2,2'-bithiophene]-3,3'-dicarboxylate (6).

Synthesis of bis(2-butyloctyl) [2,2':5',2'':5'',2'''-quaterthiophene]-3'',4'-dicarboxylate (7)

40 mL of DMF, bis(2-butyloctyl) 5,5'-dibromo-[2,2'-bithiophene]-3,3'-dicarboxylate (6) (547 mg, 0.73 mmol), and a stir bar were added to a dry 250 mL round bottom flask under positive argon pressure. Subsequently, 2-(tributylstannyl)thiophene (486.9 µL, 572 mg (d = 1.175 g/mL), 1.53 mmol, 2.1 equiv.) was added using a syringe to the flask at room temperature followed by addition of tetrakis(triphenylphosphine)palladium(0) ($Pd(PPh_3)_4$ (84 mg, 0.10 equiv.). The reaction mixture was left to stir overnight (16 hours) at 125 °C. After this, the reaction was quenched by adding 50 mL of distille water. The reaction mixture was extracted with 100 mL of DCM and the extract was washed with water and a brine solution. The organic layer was dried

over sodium sulfate and the salt was filtered out. The solvent was removed under reduced pressure and the remaining contents were purified using silica gel column chromatography with 2:1 ratio of hexane:dichloromethane as mobile phase to afford a light yellow oil (535 mg, 93%). ^{1}H NMR (500 MHz, $CDCl_3$), δ (ppm): 7.59 (s, 2H), 7.28 (dd, J = 5.1, 1.1 Hz, 2H), 7.20 (dd, J = 3.6, 1.2 Hz, 2H), 7.04 (dd, J = 5.1, 3.6 Hz, 2H), 4.03 (d, J = 5.5 Hz, 4H), 1.50-1.46 (m, 2H), 1.25-1.11 (m, 32H), 0.87-0.80 (m, 12H). $^{13}C\{^1H\}$ NMR (126 MHz, $CDCl_3$), δ (ppm): 162.68, 137.67, 137.14, 135.74, 132.76, 127.98, 125.61, 125.57, 124.62, 67.76, 37.27, 31.89, 31.61, 31.12, 30.79, 29.64, 28.97, 26.76, 22.97, 22.67, 14.12, 14.08. HR-MS (APCI,) m/z for $C_{42}H_{58}O_4S_4$ theoretical (M+H): 755.3290, found (M+H): 755.3282.

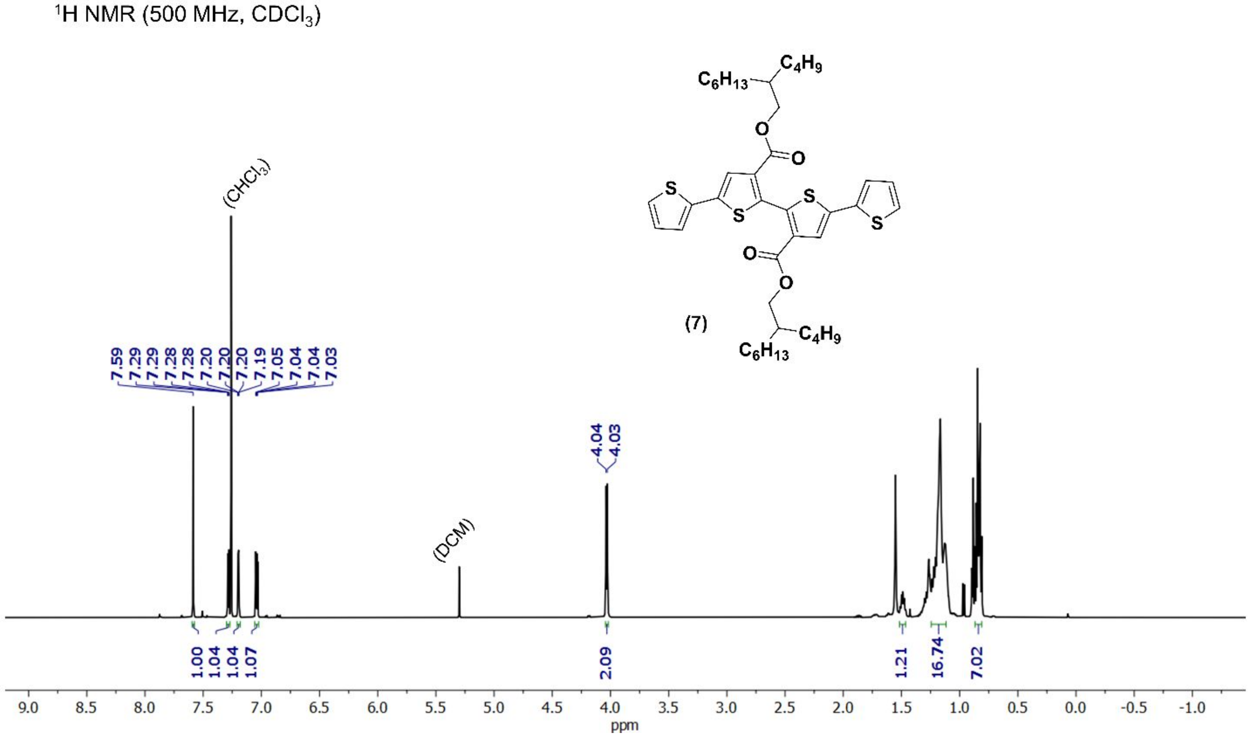

Figure S15. ^{1}H NMR of bis(2-butyloctyl) [2,2':5',2'':5'',2'''-quaterthiophene]-3'',4'-dicarboxylate (7).

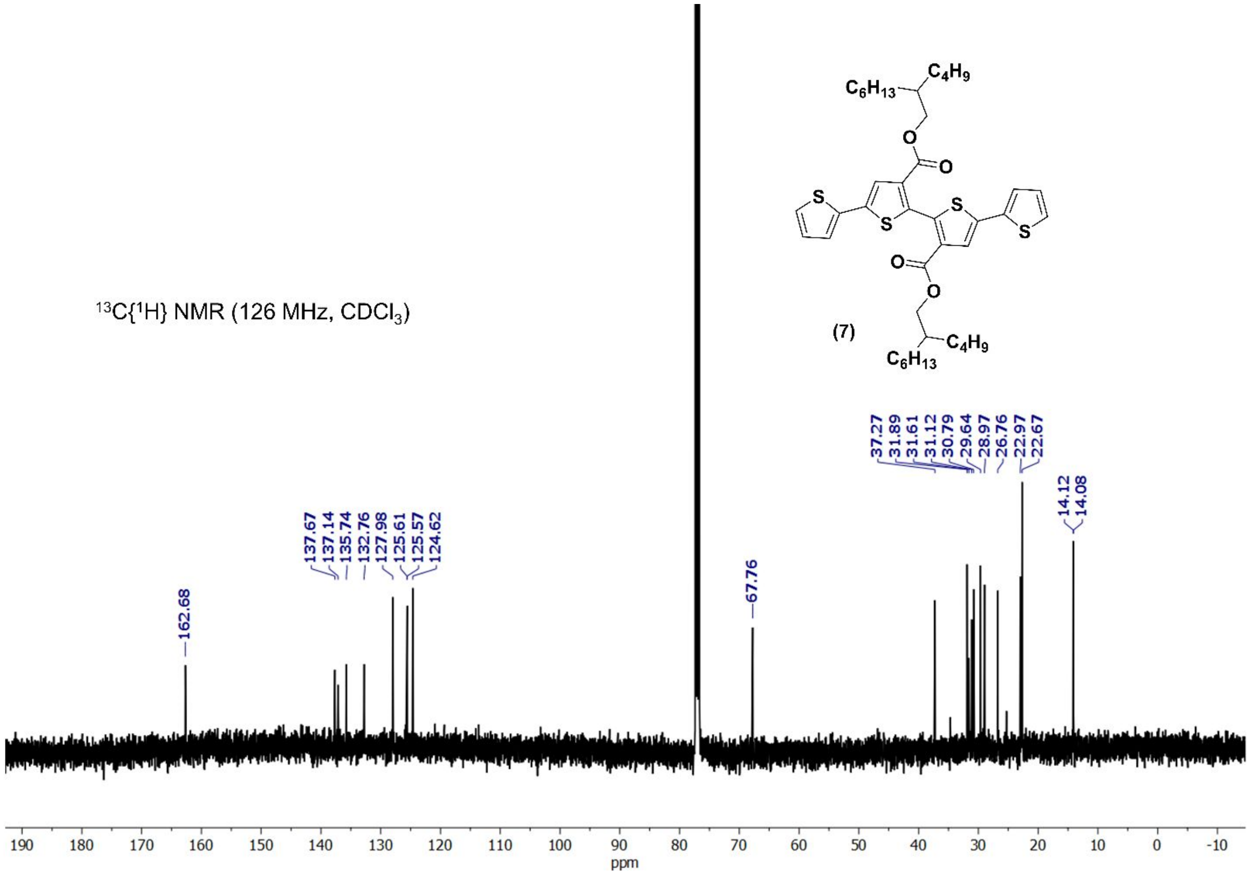

Figure S16. $^{13}C\{^1H\}$ NMR of bis(2-butyloctyl) [2,2':5',2":5",2'"-quaterthiophene]-3",4'-dicarboxylate (7).

Synthesis of bis(2-butyloctyl) 5,5'"-dibromo-[2,2':5',2":5",2'"-quaterthiophene]-3",4'-dicarboxylate (8)

To a dry 250 mL round bottom flask were added 50 mL of DMF, bis(2-butyloctyl) [2,2':5',2":5",2'"-quaterthiophene]-3",4'-dicarboxylate (7) (513 mg, 0.68 mmol), and a stir bar under positive argon pressure. Subsequently, NBS (254 mg, 1.43 mmol, 2.1 equiv.) was added over 20 minutes to the flask at room temperature. The reaction mixture was left to stir overnight (16 hours) at room temperature. After this time, the reaction was quenched by adding 50 mL of distilled water. The reaction mixture was extracted with 100 mL of DCM and the extract was washed with water and a brine solution. The organic layer was dried over sodium sulfate and the salts was filtered out. The solvent was removed under reduced pressure and the remaining contents were purified using

silica gel column chromatography with 2:1 ratio of hexane:dichloromethane as mobile phase to afford a yellow oil (504 mg, 81%). ^{1}H NMR (500 MHz, $CDCl_3$), δ (ppm): 7.51 (s, 2H), 7.00 (d, J = 3.9 Hz, 2H), 6.94 (d, J = 3.9 Hz, 2H), 4.03 (d, J = 5.5 Hz, 4H), 1.51-1.44 (m, 2H), 1.25-1.10 (m, 32H), 0.87-0.82 (m, 12H). ^{13}C{^{1}H} NMR (126 MHz, CDCl3) δ (ppm): 162.43, 137.21, 137.09, 136.69, 132.93, 130.83, 125.83, 124.77, 112.49, 67.85, 37.26, 31.92, 31.14, 30.78, 29.68, 28.96, 26.78, 22.98, 22.71, 14.14, 14.09. HR-MS (APCI,) m/z for $C_{42}H_{56}O_4S_4Br_2$ theoretical (M+H): 911.1501, found (M+H): 911.1502.

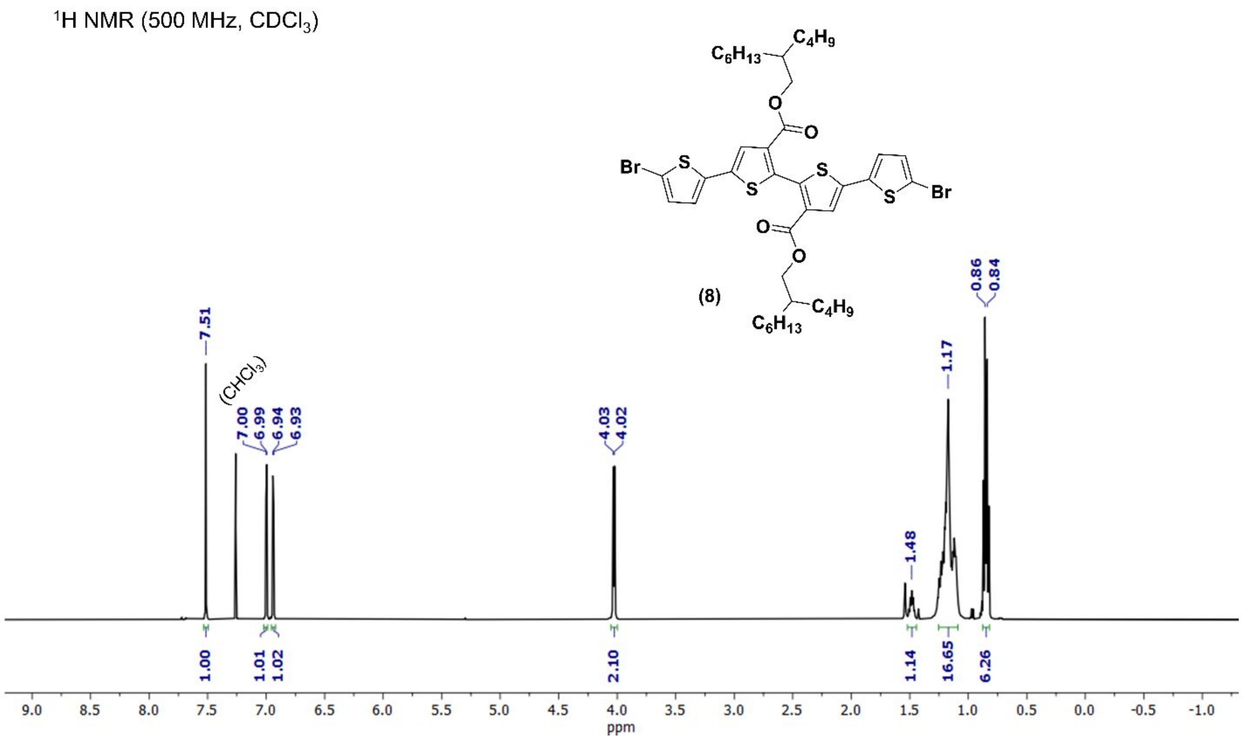

Figure S17. ^{1}H NMR of bis(2-butyloctyl) 5,5'''-dibromo-[2,2':5',2'':5'',2'''-quaterthiophene]-3'',4'-dicarboxylate (8).

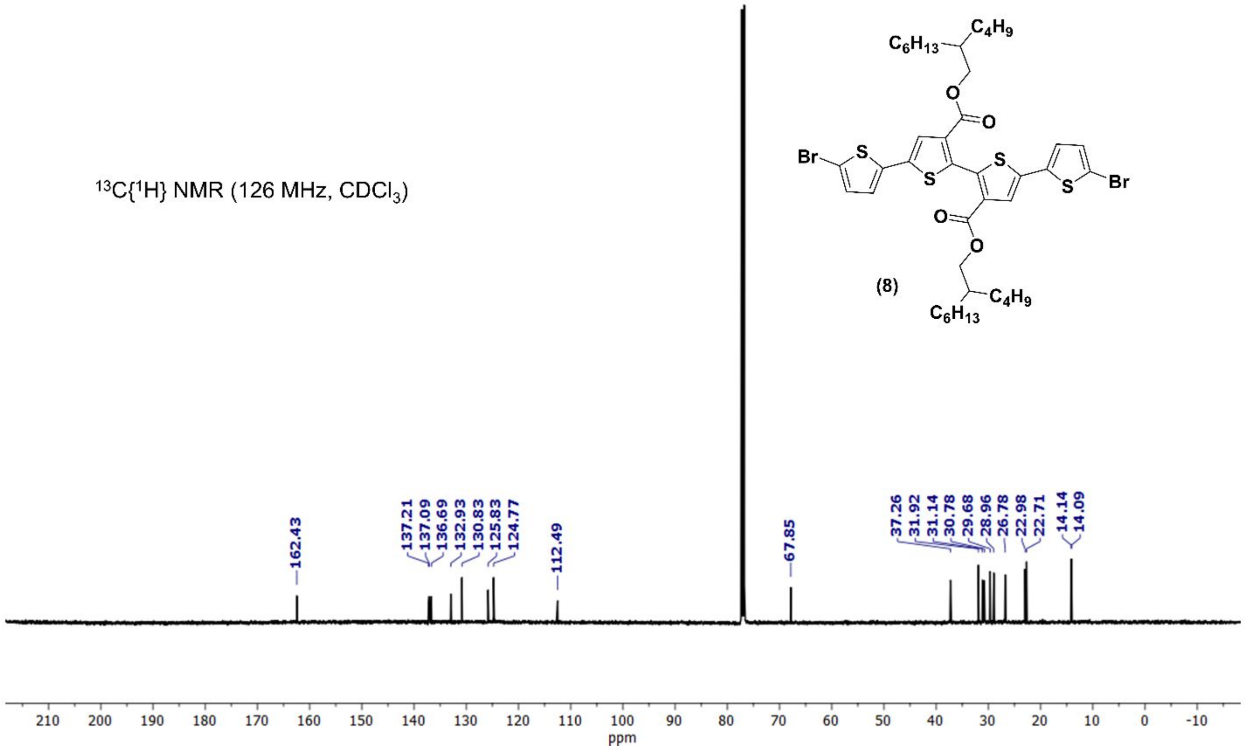

Figure S18. $^{13}C\{^1H\}$ NMR of bis(2-butyloctyl) 5,5'''-dibromo-[2,2':5',2'':5'',2'''-quaterthiophene]-3'',4'-dicarboxylate (8).

2-3 Synthesis of intermediate molecules and the quaterthiophene monomer for PM7-D5

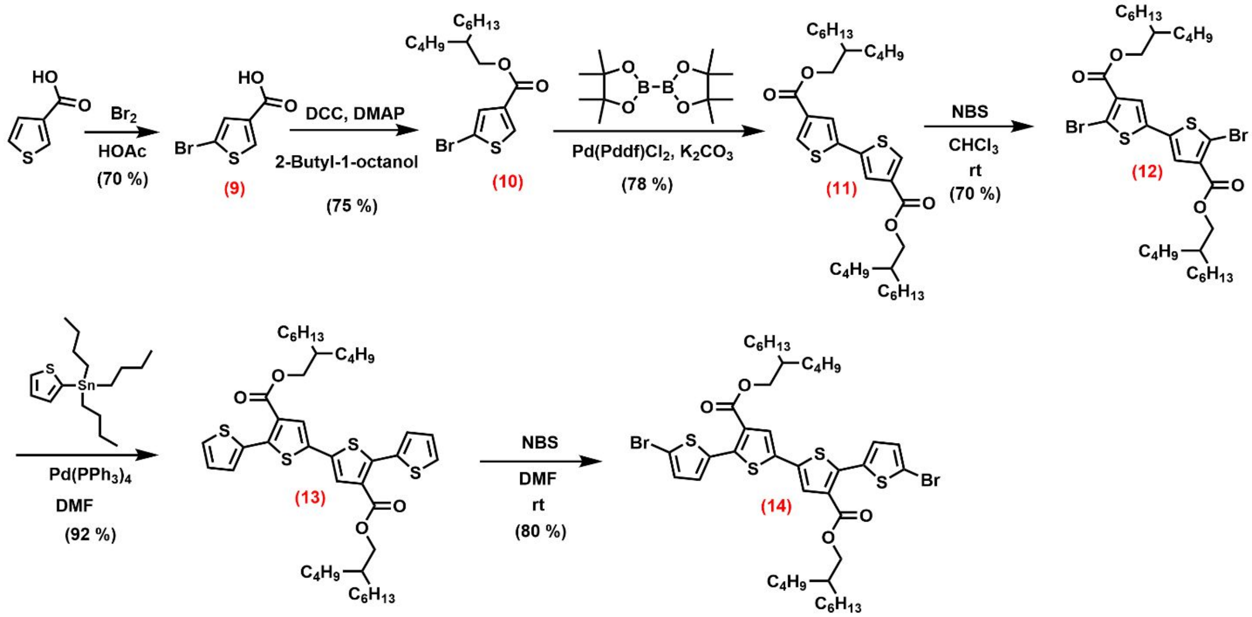

Figure S19. Synthetic pathway for molecule 14, the quaterthiophene monomer for PM7-D5

Synthesis of 5-bromothiophene-3-carboxylic acid (9):

Similar to a reported procedure,[5] 3-thiophenecarboxylic acid (6 g, 46.0 mmol) was added to a dry 250 mL round bottom flask equipped with a stir bar. Then, 50 mL of glacial acetic acid was added by syringe to the flask under positive argon pressure. Liquid bromine (2.2 mL, 42.7 mmol) was added dropwise over 20 minutes to the flask at room temperature. The reaction mixture was stirred for 2 hours at room temperature. At the end of this time, the reaction mixture was poured into cold water and the precipitate was isolated by vacuum filtration as a white powder. The desired product was obtained as white crystals after recrystallization from water and 10 v% ethanol. ^{1}H NMR (700 MHz, $CDCl_3$), δ (ppm): 8.12 (d, *J* = 1.5 Hz, 1H), 7.51 (d, *J* = 1.5 Hz, 1H). ^{13}C{^{1}H} NMR (176 MHz, $CDCl_3$), δ (ppm): 166.74, 135.84, 133.05, 130.38, 113.3. NMR spectra are consistent with a previous report.[5]

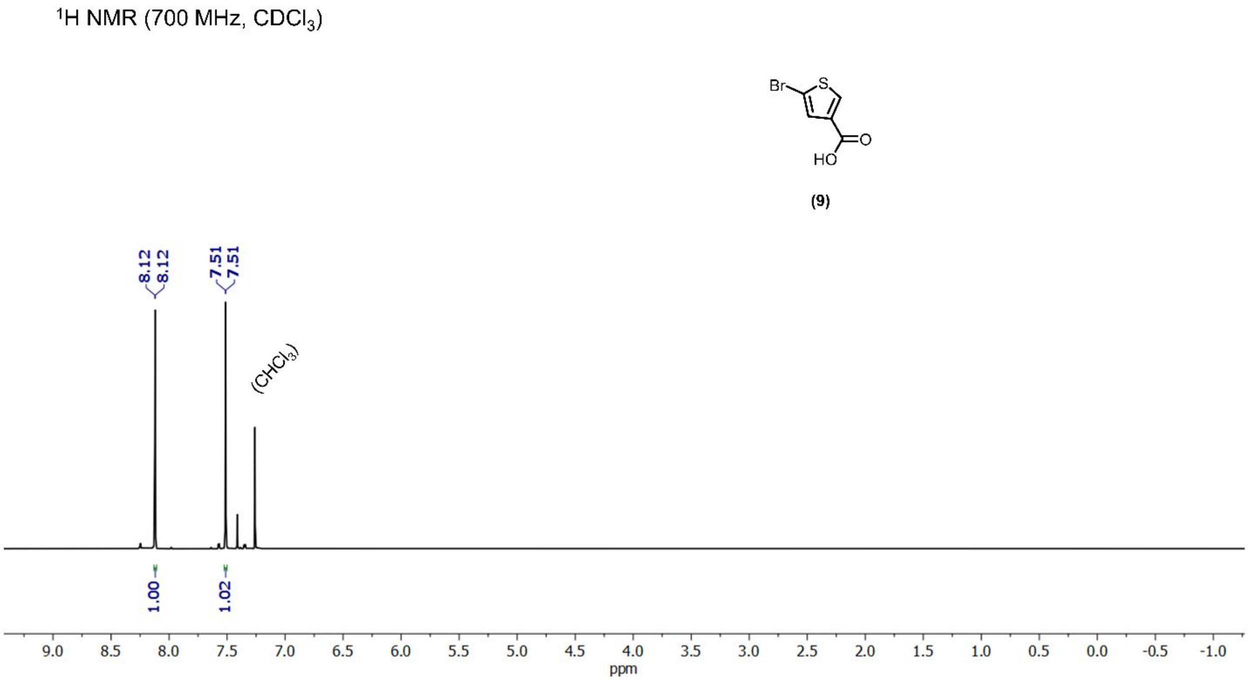

Figure S20. ^{1}H NMR of 5-bromothiophene-3-carboxylic acid (9).

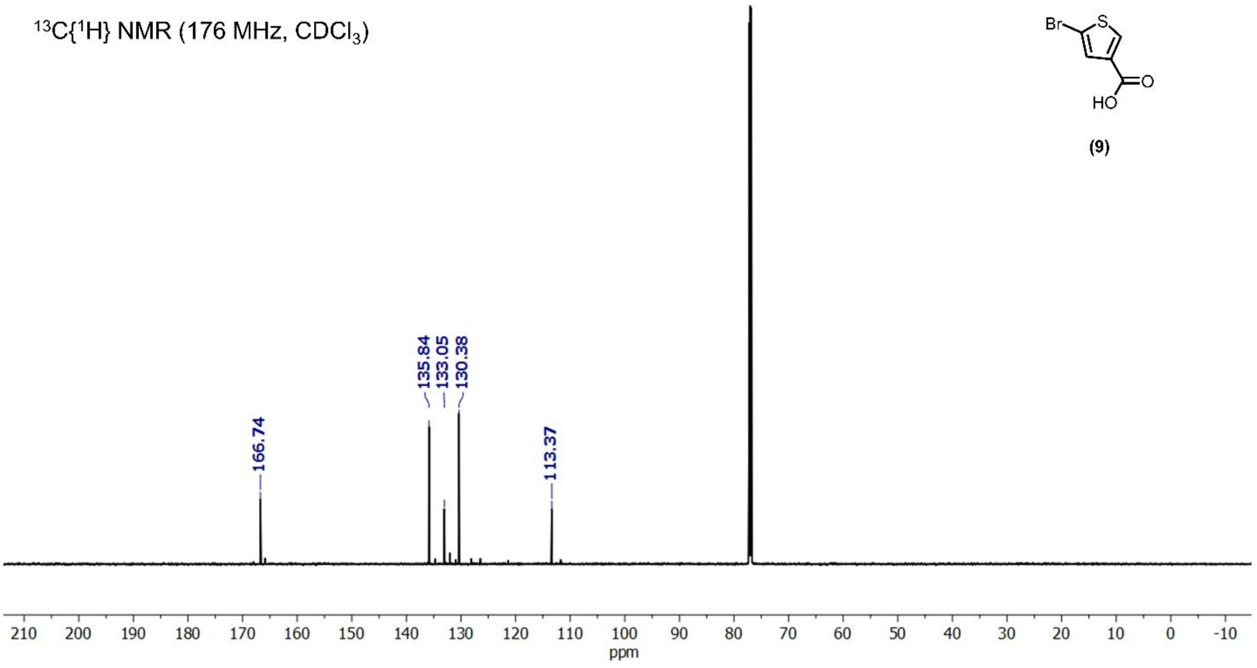

Figure S21. $^{13}C\{^{1}H\}$ NMR of 5-bromothiophene-3-carboxylic acid (9).

Synthesis of 2-butyloctyl 5-bromothiophene-3-carboxylate (10):

Similar to reported procedures,[1,6] 100 mL of dry dichloromethane (DCM) and 5-bromothiophene-3-carboxylic acid (1) (3 g, 14.5 mmol) were added to a pre-dried 250 mL round bottom flask equipped with a stir bar. Then, 512 mg (4.2 mmol, 0.29 equiv.) of 4-dimethylaminopyridine (DMAP) was added followed by the addition of 1.25 equivalents of N,N'-dicyclohexylcarbodiimide (DCC) (3.75 g, 18.1 mmol). Then 1.1 equivalents of 2-butyl-1-octanol (2.97 g, 15.9 mmol) was added into the flask and the reaction mixture was left to stir overnight (16 hours) at room temperature. The solvent was removed under reduced pressure via rotary

evaporation and the remaining contents were purified using silica gel column chromatography with 3.5:1.5 ratio of hexane:dichloromethane as mobile phase to afford a colorless oil (4.1 g, 75%). ^{1}H NMR (500 MHz, $CDCl_3$), δ(ppm): 7.96 (d, *J* = 1.5 Hz, 1H), 7.45 (d, *J* = 1.5 Hz, 1H), 4.16 (d, *J* = 5.7 Hz, 2H). 1.75-1.70 (m, 1H), 1.37-1.25 (m, 16 H), 0.90-0.86(m, 6H). ^{13}C{^{1}H} NMR (126 MHz, $CDCl_3$), δ (ppm): 161.73, 134.30, 133.61, 130.21, 112.84, 67.76, 37.39, 31.83, 31.40, 31.08, 29.62, 28.98, 26.73, 22.99, 22.67, 14.12, 14.07. NMR spectra are consistent with a previous report.[6]

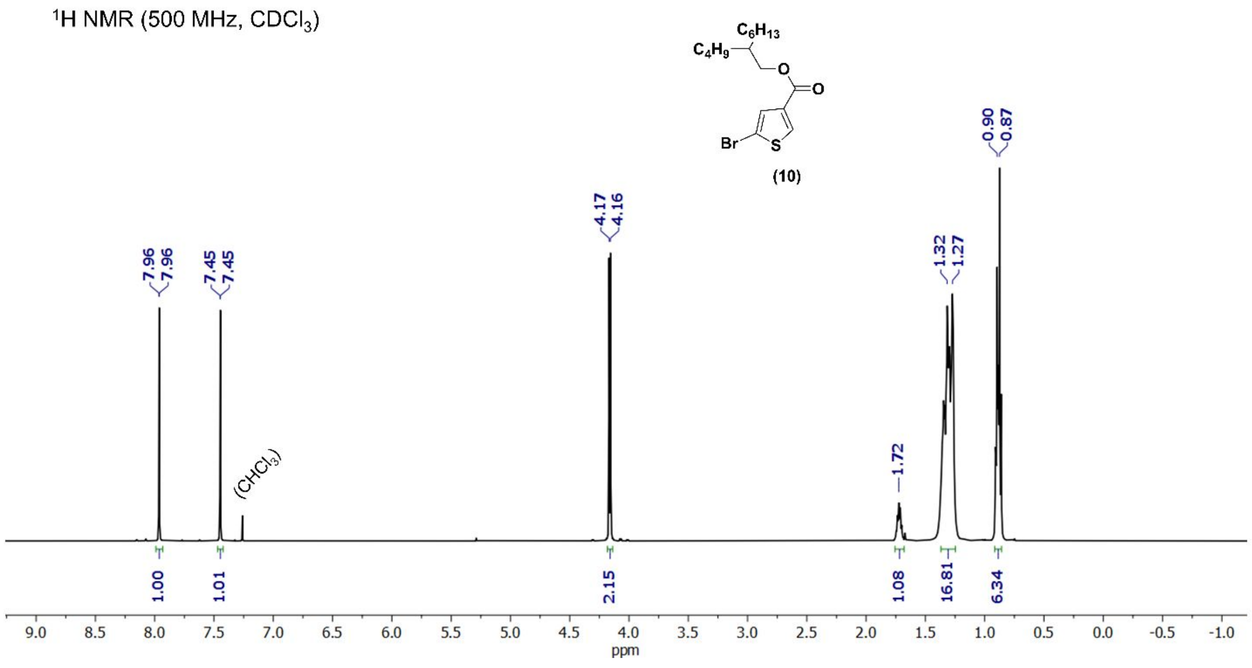

Figure S22. ^{1}H NMR of 2-butyloctyl 5-bromothiophene-3-carboxylate (10).

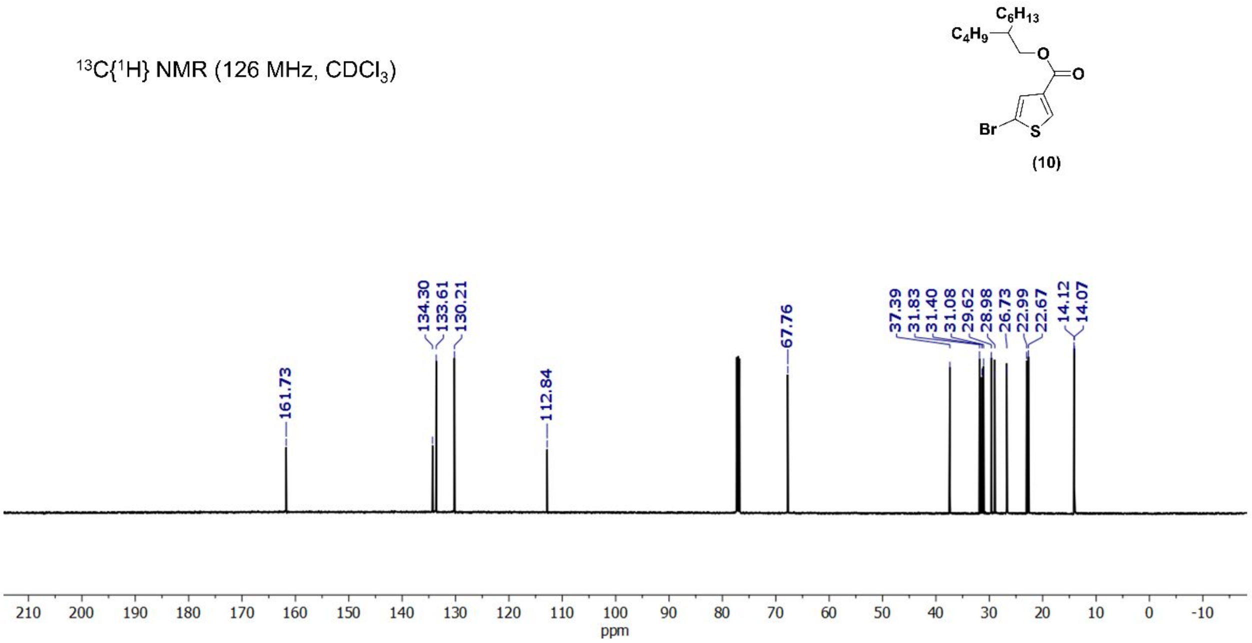

Figure S23. $^{13}C\{^1H\}$ NMR of 2-butyloctyl 5-bromothiophene-3-carboxylate (10).

Synthesis of bis(2-butyloctyl) [2,2'-bithiophene]-4,4'-dicarboxylate (11)

To a dry 250 mL round bottom flask were added 30 mL of dry dimethylformamide (DMF), 2-butyloctyl 5-bromothiophene-3-carboxylate (10) (2.8 g, 7.54 mmol), and a stir bar under positive argon pressure. Then, 958 mg (3.77 mmol, 0.5 equiv.) of bis(pinacolato)diboron was added followed by addition of 3 equivalents of potassium carbonate (K_2CO_3) (3.12 g, 22.6 mmol). Next, [1,1'-bis(diphenylphosphino)ferrocene]dichloropalladium(II), 1:1 complex with dichloromethane ($Pd(dppf)Cl_2 \cdot CH_2Cl_2$) (615 mg, 0.75 mmol, 0.1 equiv.) was added into the flask and the reaction mixture was left to stir overnight (16 hours) at 110 °C. The solvent was removed under reduced

pressure and the remaining contents were purified using silica gel column chromatography with 2:3 ratio of hexane:dichloromethane as mobile phase to afford a light yellow oil (2.2 g, 78%).^{1}H NMR (500 MHz, $CDCl_3$), δ (ppm): 7.98 (d, J = 1.3 Hz, 2H), 7.57 (d, J = 1.3 Hz, 2H), 4.19 (d, J = 5.8 Hz, 4H), 1.78-1.73 (m, 2H), 1.42-1.26 (m, 32H), 0.92-0.86 (m, 12H). $^{13}C\{^{1}H\}$ NMR (126 MHz, $CDCl_3$), δ (ppm): 162.50, 136.95, 134.64, 131.69, 124.59, 67.78, 37.41, 31.84, 31.44, 31.12, 29.63, 28.99, 26.75, 23.00, 22.67, 14.11, 14.09. NMR spectra are consistent with a previous report.[7]

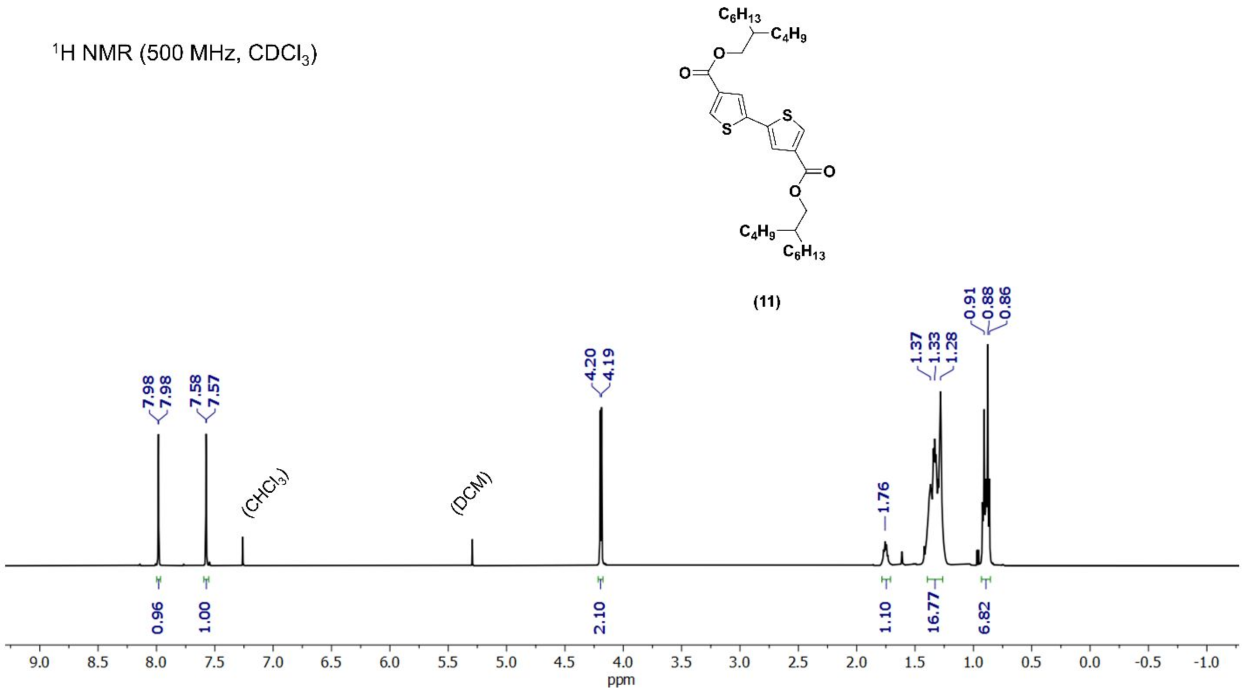

Figure S24. ^{1}H NMR of bis(2-butyloctyl) [2,2'-bithiophene]-4,4'-dicarboxylate (11).

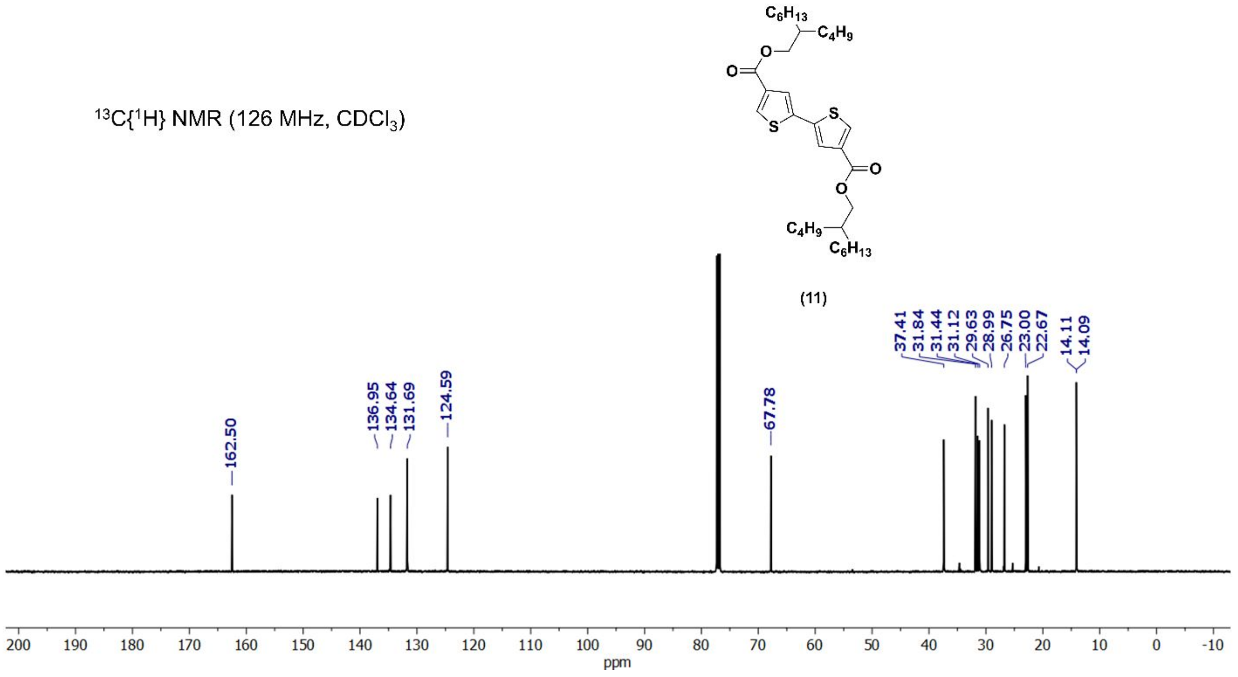

Figure S25. $^{13}C\{^{1}H\}$ NMR of bis(2-butyloctyl) [2,2'-bithiophene]-4,4'-dicarboxylate (11).

Synthesis of bis(2-butyloctyl) 5,5'-dibromo-[2,2'-bithiophene]-4,4'-dicarboxylate (12):

To a dry 250 mL round bottom flask were added 50 mL of chloroform, bis(2-butyloctyl) [2,2'-bithiophene]-4,4'-dicarboxylate (11) (1.72 g, 2.92 mmol), and a stir bar under positive argon pressure. Subsequently, 20 mL of trifluoroacetic acid (TFA) was added to the reaction flask. N-Bromosuccinimide (NBS) (1.30 g, 7.3 mmol, 2.5 equiv.) was added over 20 minutes to the flask at room temperature. The reaction mixture was left to stir overnight (16 hours) at room temperature. After this, the reaction was quenched by adding 50 mL of distilled water. The reaction mixture was extracted with 100 mL of DCM and the extract was washed with water followed by a brine solution. The organic layer was dried over sodium sulfate and the salt was filtered out. The solvent was removed under reduced pressure and the remaining contents were purified using silica

gel column chromatography with a 2:1 ratio of hexane:dichloromethane as mobile phase to afford a light yellow oil (1.53 g, 70%). ^{1}H NMR (500 MHz, $CDCl_3$), δ (ppm): 7.34 (s, 2H), 4.20 (d, J = 5.6 Hz, 4H), 1.77-1.72 (m, 2H), 1.42-1.25 (m, 32H), 0.91-0.85 (m, 12H). ^{13}C{^{1}H} NMR (126 MHz, $CDCl_3$), δ (ppm): 161.60, 135.27, 132.34, 126.03, 118.91, 68.06, 37.33, 31.85, 31.39, 31.06, 29.64, 28.97, 26.74, 23.01, 22.68, 14.13, 14.10. NMR spectra are consistent with previous report.[7]

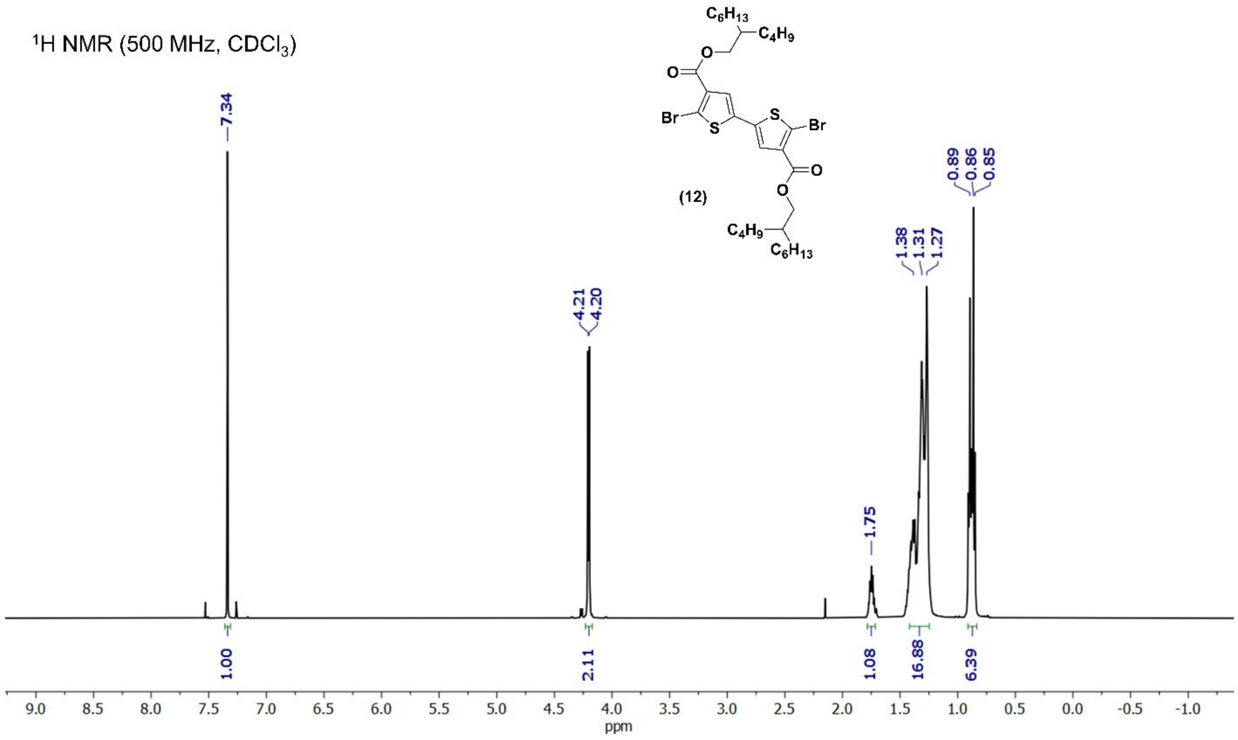

Figure S26. ^{1}H NMR of bis(2-butyloctyl) 5,5'-dibromo-[2,2'-bithiophene]-4,4'-dicarboxylate (4).

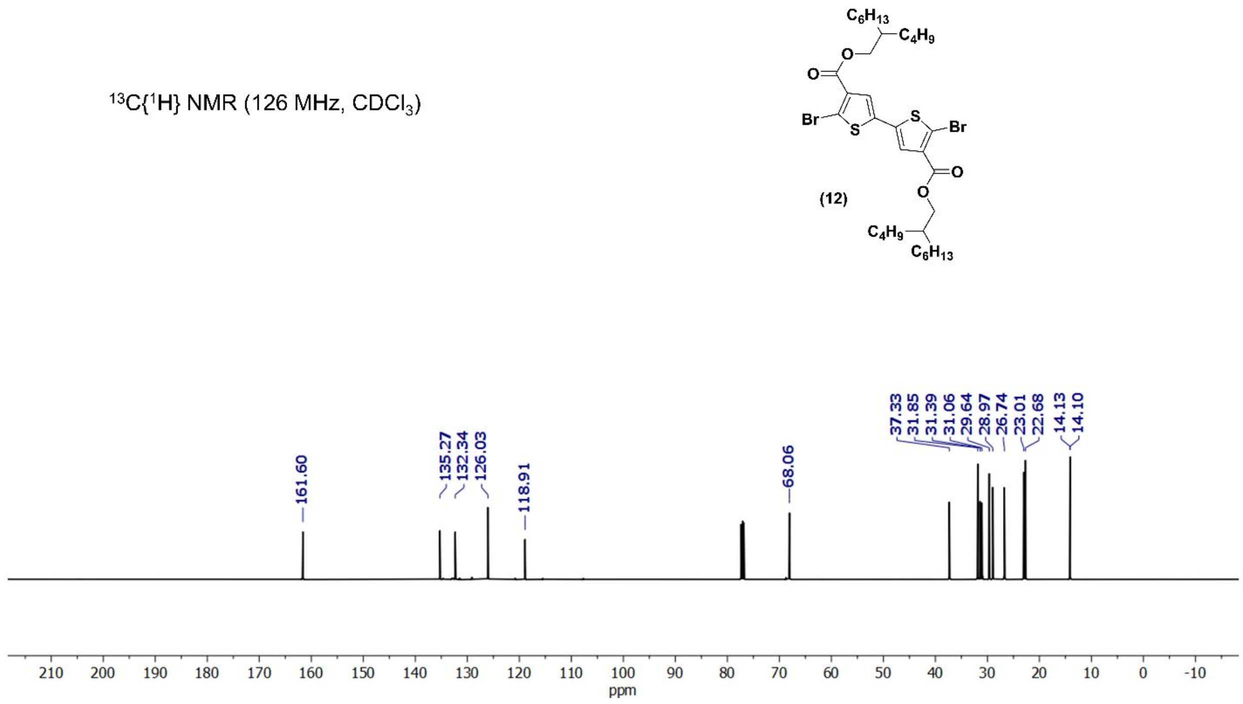

Figure S27. $^{13}C\{^1H\}$ NMR of bis(2-butyloctyl) 5,5’-dibromo-[2,2’-bithiophene]-4,4’-dicarboxylate (4).

Synthesis of bis(2-butyloctyl) [2,2':5',2'':5'',2'''-quaterthiophene]-3',4''-dicarboxylate (13):

To a dry 250 mL round bottom flask were added 40 mL of DMF, bis(2-butyloctyl) 5,5’-dibromo-[2,2’-bithiophene]-4,4’-dicarboxylate (12) (1.86 g, 2.59 mmol), and a stir bar under positive argon pressure. Subsequently, 2-(tributylstannyl)thiophene (2.03 g, 5.44 mmol, 2.1 equiv.) was added using a syringe to the flask at room temperature followed by addition of tetrakis(triphenylphosphine)palladium(0) ($Pd(PPh_3)_4$ (178 mg, 0.06 equiv.). The reaction mixture was left to stir overnight (16 hours) at 125 °C. After this, the reaction was quenched by adding 50 mL of distille water. The reaction mixture was extracted with 100 mL of DCM and the extract was washed with water and a brine solution. The organic layer was dried over sodium sulfate and

the salt was filtered out. The solvent was removed under reduced pressure and the remaining contents were purified using silica gel column chromatography with 2:1 ratio of hexane:dichloromethane as mobile phase to afford a light yellow solid (1.8 g, 92%). ^{1}H NMR (500 MHz, $CDCl_3$), δ (ppm): 7.53 (s, 2H), 7.46 (dd, J = 3.7, 1.2 Hz, 2H), 7.42 (dd, J = 5.2, 1.2 Hz, 2H), 7.08 (dd, J = 5.1, 3.6 Hz, 2H), 4.16 (d, J = 5.7 Hz, 4H), 1.71-1.66 (m, 2H), 1.32-1.24 (m, 32H), 0.91-0.86 (m, 12H). $^{13}C\{^{1}H\}$ NMR (126 MHz, $CDCl_3$), δ (ppm): 162.93, 142.16, 133.78, 133.33, 129.56, 128.70, 128.09, 127.28, 126.87, 67.91, 37.31, 31.85, 31.32, 31.00, 29.64, 28.96, 26.72, 23.01, 22.70, 14.14, 14.12. HR-MS (APCI,) m/z for $C_{42}H_{58}O_4S_4$ theoretical (M+H): 755.3290, found (M+H): 755.3284.

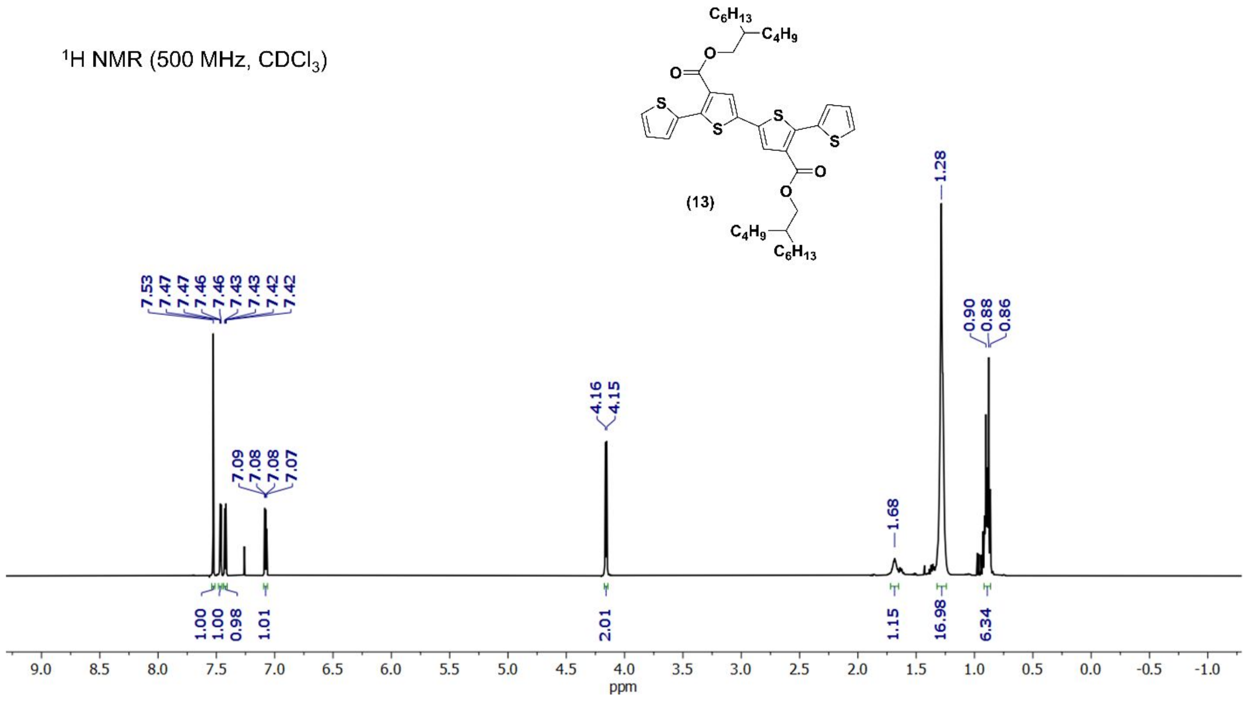

Figure S28. ^{1}H NMR of bis(2-butyloctyl) [2,2':5',2'':5'',2'''-quaterthiophene]-3',4''-dicarboxylate (13).

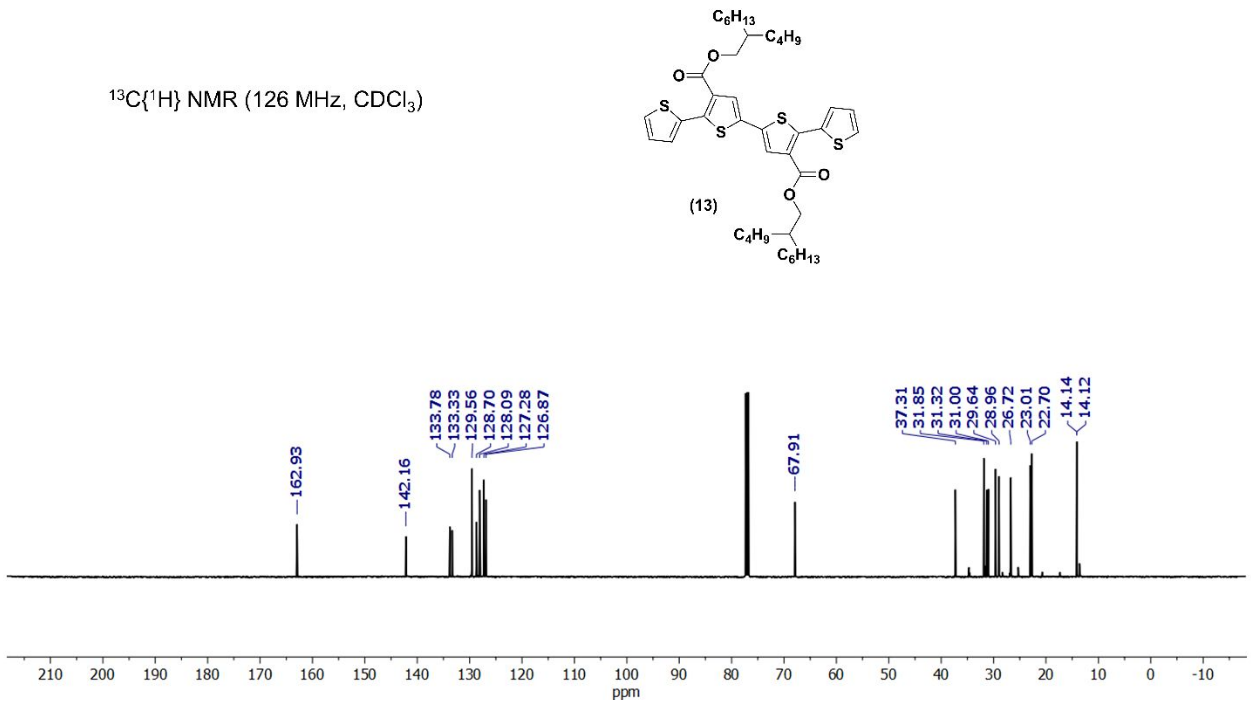

Figure S29. $^{13}C\{^1H\}$ NMR of bis(2-butyloctyl) [2,2':5',2'':5'',2'''-quaterthiophene]-3',4''-dicarboxylate (13).

Synthesis of bis(2-butyloctyl) 5,5'''-dibromo-[2,2':5',2'':5'',2'''-quaterthiophene]-3',4''-dicarboxylate (14):

To a dry 250 mL round bottom flask were added 50 mL of DMF, bis(2-butyloctyl) [2,2':5',2'':5'',2'''-quaterthiophene]-3',4''-dicarboxylate (13) (865 mg, 1.15 mmol), and a stir bar under positive argon pressure. Subsequently, NBS (428 mg, 2.41 mmol, 2.1 equiv.) was added over 20 minutes to the flask at room temperature. The reaction mixture was left to stir overnight (16 hours) at room temperature. After this time, the reaction was quenched by adding 50 mL of distilled water. The reaction mixture was extracted with 100 mL of DCM and the extract was washed with water and a brine solution.The organic layer was dried over sodium sulfate and the salts was filtered out. The

solvent was removed under reduced pressure and the remaining contents were purified using silica gel column chromatography with 2:1 ratio of hexane:dichloromethane as mobile phase to afford a yellow solid (840 mg, 80%). ^{1}H NMR (700 MHz, $CDCl_3$), δ (ppm): 7.49 (s, 2H), 7.20 (d, J = 3.9 Hz, 2H), 7.03 (d, J = 3.9 Hz, 2H), 4.17 (d, J = 5.8 Hz, 4H), 1.73-1.68 (m, 2H), 1.32-1.25 (m, 32H), 0.91-0.86 (m, 12H). ^{13}C{^{1}H} NMR (176 MHz, CDCl3) δ (ppm): 162.77, 141.33, 134.70, 133.64, 129.90, 129.59, 128.69, 126.96, 115.77, 68.12, 37.30, 31.85, 31.33, 31.01, 29.65, 28.95, 26.72, 23.01, 22.69, 14.13, 14.12. HR-MS (APCI,) m/z for $C_{42}H_{56}O_4S_4Br_2$ theoretical (M+H): 911.1501, found (M+H): 911.1501.

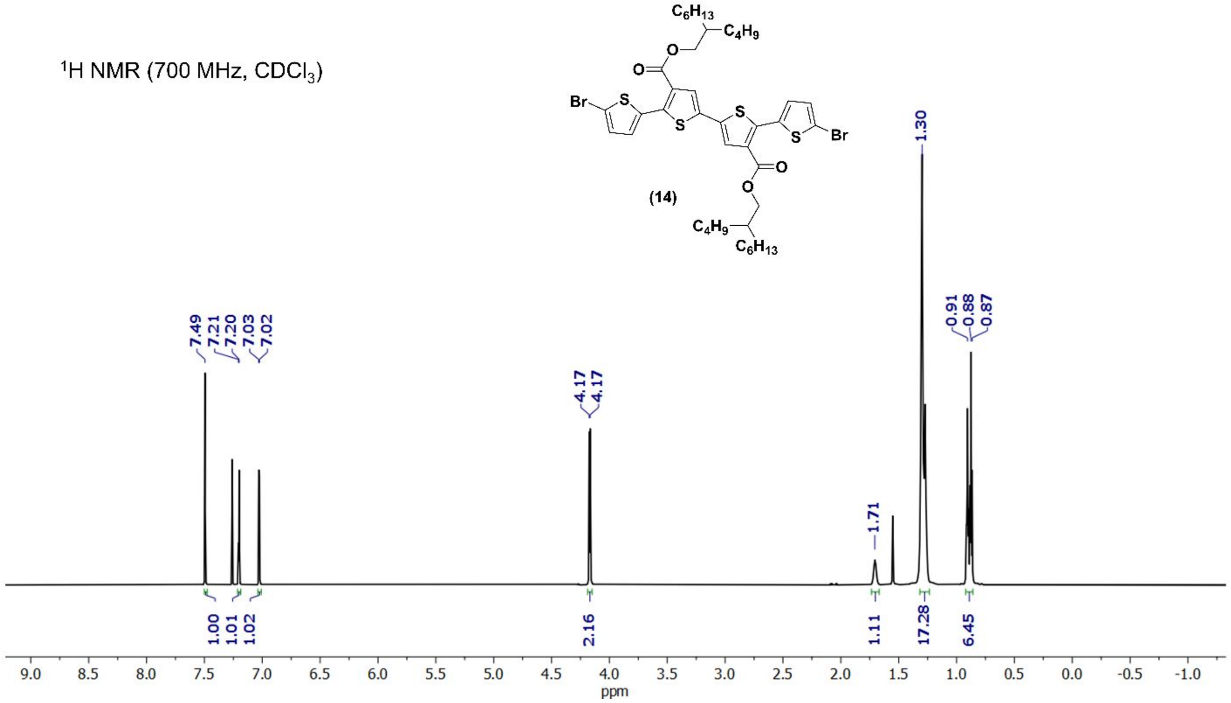

Figure S30. ^{1}H NMR of bis(2-butyloctyl) 5,5'''-dibromo-[2,2':5',2'':5'',2'''-quaterthiophene]-3',4''-dicarboxylate (14).

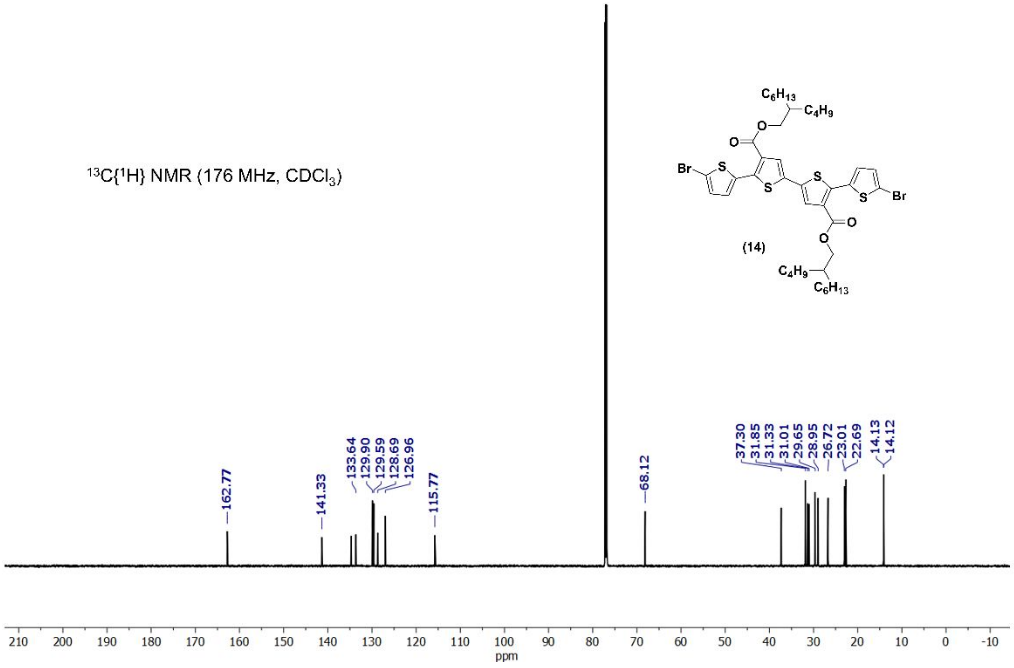

Figure S31. $^{13}C\{^1H\}$ NMR of bis(2-butyloctyl) 5,5'''-dibromo-[2,2':5',2'':5'',2'''-quaterthiophene]-3',4''-dicarboxylate (14).

3-Polymerization details and general polymer characterizations

General polymerization and purification procedures:

Bisstannyl monomer (molecule 15) (220 mg, 0.226 mmol, 1 equiv.) and dibromo quaterthiophene monomer (207 mg, 0.226 mmol, 1 equiv. of molecule 4, molecule 8, or molecule 14 respectively for PM7-D3, PM7-D4, and PM7-D5) were added to a freshly dried and cooled round bottom flask containing a stir bar. Then the flask was taken to a glove box where dipalladium-tris(dibenzylideneacetone)chloroform complex, $Pd_2(dba)_3 \cdot CHCl_3$ as the catalyst (7.0 mg, 0.00678 mmol, 0.03 equiv.), tris(o-tolyl)phosphine as the ligand, and 4 mL of toluene as the polymerization solvent were added to the flask. The reaction flask then was taken out of the glove box and was

immersed in an oil bath set at 105 °C and stirred for 16 hours. At the end of this time, a small amount of palladium scavenger (diethylammonium diethyldithiocarbamate) was added, and the temperature was dropped to 90 °C. After stirring for 1 hour at 90 °C, the solution was brought to room temperature and the crude polymer was precipitated into stirring cold methanol upon cooling to room temperature. The crude polymer was further purified by Soxhlet washing with methanol (24 hours), Acetone (24 hours), hexane (24 hours), and the purified polymer was obtained from Soxhlet extraction using chloroform. The purified polymer then reprecipitated into cold methanol and collected by vacuum filtration on a nylon membrane with pore size of 45 µm.

PM7-D3: Dark-red solid (309 mg, 93%). M_n: 64.6 kg/mol, M_w: 148.6 kg/mol, *Đ*: 2.30 (GPC in 1, 2, 4-trichlorobenzene vs polystyrene). ^{1}H NMR (400 MHz, *o*-$Cl_2C_6D_4$, 110 °C): δ (ppm) 8.08-7.78 (m, 4H), 7.71-7.53 (m, 4H), 7.32 (s, 2H), 4.46 (m, 4H), 3.09 (m, 4H), 2.03 (m, 4H), 1.78-1.41 (m, 48H), 1.25-1.00 (m, 24H). Anal. calcd. for $C_{76}H_{94}Cl_2O_4S_8$: C (65.25%), H (6.77%), S (18.33%); Found: C (65.49%), H (6.88%), S (18.20%).

^{1}H NMR (400 MHz , o-$Cl_2C_6D_4$)

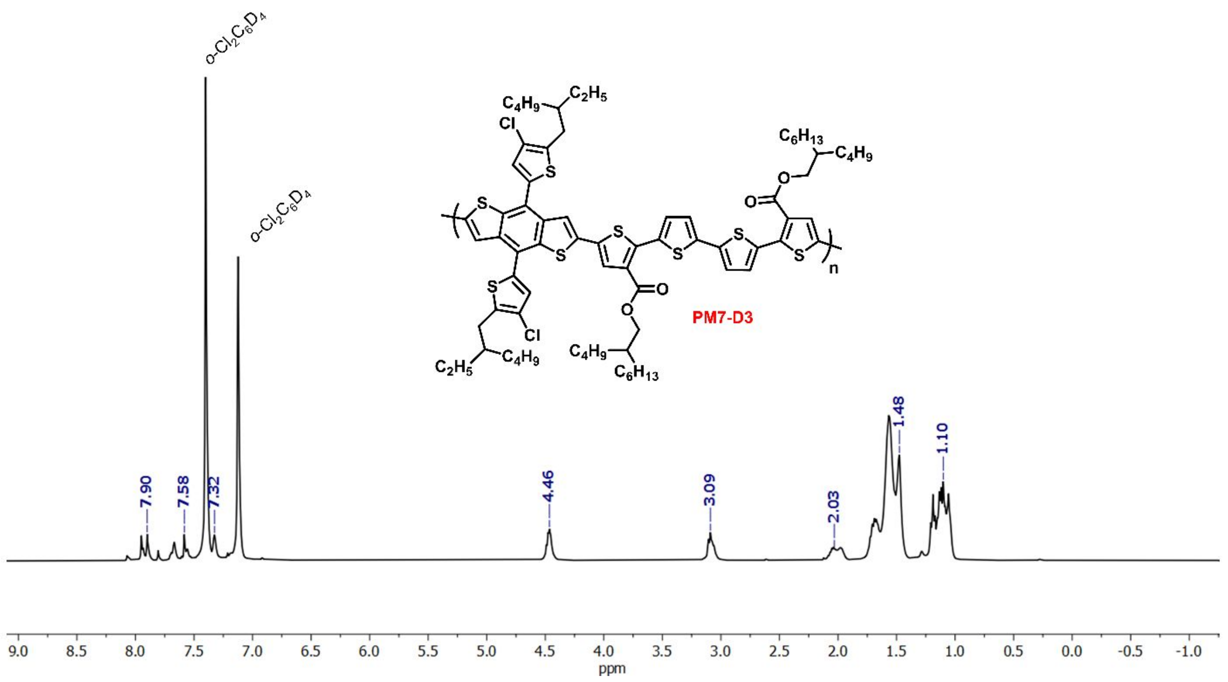

Figure S32.^{1}H NMR spectra of PM7-D3 in o-dichlorobenzene-D4 at 110 °C.

PM7-D4: Dark red flexible film (316 mg, 95%). M_n: 64.3 kg/mol, M_w: 165.3 kg/mol, *Đ*: 2.57 (GPC in 1, 2, 4-trichlorobenzene vs. polystyrene). ^{1}H NMR (400 MHz, o-$Cl_2C_6D_4$, 110 °C): δ (ppm) 7.85-7.59 (m, 4H), 7.32 (s, 2H), 7.10-6.96 (m, 4H), 4.14 (m, 4H), 3.09 (m, 4H), 1.88 (m, 4H), 1.78-1.25 (m, 48H), 1.25-0.80 (m, 24H). Anal. calcd. for $C_{76}H_{94}Cl_2O_4S_8$: C (65.25%), H (6.77%), S (18.33%); Found: C (65.51%), H (6.90%), S (18.04%).

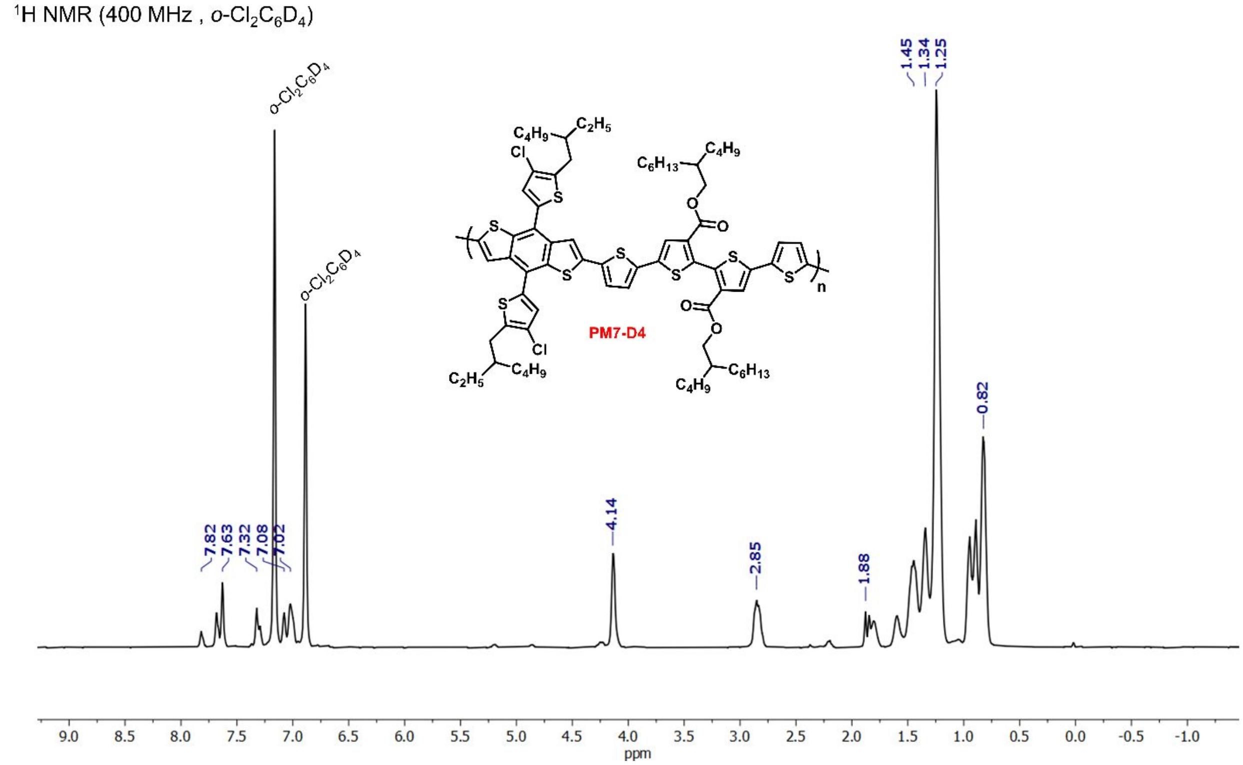

Figure S33.^{1}H NMR spectra of PM7-D4 in o-dichlorobenzene-D4 at 110 °C.

PM7-D5: Dark-red solid (326 mg, 98%). M_n: 26.1 kg/mol, M_w: 54.5 kg/mol, *Đ*: 2.09 (GPC in 1, 2, 4-trichlorobenzene vs. polystyrene). ^{1}H NMR (400 MHz, *o*-$Cl_2C_6D_4$, 110 °C): δ (ppm) 7.77 (s, 2H), 7.61 (s, 2H), 7.50 (s, 2H), 7.38 (s, 2H), ~7.21 (with solvent peak, 2H), 4.26 (m, 4H), 2.89 (m, 4H), 1.90-1.70 (m, 4H), 1.57-1.19 (m, 48H), 0.99-0.83 (m, 24H). Anal. calcd. for $C_{76}H_{94}Cl_2O_4S_8$: C (65.25%), H (6.77%), S (18.33%); Found: C (65.53%), H (6.80%), S (18.35%).

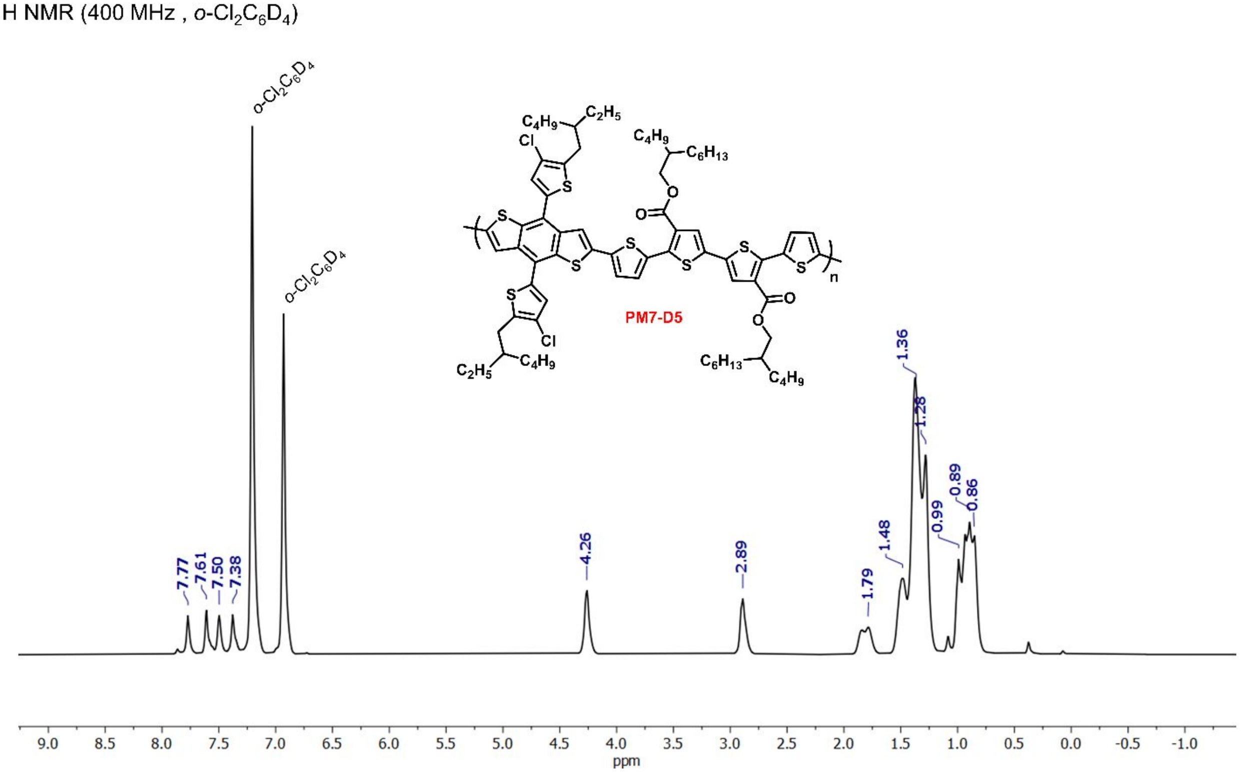

Figure S34.^{1}H NMR spectra of PM7-D5 in o-dichlorobenzene-D4 at 110 °C.

Table S1. Basic synthesis characterization of the PM7-Dx (x = 3, 4, and 5) polymers.

**Polymer**	**M_n (kg mol^{-1})**	**Đ**	**yield**	**Theoretical elemental composition**	**Measured elemental composition**
PM7-D3	64.6	2.30	93%	C: 65.25 % H: 6.77 % S: 18.33	C: 65.49 % H: 6.88 % S: 18.20 %
PM7-D4	64.3	2.57	98%	C: 65.25 % H: 6.77 % S: 18.33 %	C: 65.51 % H: 6.90 % S: 18.04 %
PM7-D5	26.1	2.09	95%	C: 65.25 % H: 6.77 % S: 18.33 %	C: 65.53 % H: 6.80 % S: 18.35 %

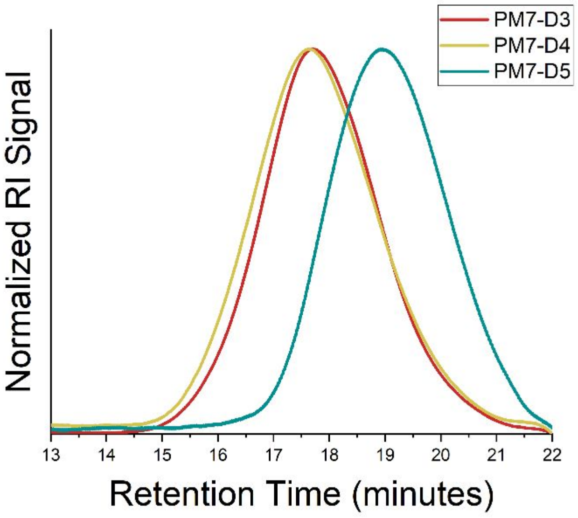

Figure S35. HT-GPC traces of PM7-D3, PM7-D4, and PM7-D5.

4- DFT calculations

Computational approaches

Geometry optimizations of isolated oligomers of the PM7-Dx (x = 3, 4, 5) series of polymers were performed using the default long-range corrected ωB97X-D functional and the 6-31G(d, p) basis set. For the evaluation of ionization potentials (IPs), electron affinities (EAs), and excited-state properties, the range separation parameter was optimized through minimizing $J(\omega) = (E_{\mathrm{HOMO}} + \mathrm{IP})^2 + (E_{\mathrm{LUMO}} + \mathrm{EA})$ in an implicit dielectric medium using the polarizable continuum model (PCM) with $\varepsilon = 4.0$ as the dielectric constant of the medium, which is a typical value for organic semiconductors with polarizable groups (*i.e.*, carbonyl groups in this case). The optimal ω values for PM7-D3, PM7-D4, and PM7-D5 symmetric (A:D:A:D:A) dimers, with D standing here for benzodithiophene and A for the thiophene segments, are 0.003, 0.005, and 0.003 Bohr^{-1}, respectively (for the sale of consistency in subsequent calculations, 0.003 Bohr^{-1} was used as the optimal ω across the PM7-Dx series). Here, the vertical IP is calculated as the energy difference between the neutral ground-state structure and the cation at the geometry of the neutral structure, and the vertical EA is calculated as the energy difference between the neutral ground-state structure and the anion at the geometry of the neutral structure. Excited-state properties including the lowest singlet excited state (S_1) energies were evaluated at the time-dependent DFT (TD-DFT) level using the symmetric dimers. The torsional scans of selected dihedrals along the backbone were performed from 0° to 360° at 10° intervals, where each dihedral was fixed at the scanned angle and the rest of the structure was fully geometry relaxed. The Gaussian 16 package[8] was used for all electronic-structure calculations.

The computational approach followed for obtaining heterojunction interfaces usually calls for extensive molecular dynamics (MD) simulations; donor:acceptor (D:A) complexes are then extracted from MD snapshots for the further investigation of their electronic properties. Here, however, we followed a more straightforward procedure as we based our analysis on model interfacial D:A complexes obtained via geometry optimizations at the DFT level with L8-BO considered as the acceptor molecule. The initial model complexes were prepared such that an electron-rich moiety (BDT) from the donor oligomers was placed on top of an electron-poor moiety (TTP, thienothienopyrrolo) of the acceptor along the central part of the backbones; the complexes were then allowed to fully relax. The minimum separation between the complexes was initially set to ~3.8 Å; in the optimized geometries, this distance was found to be reduced to ~3.5 Å. The geometry optimizations of all isolated model D:A complexes were carried out with the B3LYP functional and the 6-31G(d) basis set, using the Gaussian 16 package. This was followed by TD-DFT calculations with the tuned long-range corrected ωB97X-D functional (ω = 0.010 $Bohr^{-1}$ for the D:A complex) and the 6-31G(d, p) basis set, using the polarizable continuum model (PCM); here, ε was set to 3.5 ($= \frac{\varepsilon_A + \varepsilon_D}{2}$) as the dielectric constant of the medium. The hole and electron natural transition orbitals (NTOs) were calculated for the lowest two CT (charge-transfer) and LE (local-exciton) states. The TD-DFT and NTO calculations were carried out with the Q-Chem 5.4 package,[9] and the electronic couplings were obtained using the generalized Mulliken-Hush approach[10] as implemented in the same package. The reorganization energies related to exciton dissociation were calculated using the adiabatic potential energy surfaces of the molecular states involved in this process (see Ref. 11 for details). The exciton dissociation rate constants, $k_{LE \to CT}$, were calculated in the framework of the semi-classical Marcus equation;[12] in these calculations, we took a value of 0.1 eV for the reorganization energy due to the medium.

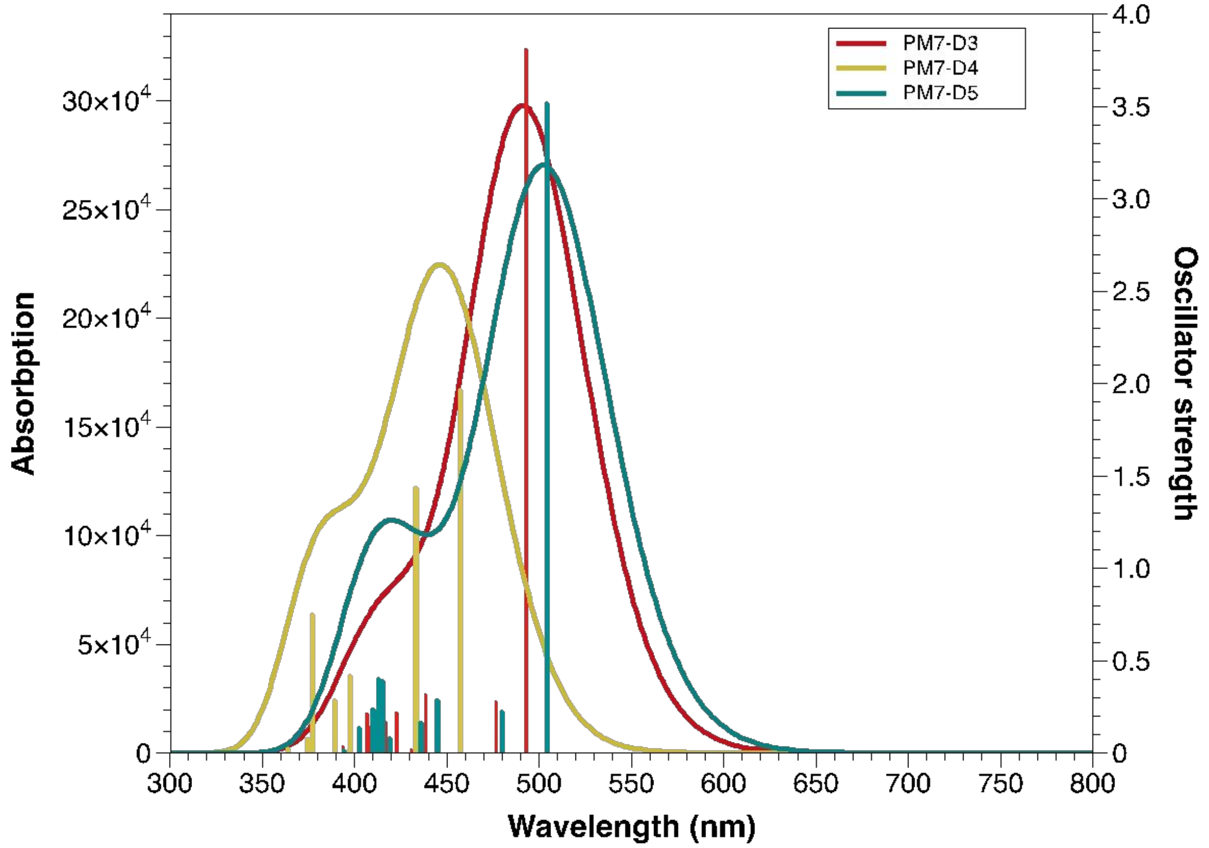

Figure S36. Simulated absorption spectra of PM7-D3 (red), PM7-D4 (yellow), and PM7-D5 (blue) symmetric dimer models, as calculated at the TD-DFT ωB97X-D/6-31G(d,p) level of theory (ω=0.003 Bohr^{-1}, ε=4.0).

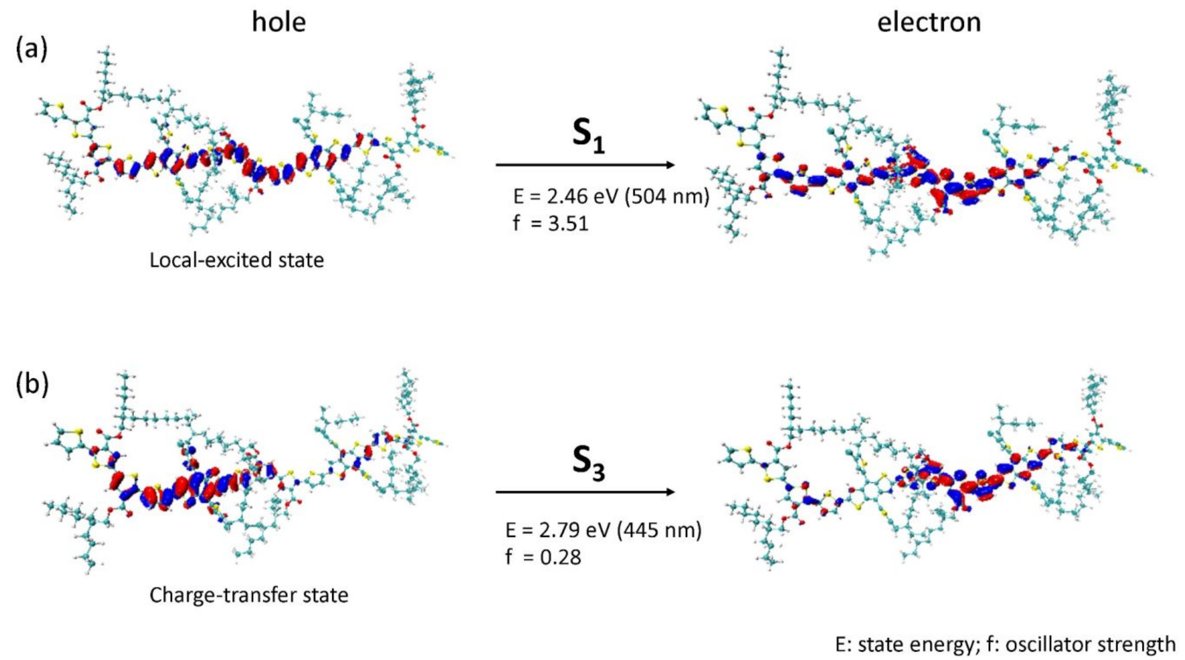

Figure S37. Natural transition orbitals of representative electronic transitions found in the UV-vis spectra: (a) at absorption maximum and (b) within the shoulder region (350 -450 nm), for the case of PM7-D5.

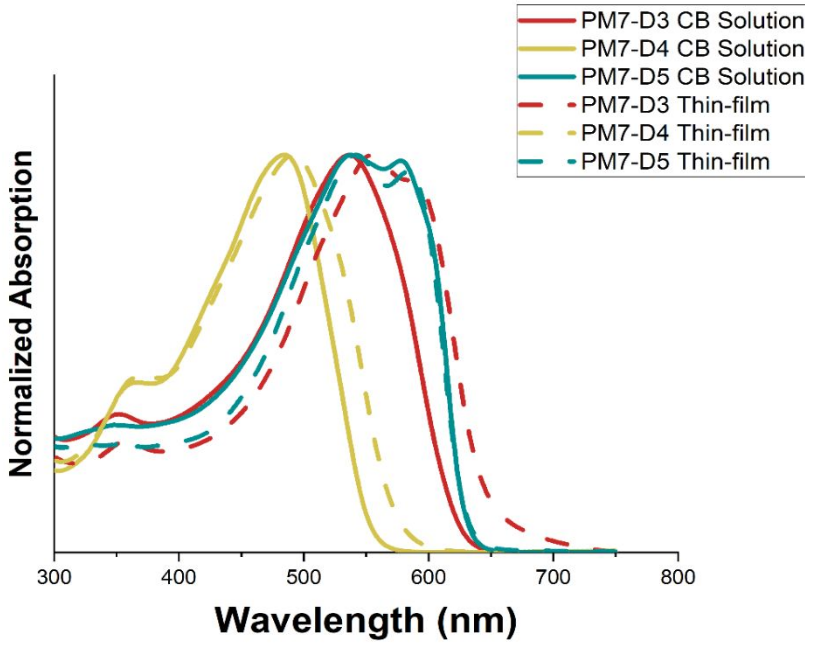

Figure S38. UV-Vis absorption in solution (solid lines) and thin-film (dashed lines) for the PM7-Dx polymers.

Table S2. Summary of optical properties of PM7-Dx polymers. Results for PM7 D1 and PM7 D2 are from the literature.[1,13]

	Solution λ_{max}	Solid-state λ_{max}	Thin-film onset of absorption	DFT Abs prediction for dimers	λmax solution to thin film shift	Opt bandgap	Echem bandgap	Exciton binding energy
**PM7 D1**	525 nm	548 nm	1.93 eV	-	23 nm	1.93	2.40	0.47
**PM7 D2**	525 nm	540 nm	1.96 eV	-	15 nm	1.96	2.44	0.48
**PM7-D3**	538 nm	555 nm	1.92 eV	493 nm (2.51 eV)	17 nm	1.92	2.41	0.49
**PM7-D4**	483 nm	492 nm	2.15 eV	458 nm (2.71 eV)	9 nm	2.15	2.50	0.35
**PM7-D5**	538 nm 578 nm	542 nm	1.97 eV	504 nm (2.46 eV)	4 nm	1.97	2.20	0.23

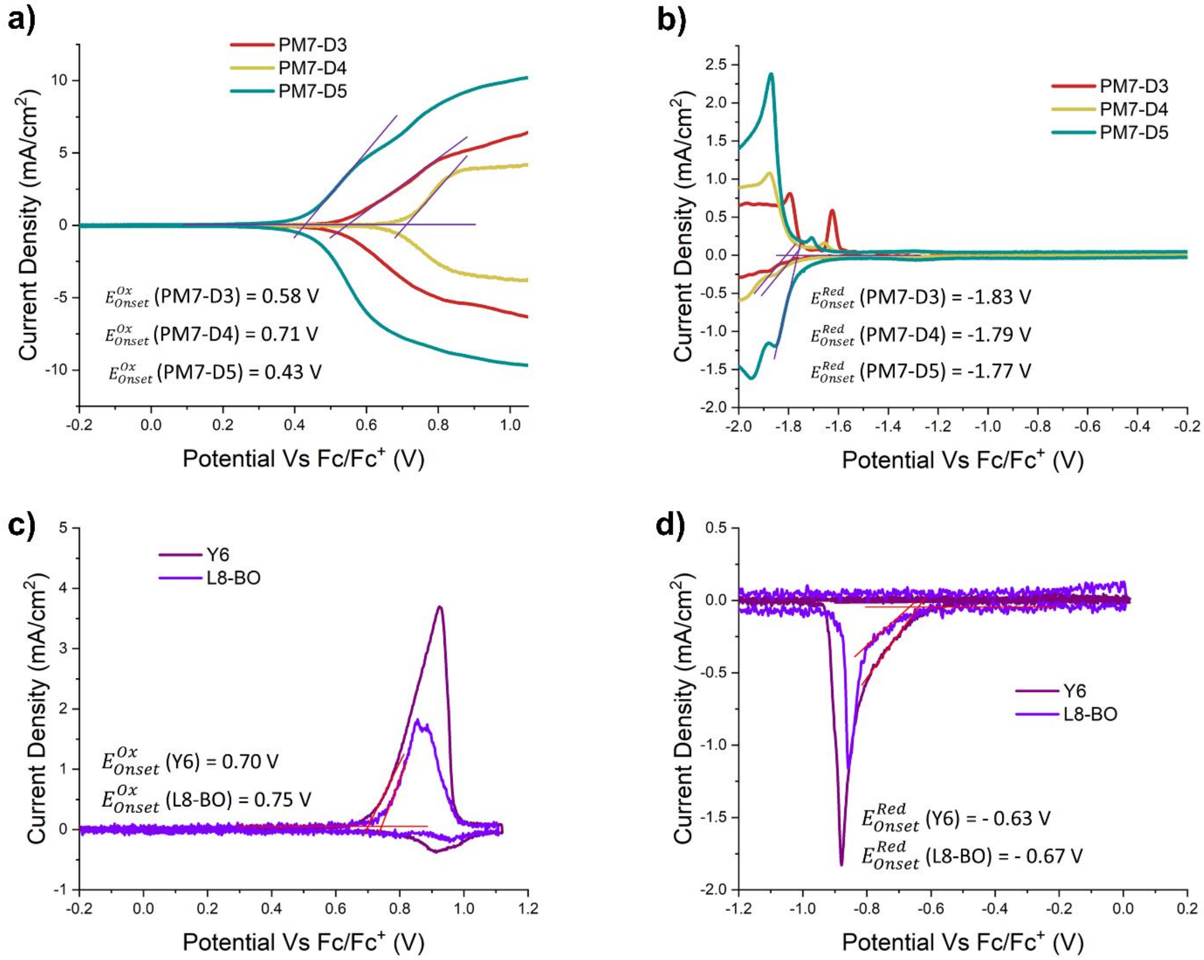

Figure S39. Results of DPV measurements of ionization potential (a and c) and electron affinity (b and d) for PM7-Dx and molecular acceptors. The IP and EA positive values were calculated by assuming saturated calomel electrode (SCE) versus vacuum with respect to Fc/Fc+ to be 5.12 eV using the equation IP/EA = +e(Eox/red + 5.12).

5- Solution processing optimization for device screening

Device fabrication details (PM7-Dx:Y6)

OPV devices were fabricated in an inverted device architecture (ITO)/ZnO/PM7-Dx:Y6/MoO_3/Ag. ITO substrates were purchased from Latech Scientific Supply Pte. Ltd., sheet resistance 10 Ω/sq. They were cleaned with dish soap and distilled water, followed by sonication in distilled water, acetone, isopropyl alcohol, subsequently for 10 minutes each. For the electron transport layer, a ZnO solution was made with 0.11 M Zn acetate dihydrate and 0.11 M ethanolamine combined in 2-methoxyethanol (Sigma Aldrich). This solution was stirred overnight at room temperature, then filtered with a 0.45 µm PTFE syringe filter before use. The ZnO solution was deposited on the cleaned ITO substrates by spin-coating for 30 seconds at 4000 rpm in ambient atmosphere to get a layer thickness of ~30 nm. After spin-coating, the ZnO layer was annealed in air at 150 ˚C for 15 min followed by slow cooling to room temperature and brought into an argon filled glovebox for active layer deposition. The active layers were prepared at a concentration of 20 mg/ml (D3 and D4) and 19 mg/ml (D5) in chloroform with or without 5% chlorobenzene (v/v), using a 1:1.2 donor:acceptor ratio. The active layers were spin-coated at 1500 (D3, D5) and 5000 rpm (D4) to yield active layers of 97 nm (D3), 93 (D4) and 74 nm (D5). After annealing of the films at 100 °C for 10 minutes, MoO_3 (7 nm) and Ag (100 nm) electrodes were thermally evaporated at pressures < 10^{-6} in a Ångstrom Engineering deposition chamber to obtain 6 complete solar cell devices per substrate with an electrode overlap area of 0.07 cm^2. The J-V characteristics of all photovoltaic devices were evaluated under AM 1.5 G solar illumination (100 mW/cm^2) using a Keithley SMU 2410 with a Newport Thermal Oriel 94021 solar simulator calibrated with a reference silicon solar cell.

Table S3. Device fabrication screening results for PM7-Dx polymers. Active layer solutions consisted of a 1:1.2 polymer:MA weight ratio in chloroform, which is similar to the optimal conditions published for many PBDB-T derivatives. Devices made at Georgia Tech. (Thicknesses are 110 ± 10 nm, average of 8 cells.)

Polymer blend	Solvent system	J_{SC} (mA cm^{-2})	V_{OC} (V)	FF (%)	Average PCE (best) (%)
PM7-D3:Y6	CF	8.44 ± 0.67	0.84 ± 0.03	37.44 ± 3.30	2.78 ± 0.23 (3.21)
PM7-D3:Y6	CF + 5% CB	13.65 ± 0.63	0.83 ± 0.01	49.46 ± 1.90	5.90 ± 0.38 (6.48)
PM7-D4:Y6	CF	4.69 ± 0.28	0.86 ± 0.01	50.50 ± 1.69	1.50 ± 0.06 (1.60)
PM7-D4:Y6	CF + 5% CB	9.72 ± 0.35	0.82 ± 0.01	46.81 ± 0.69	3.92 ± 0.17 (4.21)
PM7-D5:Y6	CF	12.06 ± 0.87	0.84 ± 0.01	41.92 ± 0.90	4.45 ± 0.34 (5.47)
PM7-D5:Y6	CF + 5% CB	18.17 ± 0.39	0.76 ± 0.01	61.41 ± 1.78	8.92 ± 0.28 (9.30)

Device fabrication details (PM7-Dx:L8-BO)

OPV devices were fabricated in an inverted device architecture (ITO)/ZnO/PM7-Dx:L8-Bo/MoO_3/Ag. ITO substrates were purchased from South China Science & Technology Company Limited. They were cleaned with dish soap and distilled water, followed by sonication in distilled water, acetone, isopropyl alcohol, subsequently for 10 minutes each. The ZnO layers were prepared via spin-coating under ambient conditions at 4000 rpm. They were prepared from a solution of diethyl zinc in toluene (15%) mixed in a 1:2 ratio with THF. After spin-coating, the

films were annealed annealing at 150 °C for 25 minutes. The substrates were transferred into a nitrogen-filled glove box. The active layers were prepared at a concentration of 20 mg/ml (D3 and D4) and 19 mg/ml (D5) in chloroform with 5% chlorobenzene (v/v), using a 1:1.2 donor:acceptor ratio. The active layers were spin-coated at 1500 (D3, D5) and 5000 rpm (D4) to yield active layers of 97 nm (D3), 93 (D4) and 74 nm (D5). After annealing of the films at 100 °C for 10 minutes, MoO_3 (7 nm) and Ag (100 nm) electrodes were thermally evaporated at pressures < 10^{-6} in a Ångstrom Engineering deposition chamber using shadow masks with an area of 0.22 cm^2.

6- Morphology studies

Experimental Details:

Grazing-incidence wide-angle X-ray scattering (GIWAXS) of pristine polymer samples were prepared using either 15 mg/ml chloroform solution or 18mg/ml solution of chloroform with 5vol% chlorobenzene and 0.5vol% chloronaphthalene that was stirred at 30°C for 5 hours and then dynamically spin coated onto a UV ozone cleaned silicon wafer substrate spinning at 2000RPM for 40 seconds. GIWAXS measurements were performed at the National Synchrotron Light Source II using beamline 11-BM. X-rays with an energy of 13.5keV at an incident angle of 0.15 degrees were used to produce scattering profiles collected by a Pilatus 1M detector (Dectris). The sample-to-detector distance of 258mm was determined using a silver behenate standard. The detector images were transformed to scattering vector space (q-space), sin(Chi) corrected as described in literature[14] then integrated using the pyFAI and pygix libraries.[15] Scattering intensities of the linecuts were normalized by film thickness before fitting was performed in python using the lmfit

package with a combination of linear background, exponential decay background, and pseudo-Voigt scattering peaks.[16]

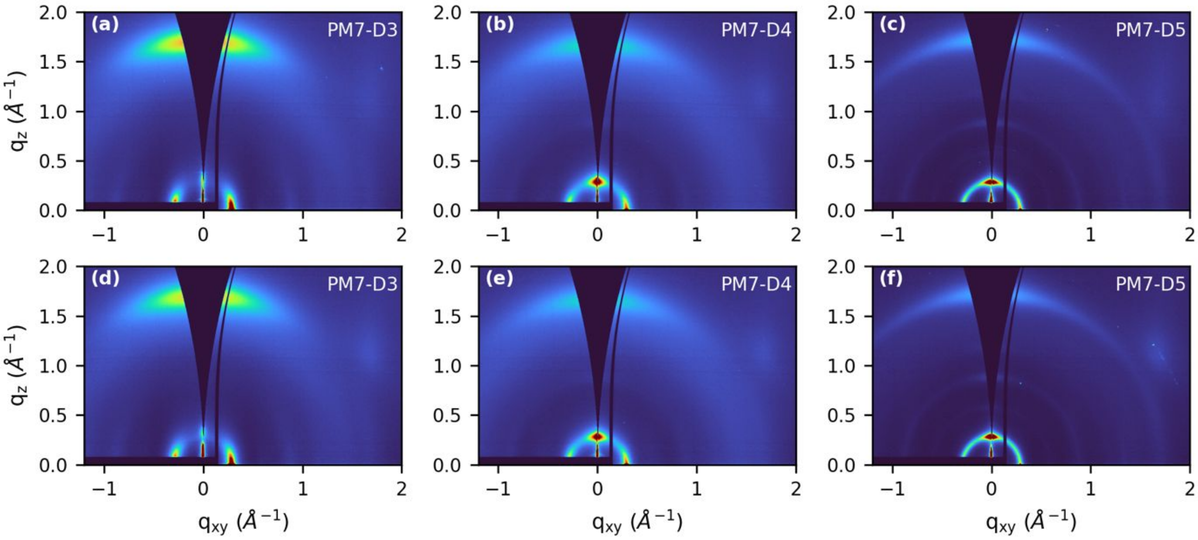

Figure S40. Reciprocal space maps from GIWAXS of neat polymer films. Measurements of duplicate films of (a, d) PM7-D3, (b, e) PM7-D4, and (c, f) PM7-D5 are shown above.

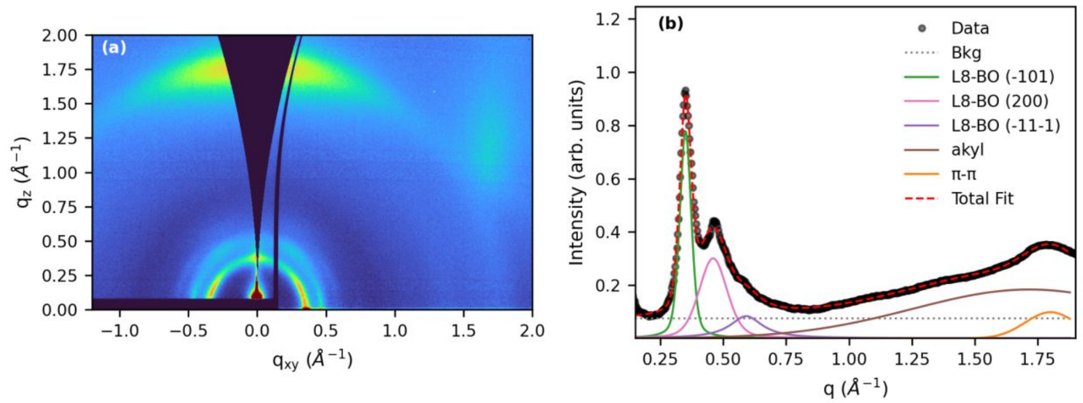

Figure S41. GIWAXS scattering from neat L8-BO acceptor film. (a) Reciprocal space map of scattering intensity. (b) Azimuthally integrated linecuts from 5 to 85° along with fits including peak components and a constant background overlayed.

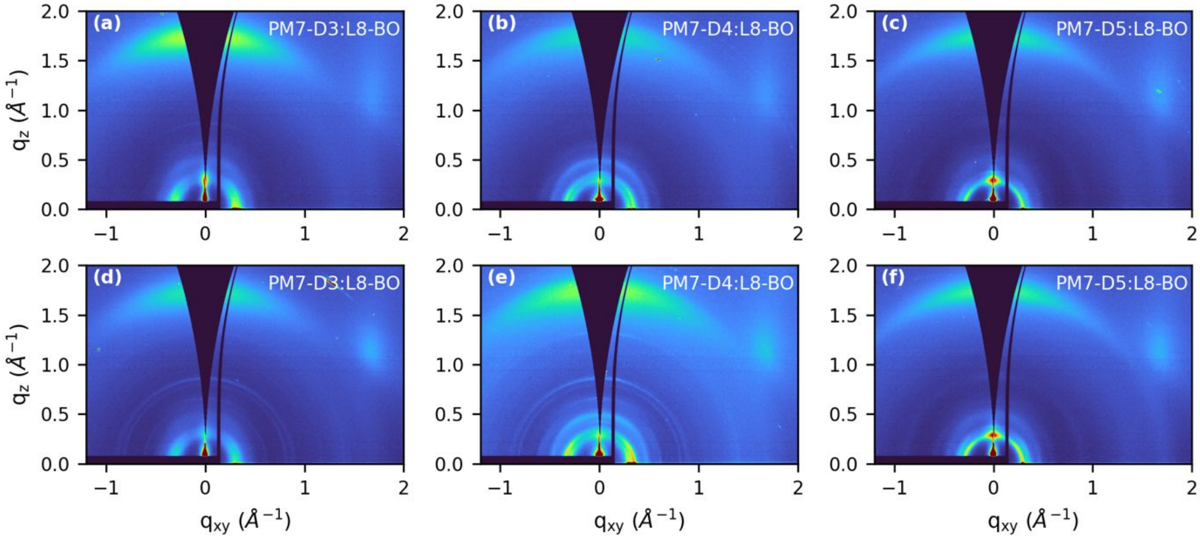

Figure S42. Reciprocal space maps from GIWAXS of bulk heterojunction polymer:L8-BO films. Measurements of duplicate films of (a, d) PM7-D3:L8-BO, (b, e) PM7-D4:L8-BO, and (c, f) PM7-D5:L8-BO are shown above.

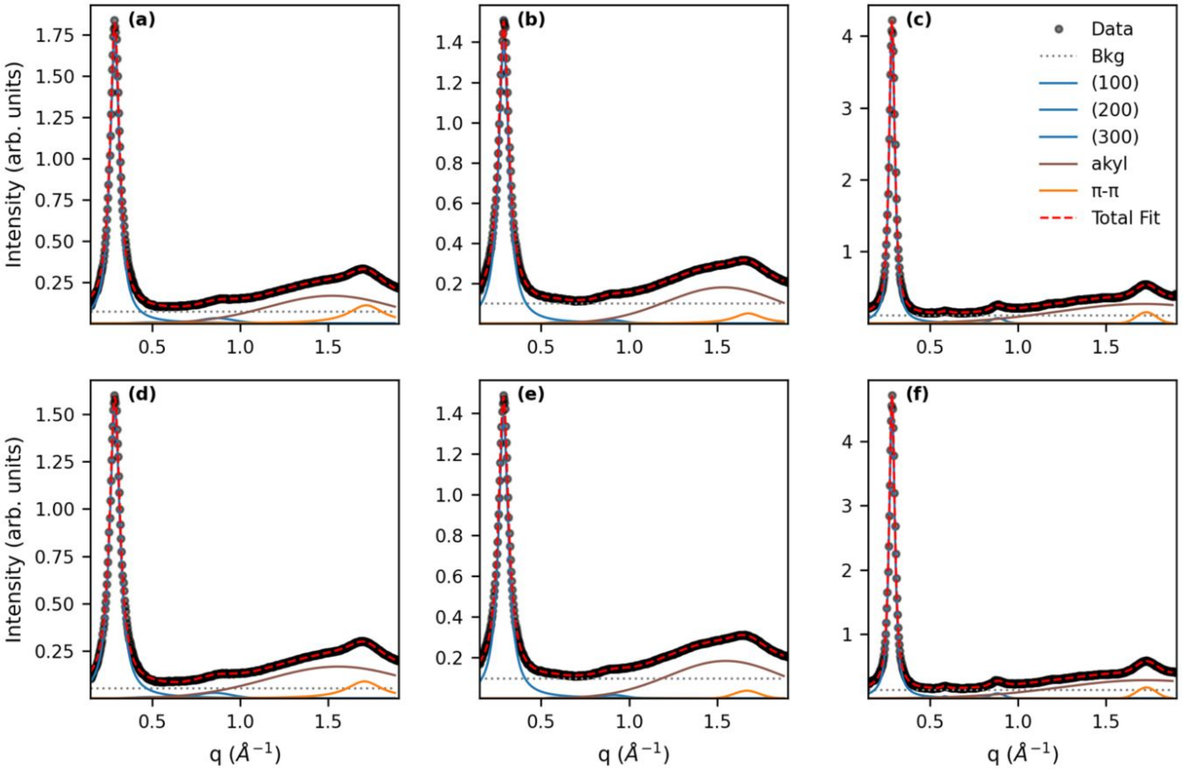

Figure S43. Azimuthally integrated linecuts from 5 to 85° for neat films of (a, d) PM7-D3, (b, e) PM7-D4, and (c, f) PM7-D5 are shown above along with their fits including peak components and

a linear background overlayed. All films exhibit the same stacking peaks with varying position, intensity, and width.

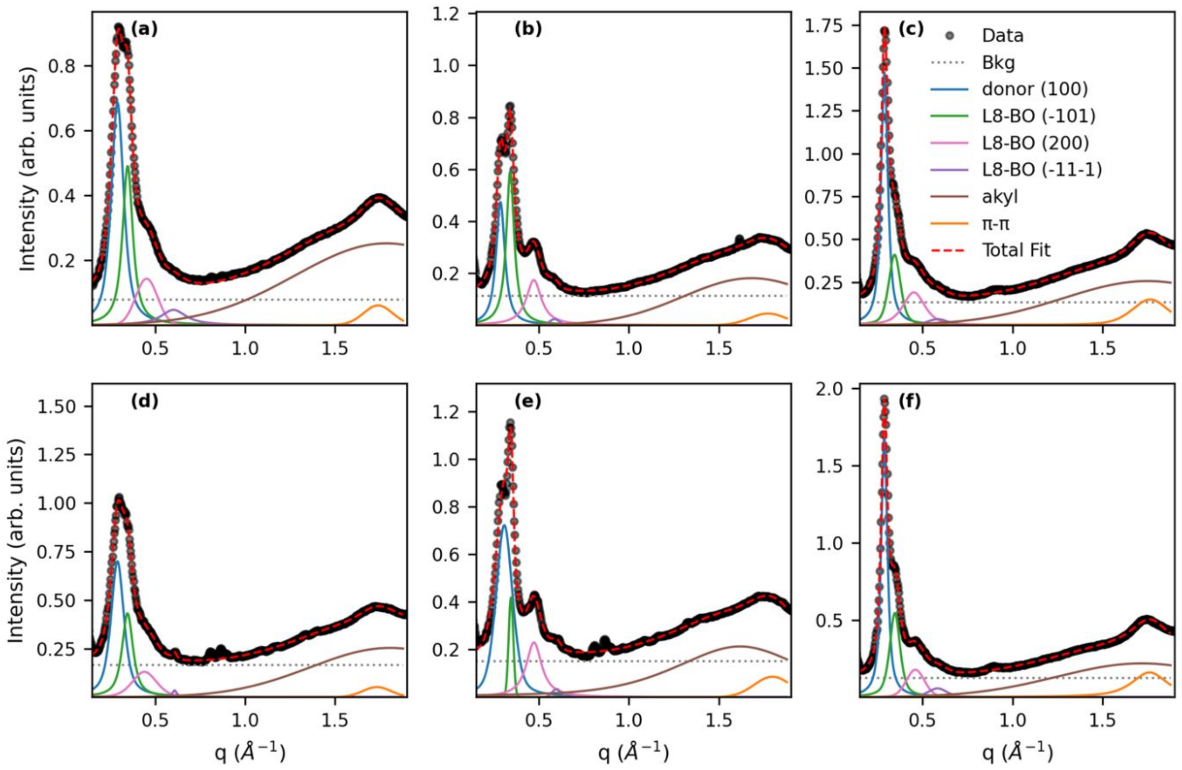

Figure S44. Azimuthally integrated linecuts from 5 to 85° for bulk heterojunction films of (a, d) PM7-D3:L8-BO, (b, e) PM7-D4:L8-BO, and (c, f) PM7-D5:L8-BO are shown above along with their fits including peak components and a constant background overlayed. All films exhibit the same stacking peaks with varying position, intensity, and width.

Table S4. Summary of the fit parameters of the GIWAXS linecuts. Values displayed are averaged between GIWAXS of two duplicate films, except for neat L8-BO where only a single film was measured. The range of values between duplicate films are also reported where available. For identifying the polymer peaks, we assume an orthorhombic unit cell (as is common). The L8-BO peaks are identified by the most closely matching Miller indices to a Y6 unit cell from existing literature oriented such that the (040) refers to π-π spacing.[17]

	donor lamella (100) peak			acceptor $(\bar{1}01)$ peak			π- π peak		
Sample	d (Å)	I (arb.)	L_c (Å)	d (Å)	I (arb.)	L_c (Å)	d (Å)	I (arb.)	L_c (Å)
PM7-D3	**21.87** ±0.01	**173** ±3	**84** ±3	-	-	-	**3.68** ±0.01	**32** ±7	**26** ±3
PM7-D4	**21.89** ±0.05	**152** ±4	**82** ±2	-	-	-	**3.76** ±0.01	**12** ±4	**31** ±2
PM7-D5	**22.08** ±0.01	**249** ±11	**145** ±2	-	-	-	**3.63** ±0.00*	**23** ±1	**43** ±1
PM7-D3:L8-BO	**21.64** ±0.01	**82** ±2	**67** ±1	**18.13** ±0.02	**49** ±3	**80** ±2	**3.62** ±0.01	**11** ±1	**30** ±1
PM7-D4:L8-BO	**21.93** ±0.03	**52** ±9	**91** ±5	**18.43** ±0.02	**54** ±4	**113** ±7	**3.53** ±0.01	**15** ±4	**24** ±0*
PM7-D5:L8-BO	**21.89** ±0.03	**101** ±20	**131** ±7	**18.24** ±0.06	**44** ±12	**76** ±3	**3.59** ±0.02	**30** ±13	**28** ±6
L8-BO	**-**	**-**	**-**	**18.0**	**52**	**105**	**3.49**	**25**	**24**

d = d-spacing
I = integrated peak intensity normalized by film thickness
L_c = coherence length calculated using a Scherrer relation with K=0.9
* Range is below measurement precision

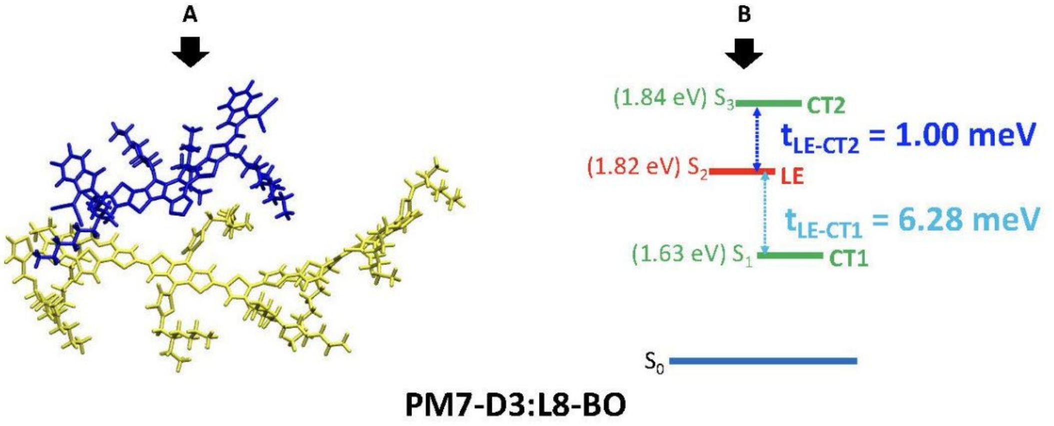

Figure S45. Illustration of A) D:A complex configuration and B) singlet energy states (LE: local-exciton state, CT*n*: n^{th} charge-transfer state, and S_0/GS: ground state) and electronic coupling values for the dimeric complex of PM7-D3 with L8-BO NFA, as calculated at the TD-DFT ωB97X-D/6-31G(d,p) level, considering an implicit dielectric medium with ε=3.5. The state energies are not illustrated to proportion.

References:

1. Jones, Austin L., et al. "Investigating the active layer thickness dependence of non-fullerene organic solar cells based on PM7 derivatives." Journal of Materials Chemistry C 8.43 (2020): 15459-15469

2. Liu, Delong, et al. "Design of wide-bandgap polymers with deeper ionization potential enables efficient ternary non-fullerene polymer solar cells with 13% efficiency." Journal of materials chemistry A 7.23 (2019): 14153-14162.

3. Li, Sunsun, et al. "Influence of covalent and noncovalent backbone rigidification strategies on the aggregation structures of a wide-band-gap polymer for photovoltaic cells." Chemistry of Materials 32.5 (2020): 1993-2003.

4. Lee, Young Woong, et al. "Regioisomeric polythiophene derivatives: synthesis and structure-property relationships for organic electronic devices." Macromolecular Research 28.8 (2020): 772-781.

5. Heuvel, Ruurd, et al. "Thermal behaviour of dicarboxylic ester bithiophene polymers exhibiting a high open-circuit voltage." Journal of Materials Chemistry C 6.14 (2018): 3731-3742.

6. Jones, Austin L., et al. "Acceptor gradient polymer donors for non-fullerene organic solar cells." Chemistry of Materials 31.23 (2019): 9729-9741.

7. Wu, Ying-Sheng, et al. "Investigation of the mobility–stretchability relationship of ester-substituted polythiophene derivatives." Macromolecules 53.12 (2020): 4968-4981.

8. Frisch, M. J. et al. “Gaussian 16, Revision C.01” Wallingford CT, (2016).

9. Shao, Yihan, et al. "Advances in molecular quantum chemistry contained in the Q-Chem 4 program package." Molecular Physics 113.2 (2015): 184-215.

10. Cave, Robert J., and Marshall D. Newton. "Generalization of the Mulliken-Hush treatment for the calculation of electron transfer matrix elements." Chemical physics letters 249.1-2 (1996): 15-19.

11. Coropceanu, Veaceslav, et al. "Charge-transfer electronic states in organic solar cells." Nature Reviews Materials 4.11 (2019): 689-707.

12. Marcus, Rudolph A. "Electron transfer reactions in chemistry. Theory and experiment." Protein electron transfer. Garland Science, 2020. 249-272.

13. You, Hoseon, et al. "Ester-functionalized, wide-bandgap derivatives of PM7 for simultaneous enhancement of photovoltaic performance and mechanical robustness of all-polymer solar cells." Journal of Materials Chemistry A 9.5 (2021): 2775-2783.

14. Baker, Jessy L., et al. "Quantification of thin film crystallographic orientation using X-ray diffraction with an area detector." Langmuir 26.11 (2010): 9146-9151.

15. Kieffer, J., et al. "New tools for calibrating diffraction setups." Journal of synchrotron radiation 27.2 (2020): 558-566.

16. Newville, Matthew, et al. "LMFIT: Non-linear least-square minimization and curve-fitting for Python." Astrophysics Source Code Library (2016): ascl-1606.

17. Zhang, Guichuan, et al. "Delocalization of exciton and electron wavefunction in non-fullerene acceptor molecules enables efficient organic solar cells." Nature communications 11.1 (2020): 3943.
